# Flexible model-based clustering of mixed binary and continuous data: application to genetic regulation and cancer

**DOI:** 10.1093/nar/gkw1270

**Published:** 2016-12-19

**Authors:** Fatin N. Zainul Abidin, David R. Westhead

**Affiliations:** 1 School of Molecular and Cellular Biology, University of Leeds, Leeds, West Yorkshire LS2 9JT, UK; 2 Institute of Systems Biology (INBIOSIS), Universiti Kebangsaan Malaysia, 43600 Bangi, Selangor D.E., Malaysia

## Abstract

Clustering is used widely in ‘omics’ studies and is often tackled with standard methods, e.g. hierarchical clustering. However, the increasing need for integration of multiple data sets leads to a requirement for clustering methods applicable to mixed data types, where the straightforward application of standard methods is not necessarily the best approach. A particularly common problem involves clustering entities characterized by a mixture of binary data (e.g. presence/absence of mutations, binding, motifs and epigenetic marks) and continuous data (e.g. gene expression, protein abundance, metabolite levels). Here, we present a generic method based on a probabilistic model for clustering this type of data, and illustrate its application to genetic regulation and the clustering of cancer samples. We show that the resulting clusters lead to useful hypotheses: in the case of genetic regulation these concern regulation of groups of genes by specific sets of transcription factors and in the case of cancer samples combinations of gene mutations are related to patterns of gene expression. The clusters have potential mechanistic significance and in the latter case are significantly linked to survival. The method is available as a stand-alone software package (GNU General Public Licence) from http://github.com/BioToolsLeeds/FlexiCoClusteringPackage.git.

## INTRODUCTION

Since clustering was first applied to microarray gene expression data for yeast (1), the use of clustered heat maps as an exploratory analysis method has been ubiquitous in the thousands of published studies covering the growing range of high-throughput genome scale data sets in molecular biology research. This is testament to the usefulness of the method, but nevertheless clustering is an art, and despite theoretical methods that quantify the tightness and separation of clusters it can be difficult to decide when a clustering solution is good or bad. Often the clustering solutions chosen in a study are those that yield the most biological insight from the data. Accordingly many different approaches to clustering exist, including standard generically applicable methods like hierarchical clustering and K-means, probabilistic model based approaches and methods specifically tailored to particular data types and problems (e.g. model based clustering for RNA sequencing data (2)). There is no consensus on the best method to apply to a given problem.

Increasingly emphasis in the field is shifting to the integration of multiple genome scale data sets. For example alongside gene expression, studies of cell lines and differentiation (3,4) now often include epigenetic chromatin marks and transcription factor binding, and similarly tumors can be characterized in terms of somatic mutations, copy number aberrations and methylation patterns. In these cases biological insight may still be obtained by clustering, but the entities are characterised by variables of mixed types, so the problem is more complex and less likely to be addressed well by generic methods. A particular challenge is that different data types may contain more or less information, some may be biologically irrelevant, and each has different characteristic levels of random variability (noise). While separate clustering of different data types followed by comparison of resulting clusters can be useful, it can ignore valuable joint information between data types that drives the underlying biology, and this motivates the development of new methodology.

A number of different approaches to clustering entities described by variables of mixed type are now becoming available. For example, Morlini (5) developed an approach to cluster mixed binary and continuous data by treating binary variables as generated from latent continuous variables with a threshold for dichotomy, leading to clustering in a multivariate normal distribution framework. These ideas were extended to ordinal and nominal variables by McParland and Gormley (6). In each case, an expectation maximization framework was adopted, requiring manual specification of cluster numbers, and methods were applied to problems with relatively small numbers of variables (<10 continuous and <20 other). Similar ideas have been explored by Browne and Nicholas (7) and by Cai and co-workers in a Bayesian context (8). Alternatively, addressing problems with large numbers of variables of different types and incorporating dimension reduction as an integral component, iCluster (9) and iPF (10) were developed specifically for integrating and clustering mixed genome-scale (‘omics’) data for disease subtype discovery. In iCluster the link between data types is achieved by assuming a shared underlying latent variable model representing the disease subtypes. A different approach is taken the Bayesian MDI package (11,12), which couples clustering based on each separate data type by linking coefficients specifying the allocation of entities to specific components in a mixture distribution.

Many problems of current interest in our field involve clustering entities described by a mixture of binary and continuous variables: for example, genetic regulation can be described in terms of the presence or absence of transcription factor binding and histone marks in promoter and enhancer elements and this can determine patterns of gene expression (13); equally, tumors can be characterized by the presence or absence of somatic mutations in key signalling genes that may drive downstream changes to oncogenic gene expression patterns. We therefore set out to develop a method to cluster such entities that would be generically applicable to a range of different problems. Our specific goals were a method able handle variable numbers and data set sizes common in the field, and where the optimum number of clusters is unknown and difficult to estimate, making manual experimentation impractical. We sought a method that would give clusters with clear biological interpretability, for instance a pattern of mutation or TF binding that relates to a shared pattern of expression in a cluster of genes, and therefore avoided using dimension reduction as an integral component, assuming that this would be employed at the data preparation stage if necessary to identify the most relevant variables. Satisfying these requirements led to a method complementary to those discussed above that we show to be applicable to several realistic current problems.

We chose a simple model based framework, using a joint probability distribution of binary and continuous variables that is a mixture over an unknown number of clusters. An attractive feature of this probabilistic approach is that it provides a natural treatment of data sets where some variables may be irrelevant, for example passenger mutations in cancer samples or transcription factor binding to DNA without regulatory significance, and where there may be false positives and negatives, for example in chromatin immunoprecipitation data. In outline the method employs a heuristic search for an approximate optimal model followed by refinement using an expectation-maximization procedure. We investigated model selection with simulated data using a range of criteria related to the well-known Akaike-Information-Criterion (AIC) (14), the Bayesian Information Criterion (BIC) (15) and their variants (16,17). The method was applied to two different problems, genetic regulation in yeast based on transcription factor binding and gene expression, and the classification of cancer samples based on somatic mutations and gene expression. This showed the method to be effective in identifying clusters that relate to relevant biomolecular mechanisms, and in the cancer case to survival.

## MATERIALS AND METHODS

### Model

We consider a set of *N* entities (data points) *i*, representing genes, tumor samples etc., each characterised by ${r_{ij}} \in \{ {0,1} \}, j = 1, \ldots ,{n_r}$ binary variables and ${e_{il}}, l = 1, \ldots ,{n_e}$ continuous variables. For example the binary variables could indicate the binding or not of a transcription factor in a gene promoter or the presence/absence of a mutation at a particular locus (we allow for *n_r_* such variables), and the continuous variables could be gene expression values in *n_e_* samples, experiments or time points. We assume a probability distribution which is a mixture of *N_m_* components (clusters)

\begin{eqnarray*}\begin{array}{@{}*{1}{l}@{}} {p({r_{i1}}, \ldots ,{r_{i{n_r}}},{e_{i1}}, \ldots ,{e_{i{n_e}}}) = }\\ {\sum\limits_{m = 1}^{{N_m}} {{\alpha _m}} \prod\limits_{j = 1}^{{n_r}} {B({r_{ij}};{p_{mj}})} \prod\limits_{l = 1}^{{n_e}} {N({e_{il}};{\mu _{ml}},{\sigma _{ml}})} } \end{array}\end{eqnarray*}

from which data points are assumed to be generated. Here ${\alpha _m}$ are mixing coefficients ∑ α*_m_* = 1; *B* denotes the Bernoulli distribution with parameter ${p_{mj}}$, and *N* is a normal distribution with parameters ${\mu _{ml}}$ and ${\sigma _{ml}}$. In the case of genetic regulation the mixture components represent the well-known concept of a cluster of co-regulated genes, with, for example, Bernoulli parameters ${p_{mj}}$ representing the probability of binding for particular transcription factors in promoter/enhancer elements, and the ${\mu _{ml}}$representing a shared average pattern of gene expression, which could be a time or developmental series but is not required to be. In the case of tumour samples, clusters could be related samples where Bernoulli parameters associate mutation probabilities at particular loci with shared patterns of oncogenic gene expression.

### Estimating model parameters

Since the number of clusters is unknown and difficult to estimate we adopted an initial heuristic search for an approximately optimal model, followed by refinement of the solution by expectation maximization. The heuristic search employed a Monte-Carlo simulated annealing algorithm (18) to optimize objective functions of the form

\begin{equation*}O \left( {L,k} \right) = - 2L + k\lambda \left( N \right)\end{equation*}

where *L* is the (maximized) log-likelihood from the distribution above, $\lambda$ is a function of the number of data points *N* and *k* is the number of parameters in the model.

We investigated several different functions $\lambda ( N )$, including constants ($\lambda = 1.0 - 5.0$) where $\lambda = 2$ corresponds to the standard AIC criterion, $\lambda ( N ) = \ln N$ (the BIC criterion), the Hannan-Quinn criterion $\lambda ( N ) = 2\ln \ln N$ and the consistent AIC (CAIC, $\lambda ( N ) = 1 + \ln N$). Regarding the choice of objective functions the standard AIC and BIC are the most commonly used, and they arise from fundamentally different theoretical stand points (19). The AIC is obtained by minimizing the Kullbeck–Liebler distance between the estimated model and an underlying ‘true’ model, while the BIC maximizes the posterior probability of the model given the data. Both criteria are asymptotic results applicable to large samples, but their large sample behaviour is fundamentally different: the BIC is asymptotically consistent (converges in probability to a single model as $N \to \infty$) and AIC is not. AIC on the other hand embodies the idea that as the data set grows in size evidence may emerge for a more complex model. We note that in our case we are likely to be some way short of the large sample limit, and that the choice of objective function is likely to be based on empirical considerations. The rationale for investigating different multipliers in the AIC type criteria (with no *N* dependency in the penalty) was that for small samples the standard AIC is an underestimate of the optimal penalty, but a more complex correction is not suitable numerically for our approach.

Full details of the heuristic search algorithm and equations for expectation maximization are given in Supplementary information, along with details of how the algorithm was parametrised using simulated data. Algorithm parameters in the form of input files for the clustering program for the two biological test cases are also provided in supplementary information.

### Test data sets

As an initial test of the methodology and objective functions we examined their ability to find correct solutions for the number of mixture components and the assignment of data points to components in data simulated from the probability distribution above. Simulated data was also used to parametrize the algorithms (see Supplementary Data). For the tests described here, we simulated data sets with 100, 200, 500 and 1000 data points, and for each case two data sets comprising clusters of equal sizes of 10 or 20 (e.g. the 500 data point sets were 1. 25 clusters of size 20 and 2. 50 clusters of size 10). All data sets had ${n_r} = 20$ and ${n_e} = 20$ and each cluster was specified with a distinct pattern of binary variables ${p_{mj}}$ and continuous variables ${\mu _{ml}}$ (randomly chosen) and ${\sigma _{ml}}$. One set of simulations modeled the case of tight well separated clusters with low noise, and the second set modeled less well separated clusters with a higher level of noise. In the first set the values of ${p_{mj}}$ were either 0.9 or 0.1 and all ${\sigma _{ml}}$ values were 0.01, in the second set the values of ${p_{mj}}$ were either 0.9 or 0.4 and all ${\sigma _{ml}}$ values were 0.3. Random numbers from appropriate probability distributions were generated using standard functions in the Java programming language.

### Data for genetic regulation

To test the methodology in application to genetic regulation we used the well-studied yeast cell cycle, basing our work on gene expression data (18 time-points) from Spellman and co-workers (20) and 103 yeast transcription factors (TFs) from the regulatory map published by Harbison *et al.* (21). In the regulatory map, a TF was assumed to bind if the *P*-value was less than 0.001. Based on our studies with simulated data we considered that our method is suitable for a data set of a few hundred genes and 10–20 regulatory inputs, and this is consistent with estimates from previous studies of the number of genes showing cell cycle related expression and the likely number of TFs involved in cell cycle related regulation (22–24). Accordingly, we began with 525 genes identified by the authors as showing cell cycle related expression. Our preselection of TFs was based on the preselection step for the LeTICE algorithm (25). This is based on the hypothesis that if a TF is active in regulating any of the selected genes, then within the set of genes whose promoters it binds there should be some gene pairs showing highly correlated expression patterns reflecting common regulation, even allowing for the possibility that the TF does not regulate all the genes that it binds. Therefore using the 95th percentile, ρ, of Pearson correlation coefficients over all gene pairs, the proportion of correlations greater than *ρ* in the gene set that the TF binds is calculated. This is then compared to the proportion of correlations greater than *ρ* in randomly selected gene sets of the same size, and an empirical p value calculated. If this p value is less than the generous threshold of 0.1 then it is assumed that the TF may regulate some genes and it is retained, otherwise the TF is removed from the set under consideration. In this case 17 TFs were retained for input to the main clustering algorithm, on the assumption that these TFs are the ones likely to be regulating cell cycle genes. Following this the set of 525 genes was further reduced to 328 by eliminating genes not bound by any of the selected TFs.

LeTICE (25) was also used as an alternative method for comparison with our approach. LeTICE is not a generic clustering method but is designed specifically for the problem of genetic regulatory network prediction. It is based on integrating TF binding data with expression pattern data to define a genetic regulatory network, i.e. a set of modules each comprising genes with a common TF binding pattern and a shared pattern of expression. This is achieved by finding the network, *B*, which maximizes $P( {B{\rm{|}}L,E} )$where *L* is a matrix of TF binding probabilities and *E* a matrix of gene expression patterns. As such LeTICE is a method based on a similar premise of integrating TF binding data and expression data to find regulatory relationships, but being based on different underlying methodology it is an ideal comparator, albeit only relevant to the problem of genetic regulation. To provide a direct comparison of algorithms, LeTICE was applied to the dataset described above. Note that LeTICE takes binding *p* values directly as input and that it has its own TF and gene pre-selection criteria, in this case it selecting 18 TFs and 289 genes. LeTICE was then run with the optimum runtime parameters suggested in the original paper.

As part of this study we also examined the effect of using normalized (where each gene was normalized to zero mean and unit standard deviation) and un-normalised gene expression data. We also compared joint clustering to clustering expression data separately, which can be done by simply omitting binary variables in the input to our program.

In evaluation of our method we considered comparison with the known literature on genetic regulation in the yeast cell cycle, as well as measures of the functional coherence of clusters based on Gene Ontology (GO) using the GOSemSim (26) package in R (with the information content based semantic similarity measure). Since our method can identify combinatorial regulation (a cluster of genes regulated by more than one TF), and this implies potential interactions between TFs, we also compared these implied interactions with physical and genetic evidence in BioGRID (27). As for selecting a set of relevant regulators related to the well-known cell cycle regulators in the discussion section, KEGG was used to retrieve all genes related to the cell cycle pathway (28).

### Data for acute myeloid leukaemia

To test the application of our methodology to data from cancer samples, we applied it to the Acute Myeloid Leukemia (AML) mutation and gene expression data generated by The Cancer Genome ATLAS (TCGA) Research Network (29,30). This is an effective test since classification of AML samples has been the subject of extensive research which can be compared to our results, including the French-American-British (FAB) system which largely relies on cell histopathology, the World Health Organisation classification which includes cytogenetic aberrations, and further work using gene expression alone (31–33), gene mutations (34) and linking gene mutations to expression (35).

Datasets from TCGA were retrieved through using cBioPortal for Cancer Genomics tool (36,37). In the TCGA data, samples were selected based on the availability of mutation and RNA-seq gene expression data. Genes which were mutated in at least two patients were chosen and samples with no mutation were removed, resulting in 170 samples and 154 gene mutations. For gene expression, we chose the 500 genes with highest ranked-based coefficients of variation and standard deviation across these samples (details of samples, mutations and chosen genes are given in Supplementary Data). Up and down regulated genes in each cluster were analysed using GenePattern 2.0 (38).

## RESULTS

### Simulated data

**Figure 1.**
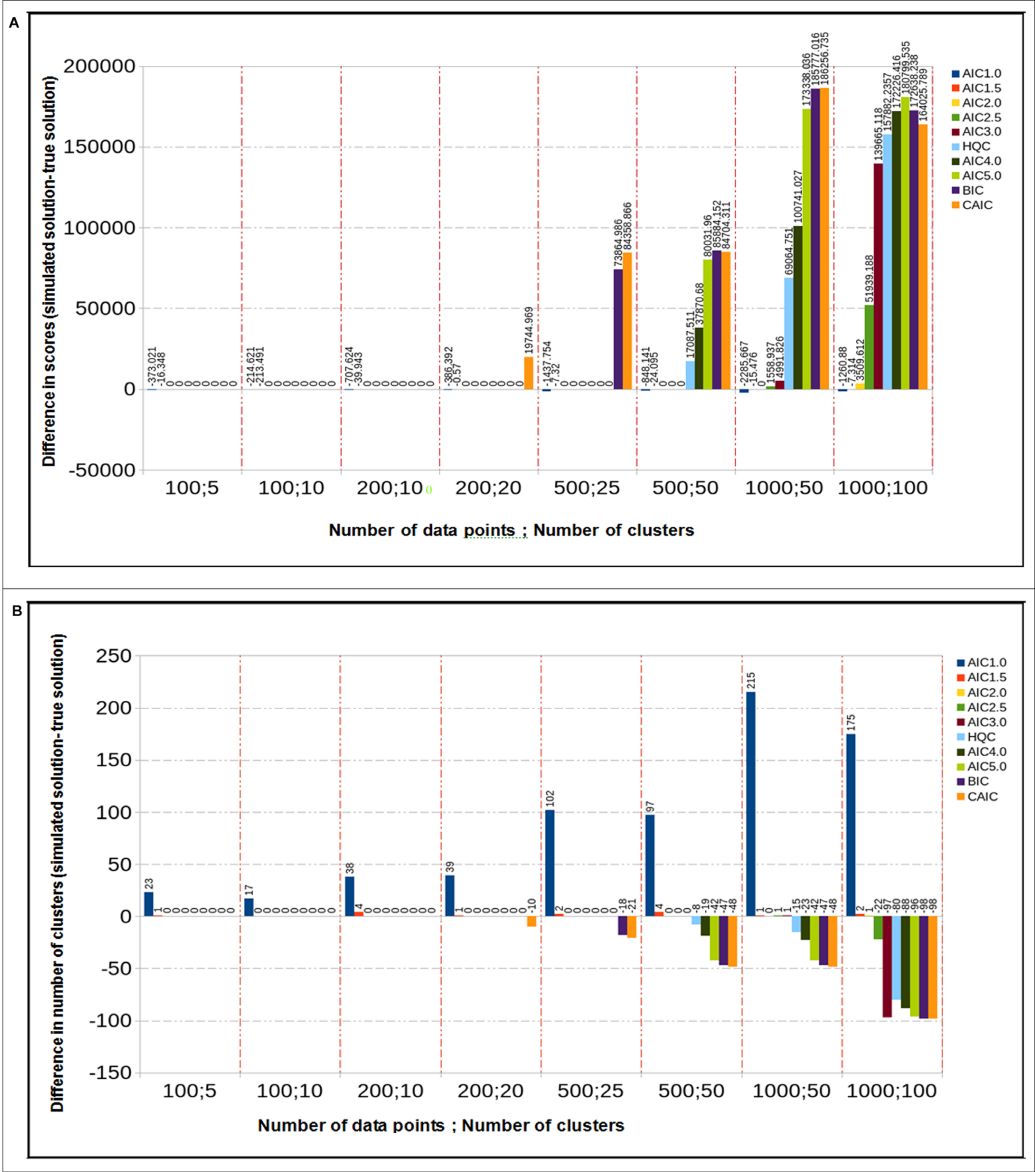
Result from our algorithm using a data set simulated from the probability distribution assumed in the paper for ${n_r} = 20$ binary variables and ${n_e} = 20$ continuous variables. In this case, parameters of the simulation correspond to tightly clustered data and relatively little ‘noise’ (Bernoulli parameters of 0.1 or 0.9 at each regulatory input and expression standard deviations of 0.01). Cases simulated covered 100–1000 data points and 10 or 20 data points per cluster in each case. Panel (**A**) shows the difference in score, and panel (**B**) the difference in the number of clusters, between the solutions found by the algorithm and the known true solutions. Results are shown for several objective functions arranged in order of increasing penalty value λ. Differences of zero in each case indicate that the algorithm found the true solution; negative score differences indicate objective function failures (solutions different to the true solution exist with better scores), and positive score differences indicate search algorithm failure (algorithm stopped at a solution scoring worse than the true solution).

The results of applying the method to simulated data, using 100–1000 data points and 5–100 mixture components are shown in Figure 1 for simulated data with a low level of variability (tight, well separated clusters) and in Supplementary Figure S1 for data with higher variability and less well-defined clusters. Figure 1 shows that for smaller numbers of data points (100–200) the optimization algorithm successfully finds the correct solution and that this is relatively insensitive to the chosen objective function. Only the very low penalty functions ($\lambda = 1.0,1.5)$ generate solutions with lower objective values and more mixture components than the underlying distribution from which the data were generated. With more data points to cluster the optimization procedure finds solutions equal or very similar to the correct solution for $\lambda = 2.0,2.5$, encompassing the standard AIC and slightly higher penalties, which might be expected on theoretical grounds. However for stronger penalties, including those with *N* dependency, solutions with higher objective function values and too few clusters are found. We note that this seems to be a failure of the optimization method rather than the objective function, and suggest that it reflects optimization on a surface where the likelihood gives limited ‘downhill’ information compared to the strong penalty on parameter numbers. Using data simulated with higher variability and less well defined clusters (Supplementary Data) leads to similar conclusions: values of $\lambda = 2.0,2.5$ yield the best solutions over a range of problem sizes. In this case, some of the higher penalty criteria fail at the level of the objective function (solutions with too few clusters have lower objective values than the correct solutions) rather than optimization method. Overall, these results with simulated data suggest that the method is most successful with AIC type objective functions without N dependency on the penalty term, and that the actual AIC ($\lambda = 2.0)$ is an effective choice with simulations suggesting the use of slightly higher penalties for small data sets. Further work with larger simulated data sets (not shown) gave similar results, suggesting as expected that $\lambda$ values close to 2.0 should be used.

### Application to genetic regulation in the yeast cell cycle

**Figure 2.**
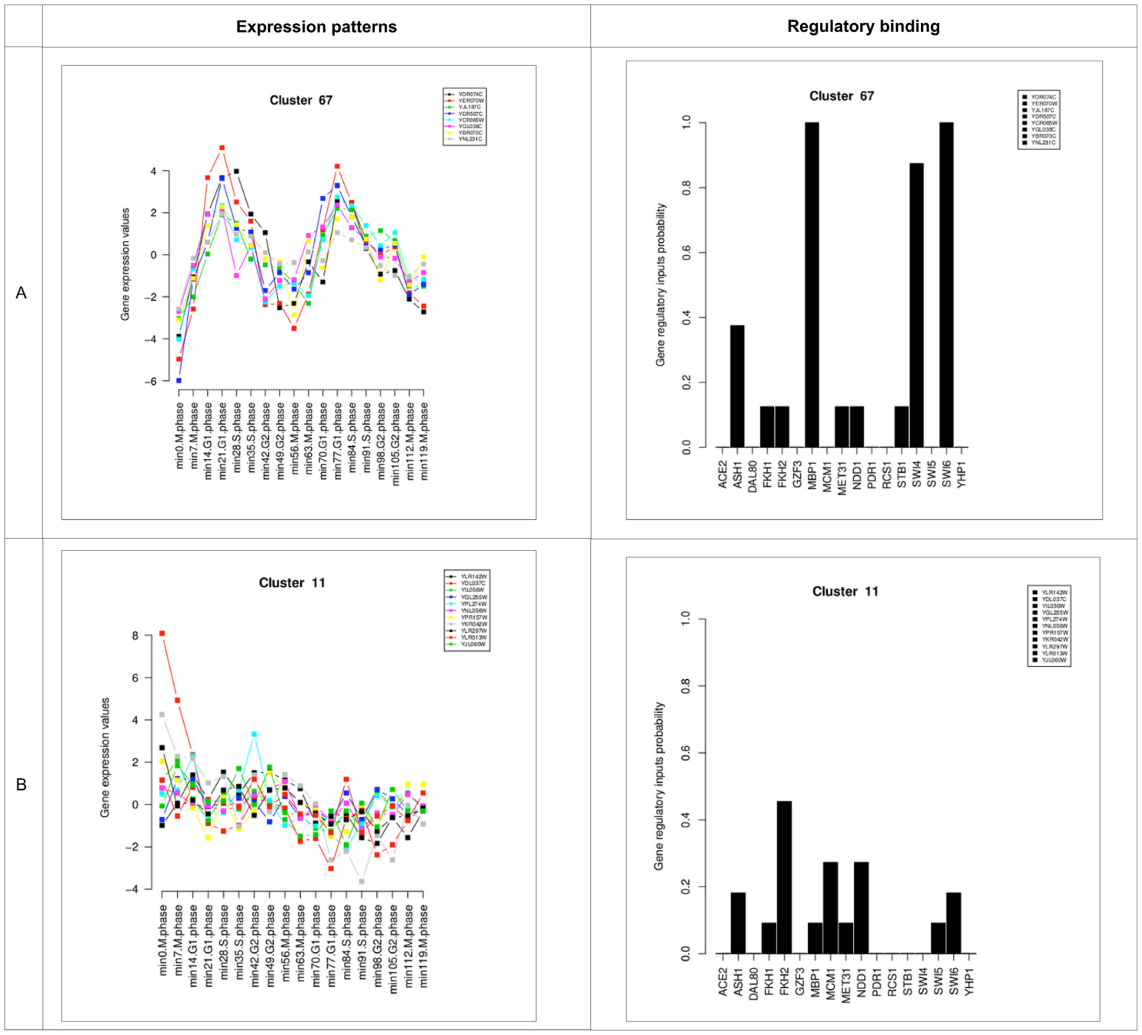
Two examples of clusters showing both expression patterns and regulatory binding patterns generated using the standard AIC objective function and normalized gene expression patterns. Panel A shows expression and regulatory binding patterns plots for genes in cluster 67: here a clear gene expression pattern is associated to a clear regulatory hypothesis involving high probability binding by Mbp1, Swi4 and Swi6. On the contrary, a clear regulatory hypothesis could not be made for cluster 11 in panel B: these genes do not have a very clear cell cycle expression pattern nor do they show a high probability of binding any transcription factor.

We applied our method to the cell cycle data using parameters suggested from the simulation study. Some example clusters are shown in Figure 2 and all clusters are in Supplementary Data, in these cases employing the standard AIC ($\lambda = 2.0)$. We also examined the effect of refinement of the clusters using the EM algorithm and results for the marginal densities are shown in Supplementary Data: these results reveal little overlap of the clusters: no genes have significant probability of membership of clusters other than the one assigned by the heuristic search and parameter estimates for the clusters did not change significantly after refinement. Figure 2A illustrates cluster 67, within which genes have a clear cell cycle related expression pattern and linked regulation by three TFs with high probability, corresponding to a clear regulatory hypothesis for this group of genes. On the other hand Figure 2B shows a cluster with no clear TF binding or gene expression pattern, and this case we consider that the cluster contains limited information about the expression and regulation of the corresponding genes. Based on these observations, we chose to analyse our data by first extracting clusters where the expression and binding pattern is clear (average Pearson correlation of expression patterns > 0.5 and at least one TF with binding probability > 0.5). In this case, of the 76 clusters produced (Supplementary Data), 52 met these criteria and we refer to these subsequently as ‘clear’ clusters.

In Table 1 we show some statistics of different approaches to clustering this data set, where the results in Figure 2 ($\lambda = 2.0)$ correspond to the first column. The number of clusters found by each method follows expected patterns: using a stronger penalty term ($\lambda = 2.5)$ results in fewer and larger clusters, although this effect is more pronounced that it was in the simulated data examples. Normalizing gene expression also results in fewer clusters. Clustering with expression data only results in fewer larger clusters, indicating that gene expression patterns may be shared when regulation is different and underlining the advantage of a joint clustering approach incorporating regulation. Regarding the results from LeTICE, it should be appreciated that this method assigns genes to ‘regulated modules’ or to the ‘background’, a process that corresponds to our approach of focusing on clusters with clear regulation and gene expression. The 14 clusters produced by LeTICE can be viewed therefore as similar to our results for clear clusters using ($\lambda = 2.5)$ and normalized expression data (15 clusters).

**Table 1.** Statistics of clusters found by joint clustering of regulation and expression with different objective functions AIC (λ = 2.0) and AIC2.5 (λ = 2.5), with and without normalization of gene expression, compared to using LeTICE and using expression alone. Gene symbols in red are the nine well known yeast cell cycle transcription factors

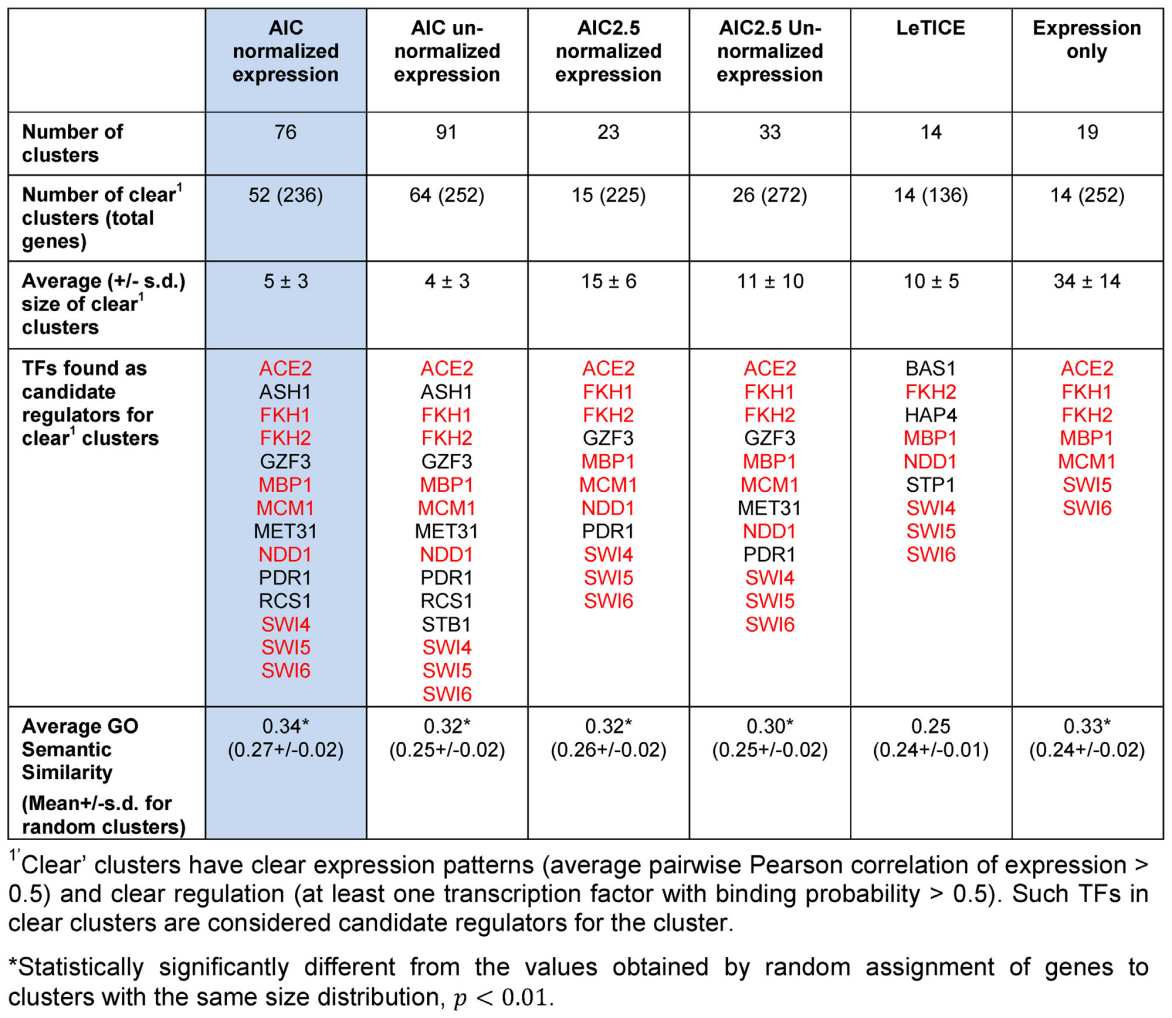

We also considered which of the TFs were assigned significant regulatory roles, i.e. which TFs appear with binding probability >0.5 in at least one ‘clear’ cluster. As expected these were more numerous in methods that produce more and smaller clusters. We observe that all nine of the well-known yeast cell cycle TFs (23), were assigned regulatory roles by our methods using any objective function tested, and with LeTICE this was not the case. Equally in expression only clustering fewer TFs were assigned roles, again emphasising that genes may be co-expressed with different regulation.

We measured functional coherence of the clusters using the average semantic similarity of Gene Ontology annotations of the clustered genes (Table 1). By this measure, most methods produce clusters that are significantly better than a random assignment of genes to clusters of the same size distribution. For neither AIC type objective function is there any strong evidence of a difference in results based on normalisation of the expression data. Finally, based on GO criteria and the implication of more TFs in regulatory roles, we marginally preferred AIC ($\lambda = 2.0)$ with normalised expression and our subsequent analysis is based on these clusters.

**Figure 3.**
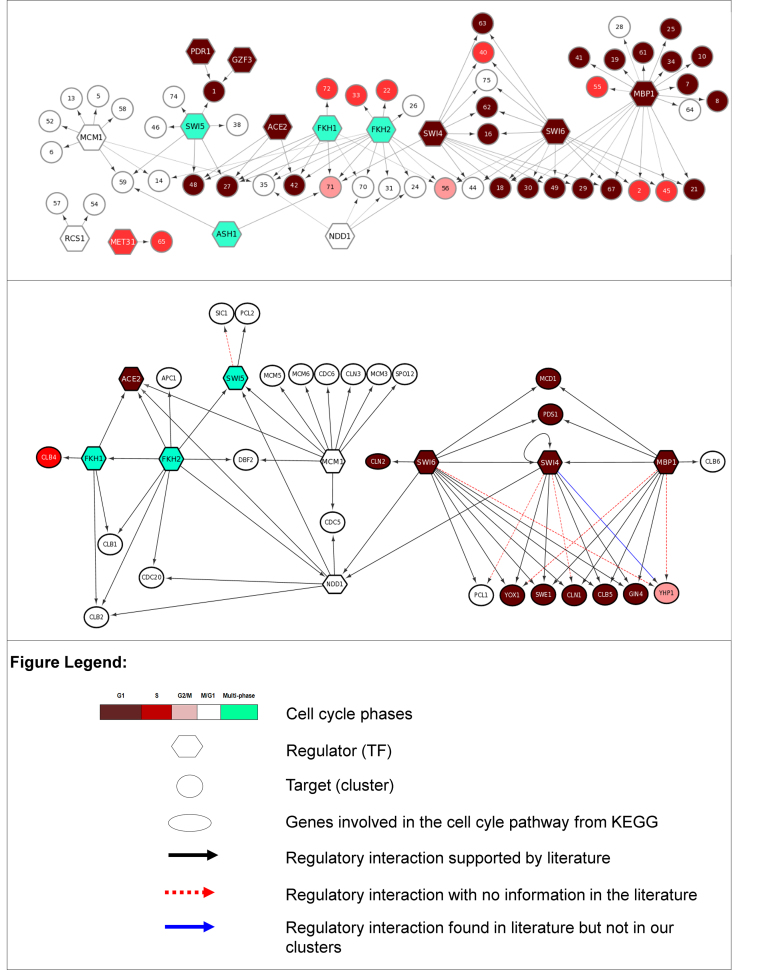
*Top*. The transcriptional regulatory network obtained from clusters with clear regulation and expression using the AIC objective function. The hexagonal nodes represent transcription factors and circular nodes regulated clusters (labeled 1–76, only clear clusters shown): colors represent cell cycle phases (peak expression phase for clusters, and the main phase of the regulated clusters for each transcription factor). *Bottom*. The regulatory network of transcription factors and other regulators extracted from the above network. Transcription factors shown are those associated by our algorithm to the regulation of clear clusters; other cell cycle regulators were identified in our gene set and overlapped with cell-cycle pathway map in KEGG (19).

The regulatory networks implied by the clustering are shown in Figure 3, first connecting TFs to their regulated clusters (upper panel) and then focusing on connections between all TFs and known cell cycle regulators (lower panel). It is notable that our choice of AIC as objective function produces a relatively large number of clusters, some of which are quite small. However, we note that even very small clusters, for example clusters 40, 42, 47, 48 and 49 containing 2–3 genes each, have clear regulation (see Supplementary Data) and contain genes with related functions. These clusters have statistically significant functional enrichment in cellular budding (clus. 48), drug transport (clus. 47), chromatin assembly and disassembly, cell wall organisation (clus. 40), response to pheromones and sexual reproduction (clus. 42). Equally, there are often several clusters which are related in expression and regulation, for instance clusters 18, 29, 30, 49 and 67 whose expression patterns all peak in G1 phase and all show a high probability of regulation by the TFs SWI4, SWI6 and MBP1. These separate clusters have clearly different GO annotations: regulation of transcription (clus. 18), organelle fission and nuclear division (clus. 29), conjugation with cellular fusion (clus. 30), regulation of protein kinase activity/cell division/bud site selection (clus. 49) and deoxyribonucleotide biosynthetic processes (clus. 67), and their separation reflects differences of detail in the expression pattern and regulatory probabilities.

The regulation of the yeast cell cycle has been extensively studied both experimentally and in the context of algorithms aimed at reconstruction of the network from different sources of data (see (39) for a recent review). Here, we offer a very brief discussion of our results in that context. The regulatory relationships in Figure 3 are largely known, and most of the regulatory relationships in the lower panel are supported, as shown, by evidence from the literature. We have already commented on regulation of G1 phase genes by SWI4/SWI6/MBP1 which together form the heterodimeric transcription factors MBF and SBF, and note that our method also finds the known regulation of G1 phase cyclins CLN1, CLN2, CLB5, CLB6, SWE1 and GIN4 by these factors. Regulation of S phase genes also by SBF and MBF (40), particularly histones and genes associated with chromatin organization, is evident in clusters 2, 40 and 55, while other S phase clusters (clus. 33, 72) are regulated by FKH1 and FKH2. Moving to G2M and M phase, while SBF/MBF still participate in regulation, it becomes dominated by MCM1, NDD1, FKH1 and FKH2. In particular our algorithm finds regulation of the key cyclins CLB1 and CLB2 by FKH1/2 and NDD1, but does not discover known links to SBF or MCM1 (41,42). We note also the interesting disconnected component in Figure 3, cluster 65 (see Supplementary Data) being regulated by MET31, comprising genes associated with S-adenosylmethionine metabolism which has been linked to cell cycle control (43,44).

Combinatorial regulation of genes by multiple TFs is known to be important and several of our clusters exhibited high probability binding by more than one TF (e.g. regulation by SWI4, SWI6 and MBP1 in Figure 2A). Such multiple regulation implies possible interaction between the factors concerned and in Supplementary Data we summarise genetic and physical interaction evidence supporting combinatorial interactions in our clusters. All but one identified combinatorial interaction is supported by some evidence from BioGRID (27), and most have extensive support. Finally we note that of the regulatory interactions predicted between TFs and genes within our clusters, only 34% are supported by significant correlation between those genes’ expression patterns and the expression patterns of the regulating factors. Although this percentage increases if correlations off-set in time are considered, it shows that simple correlation of expression is not a good way of predicting regulation.

### Application to acute myeloid leukaemia data

**Figure 4.**
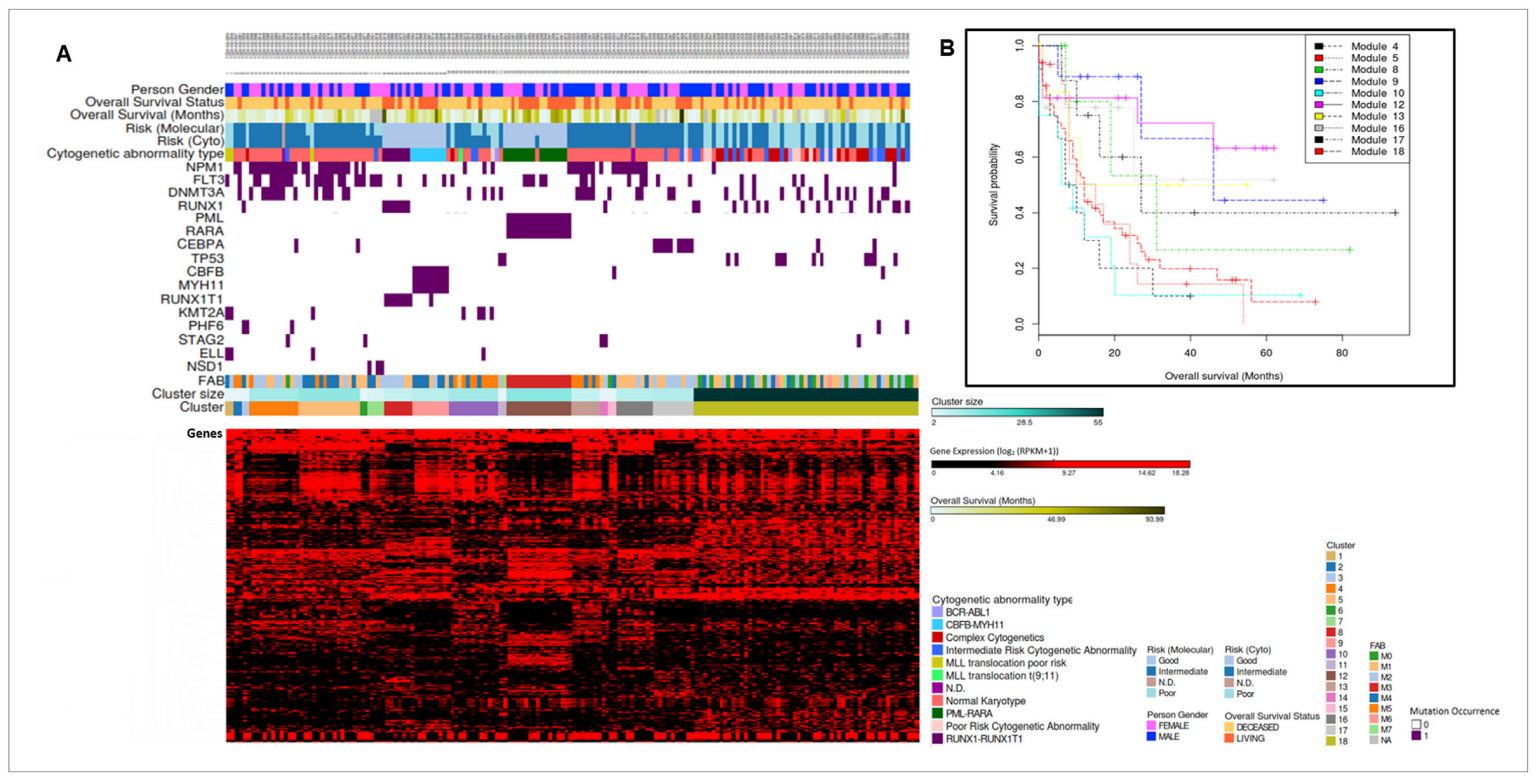
(**A**) Clustering of AML samples shown in columns of 170 samples using AIC (λ = 2.5) across the most variably expressed 500 genes (lower) and the 18 mutations (above, dark purple bar shows a mutation). Other relevant variables are also shown to aid interpretation (but were not used in clustering). (**B**) Kaplan–Meier estimators for the 10 clusters with more than two samples with survival information available in each cluster. The 10 Kaplan–Meier estimators perform differently with a significant *P*-value in the log-rank test, *P* = 0.001.

Based on our findings with simulated data, we investigated clustering of this mutation and expression data using the AIC related criteria with $\lambda = 2.0$ and 2.5. Again clustering with this real data set showed greater variability in results between these two penalty functions than was evident in simulations, with clusters predominantly very small (two samples) from $\lambda = 2.0$. Accordingly we chose $\lambda = 2.5$ in this case on biological grounds: the results are shown graphically in Figure 4A, and Supplementary Data lists the up- and down-regulated genes in each cluster along with the associated gene mutations and probabilities. Figure 4B shows survival curves for larger clusters. Overall, it is clear that the method is able to discover clusters of samples where characteristic mutation patterns are associated with distinct patterns of gene expression; these are potentially related to different oncogenic mechanisms and show statistically significant differences in survival.

Cytogenetic abnormalities are well known in AML with several well-known translocations and associated gene fusions, and this is evident in several of the clusters our method produces. For example cluster 12 (Figure 4A) has been identified by our method because the fused PML and RARA genes are both classed as mutated in this data, and this pair of mutations is associated with a distinct and characteristic pattern of gene expression. We note a single sample in the middle of this cluster that has these mutations and gene expression pattern but is not annotated with the cytogenetic abnormality: this appears to be an annotation error. In a similar way, clusters 1, 8 and 9 are associated with cytogenetic abnormalities and MLL (alias KMT2a)/ELL, RUNX1/RUNX1T1 and CBFB/MYH11 translocations, respectively (29), and clusters 8, 9 and 12 are also associated with survival differences in this data (Figure 4B). These clusters stand in contrast to clusters 10, 15 and 18 which have distinct patterns of gene expression but are not associated with any mutations at high probability, illustrating that the method is robust to discover gene expression based clusters without an associated mutational pattern in the data.

Perhaps more interesting than cytogenetic abnormalities are clusters where other gene mutations are co-clustered with distinctive gene expression patterns: for example cluster 17 where a distinctive pattern of gene expression is associated with mutation of the transcription factor CEBPA, and the smaller clusters 11 and 14 which are associated with mutations in TP53 and STAG2 respectively. Clusters 4, 5, 13 and 16 are all larger clusters associated with mutation of NPM1 and to a greater or lesser extent the associated dysregulation of HOX genes (45) (Supplementary Data), but nevertheless have distinctively different patterns of gene expression. Cluster 4 is very strongly associated with HOX gene up-regulation and the NPM1 mutation is coupled with high probability of both FLT3 and DNMT3A mutation. Cluster 5 is similar, with a lower probability of DNMT3A mutation (<0.5), and clusters 13 and 16 are associated with more dysregulated genes and an absence of FLT3 mutations. It has been suggested that AML arises from three complementary classes of mutation (46,47): class I mutations in tyrosine kinases including FLT3; class II mutations in transcription factors including RUNX1, CEBPA and NPM1; class III mutations in genes associated with DNA methylation including DNMT3A; and, another class associated with tumor suppressor mutations. It is noteworthy that these clusters mix mutations from different classes, which along with the different patterns of gene expression emphasises mechanistic heterogeneity within the broad class of NPM1 mutated cases. The survival analysis (Figure 4B) shows that clusters 4 and 5 have much worse prognosis than 13 and 16, suggesting that the clusters may have clinical as well as mechanistic relevance and that appropriate biomarkers would combine both mutation and gene expression information.

## DISCUSSION

The results above illustrate that a generic approach to clustering entities described by a mixture of binary and continuous variables is potentially useful in a wide range of applications with large data sets in molecular biology. While this could have been approached in several different ways, the choice of the probabilistic model has the advantage of identifying key variables in each cluster, for example associating mutations with high probability to gene expression patterns or identifying the most likely regulating transcription factors. It also provides a natural treatment of a low level of false positives and false negatives that can affect high-throughput data in both the examples given, and probably in many similar types of high-throughput biological data.

All clustering problems face the question of how many clusters. We approached this through model selection using well-known criteria and their variants, but mindful of their approximate nature and also of concerns about applicability in the case of mixture models (19) investigated these in detail using simulated data. It is interesting that this investigation concluded with a preference for criteria close to the well-known AIC, and that in both examples given this carried through to real data sets at least in generating clusters with clear biological meaning.

In the application to genetic regulation in yeast we note that the method produces results that to a large extent recapitulate existing knowledge. We chose to compare to LeTICE (25) as a recent method based on a similar premise but otherwise methodologically very distinct. In our hands and on this data set, our method produced arguably better results. However, our view is that comparison of methods should be done independently of the authors of those methods. Accordingly we do not claim better performance, but simply take this as evidence that our method performs at least as well. In this application we suggest that limitations are not methodological, but associated with the limited nature of the data. The transcription factor binding data is not resolved by time or cell cycle phase, and this limits how well any method could perform.

We view clustering as a method of exploratory data analysis that can be used to generate hypotheses based on data, illustrated in this case as hypotheses about the regulation of groups of genes or mechanistic links between mutations and gene expression patterns in cancer samples. Our method is generic and we hope applicable beyond the examples given here. For example it could be applied without modification to the presence/absence of specific mutations in genes (rather than mutated or not), to time/cell cycle resolved transcription factor binding data and to other types of continuous data.

## Supplementary Material

Supplementary Data
