# Supplementary material for: Flexible model-based clustering of mixed binary and continuous data: application to genetic regulation and cancer
Source: Nucleic Acids Res. 2016 Dec 19;45(7):e53. doi: 10.1093/nar/gkw1270 (PMC5399749; doi:10.1093/nar/gkw1270)
Supplement: Supplementary Data [file gkw1270_supplementary_data.zip › nar-02952-met-n-2016-File011.docx]

| **Double** | **Triple** | **Quadruple** |
| --- | --- | --- |
| Mbp1-Swi6  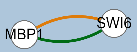  Fkh2-Mcm1  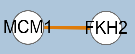  Swi4-Swi6  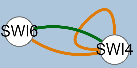  Mbp1-Swi4  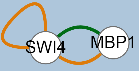  Fkh2-Ndd1  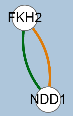 | Gzf3-Pdr1-Swi5  Mbp1-Swi4-Swi6  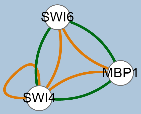  Ace2-Fkh1-Fkh2  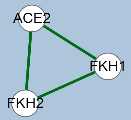  Fkh2-Swi4-Swi6  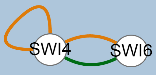  Fkh1-Swi5-Ace2  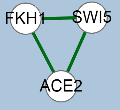  Fkh1-Fkh2-Swi6  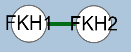  Ash1-Mcm1-Swi5  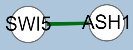 | Ace2-Fkh1-Fkh2-Swi5  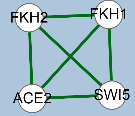  Fkh1-Fkh2-Mcm1-Ndd1  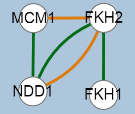  Fkh1-Fkh2-Ndd1  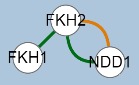  Ash1-Swi4-Fkh1-Fkh2  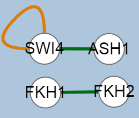 |

**Supplementary Figure S4.** Combinatorial regulatory interactions found in clusters with clear regulation and expression (the objective function is AIC and expression data was normalized). Combinatorial interactions derive from clusters regulated by more than one TF (for instance cluster 67 in Figure 2 is regulated by SWI4, SWI6 and MBP1), and are listed in each column. Below each combinatorial interaction is a figure showing the extent of support of the combination in physical and genetic interaction data in yeast from the BioGRID data base.
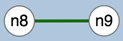
 denotes a genetic interaction and
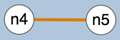
 is a physical interaction. TFs in red indicate combinatorial interactions from our algorithm that are not supported in genetic or physical interaction data (18).
