# Supplementary material for: Flexible model-based clustering of mixed binary and continuous data: application to genetic regulation and cancer
Source: Nucleic Acids Res. 2016 Dec 19;45(7):e53. doi: 10.1093/nar/gkw1270 (PMC5399749; doi:10.1093/nar/gkw1270)
Supplement: Supplementary Data [file gkw1270_supplementary_data.zip › nar-02952-met-n-2016-File007.docx]

| **A.** | 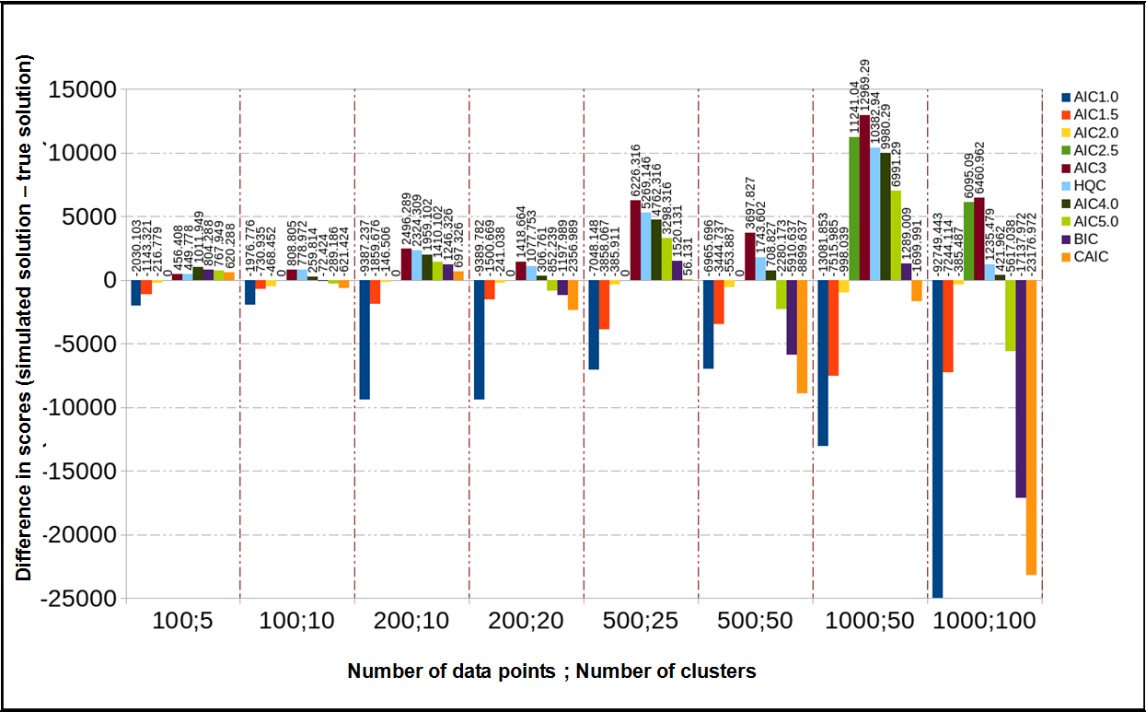  **Difference in scores (simulated solution – true solution)** |
| --- | --- |
| **B.** | 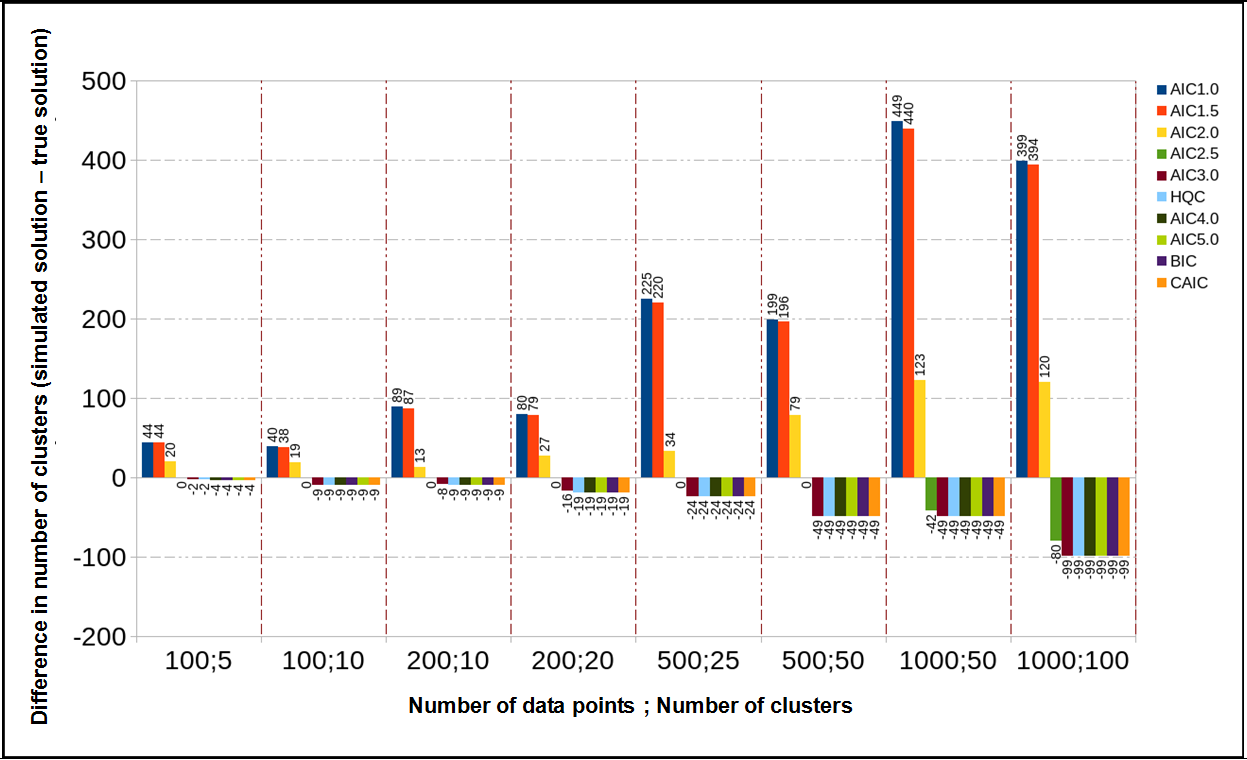  **Number of data points ; Number of clusters** |

**Supplementary Figure S1:** Result from our algorithm using a data set simulated from the probability distribution assumed in the paper for $n_{r}=20$ binary variables and $n_{e}=20$ continuous variables. In this case parameters of the simulation correspond noisier data and less tight clusters (Bernoulli parameters of 0.4 or 0.9 at each regulatory input and expression standard deviations of 0.3). Cases simulated covered 100-1000 data points and 10 or 20 data points per cluster in each case. Panel **A** shows the difference in score, and panel **B** the difference in the number of clusters, between the solutions found by the algorithm and the known true solutions. Results are shown for several objective functions arranged in order of increasing penalty value $\lambda$. Differences of zero in each case indicate that the algorithm found the true solution; negative score differences indicate objective function failures (solutions different to the true solution exist with better scores), and positive score differences indicate search algorithm failure (algorithm stopped at a solution scoring worse than the true solution).
