# Supplementary material for: Flexible model-based clustering of mixed binary and continuous data: application to genetic regulation and cancer
Source: Nucleic Acids Res. 2016 Dec 19;45(7):e53. doi: 10.1093/nar/gkw1270 (PMC5399749; doi:10.1093/nar/gkw1270)
Supplement: Supplementary Data [file gkw1270_supplementary_data.zip › nar-02952-met-n-2016-File010.docx]

**Supplementary Figure S3**

| 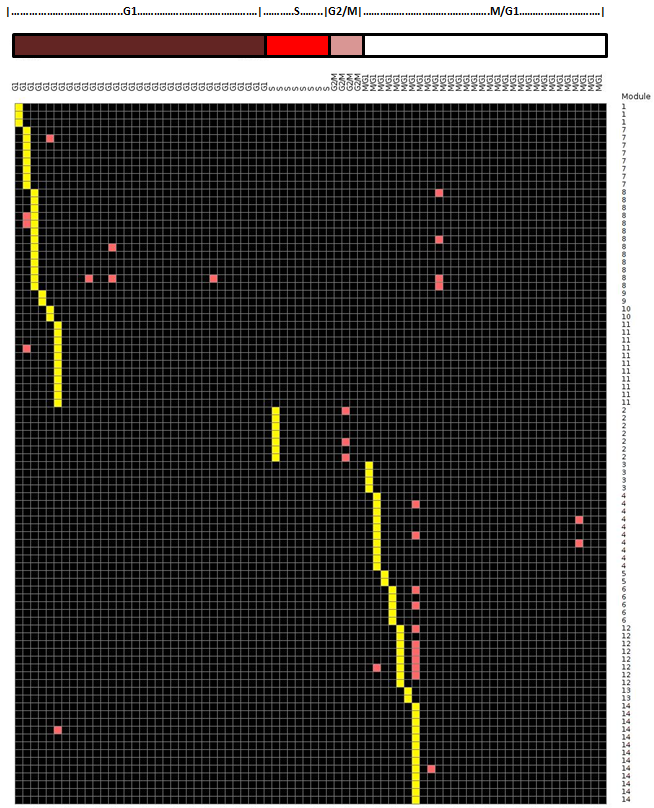 |
| --- |

**Supplementary Figure S3.** Expectation maximization results. Marginal densities $( p\left( m | i,\theta\right)$ for gene *i* and cluster *m*) are shown for the first 14 clusters found for the yeast cell cycle with the parameters corresponding to results in Figure 2 and Supplementary Figure 2. Colours: salmon (density approximately 0.0001), yellow (density approximately 1.0) and black (density approximately 0). Rows are the genes in the first 14 clusters and columns are the 76 clusters. Rows and columns are sorted by cell cycle phase showing peak gene expression in the cluster.
