# Supplementary material for: Flexible model-based clustering of mixed binary and continuous data: application to genetic regulation and cancer
Source: Nucleic Acids Res. 2016 Dec 19;45(7):e53. doi: 10.1093/nar/gkw1270 (PMC5399749; doi:10.1093/nar/gkw1270)
Supplement: Supplementary Data [file gkw1270_supplementary_data.zip › nar-02952-met-n-2016-File008.docx]

**Supplementary Figure S2**: Expression and regulatory binding patterns in the yeast data set for all clusters produced by the program using AIC as objective function with normalised gene expression (corresponding to the results and statistics reported in the first column of Table 1 in the main paper).
