# Supplementary material for: Flexible model-based clustering of mixed binary and continuous data: application to genetic regulation and cancer
Source: Nucleic Acids Res. 2016 Dec 19;45(7):e53. doi: 10.1093/nar/gkw1270 (PMC5399749; doi:10.1093/nar/gkw1270)
Supplement: Supplementary Data [file gkw1270_supplementary_data.zip › nar-02952-met-n-2016-File013.docx]

**1. Supplementary algorithmic details**

We begin with the mixture distribution, with the notation introduced in the main text of the paper, representing data generated from $N_{m}$ mixture components or clusters.

$$p\left( r_{i1},\ldots, r_{in_{r}},e_{i1},\ldots,e_{in_{e}} \right)=\sum_{m=1}^{N_{m}} \alpha_{m}\prod_{j=1}^{n_{r}} B\left( r_{ij};p_{mj} \right)\prod_{l=1}^{n_{e}} N\left( e_{il};\mu_{ml},\sigma_{ml} \right)$$

The log-likelihood is defined as

$$L=\ln\prod_{i=1}^{N} p(\boldsymbol{r}_{\boldsymbol{i}},\boldsymbol{e}_{\boldsymbol{i}})= \sum_{i=1}^{N} \ln p(\boldsymbol{r}_{\boldsymbol{i}},\boldsymbol{e}_{\boldsymbol{i}})$$

where we adopt vector notation for the binary and continuous variables for brevity, and the product is over $N$ entities or data points that are to be clustered.

**1.1 Estimating model parameters**

A standard approach to estimating the parameters in the model above would be to fix the number of mixture components or clusters (*N_m_*) and then fit the parameters of the mixture distribution by expectation maximization (EM). This process could be repeated for a selection of possible values of *N_m_* and an optimum chosen, but this procedure is difficult for a number of reasons. First we have no information from the application domain about the likely value of *N_m_*, and second the EM optimization algorithm is local in nature and therefore needs to be started from a number of different initial points to investigate possible alternative minima. Therefore we have adopted a different approach, beginning with a heuristic search and finally using EM to refine the best model found.

The heuristic search minimizes the objective functions given in the main paper,

$$O\left( L,k \right)= -2L+ k\lambda\left( N \right)$$

where k is the number of free parameters in the model. In this case k=N_m_(1+n_r_+2*n_e_)-1, accounting for N_m_-1 independent mixing coefficients, and the Bernoulli parameters and Normal distribution parameters in each model. Here *L* denotes a maximized likelihood.

The initial heuristic approach makes the assumption that the components in the mixture model above from which it is assumed the data are generated, are ‘well separated’ , so that the contribution of each data point to the likelihood is dominated by a single mixture component. With this assumption, given a solution comprising *N_m_* components and the assignment of data points to mixture components (let the set of entities assigned to component *m* be *G_m_*), the estimates of the maximum likelihood parameters for the component Bernoulli distributions are

$$p_{mj}=\frac{1}{\left| G_{m} \right|}\sum_{g_{i}\in G_{m}} r_{ij}$$

and the estimates of the parameters of the normal distributions are

$$\mu_{ml}=\frac{1}{\left| G_{m} \right|}\sum_{g_{i}\in G_{m}} e_{il}$$

$$\sigma_{ml}^{2}=\frac{1}{\left| G_{m} \right|}\sum_{g_{i}\in G_{m}} (e_{il}-\mu_{ml})^{2}$$

The estimates of the mixing coefficients are

$$\alpha_{m}= \left| G_{m} \right|/N$$

We can then seek to find model parameters by a suitable search algorithm over assignments of data points to mixture components (clusters).

**1.1.1 Heuristic search algorithm**

Even with the simplifying assumption above the search is over a very large number of possible assignments of data points to mixture components and can only be tackled heuristically. Monte-Carlo simulated annealing (SA) ([1](#_ENREF_1)) is a simple and highly effective optimization method, which is able to tackle optimization over high-dimensional and rugged (with many local minima) objective functions, and was adopted in this case. For computational convenience, solutions were represented as vectors $v$ (length $N,$ the number of data points) of component numbers ($m\in Z, 0\leq m<N$) holding the assignment of each data point to a defined component or cluster. Allowing $m$ for each data point to take any value in the specified range permits the representation of any possible solution, assigning each data point to one of a variable number of mixture components (between 1 and $N$), defining data points to be in the same mixture component if they have the same component number. The search algorithm used three Monte-Carlo moves: a random change of component for a single data point (including randomly moving a data point into a new component), randomly merging two components, and randomly splitting a component into two separate components. In contrast to the usual random starting point for SA, we considered two possible starting points: first initially placing each data point in a separate component (agglomerative clustering process) and second placing all data points in a single component (divisive clustering process).

**1.1.2 Expectation-maximization**

The parameters of model produced as the best solution from the heuristic search were refined by expectation-maximization (EM, see for example ([2](#_ENREF_2))), with the useful side effect of estimating the degree of mixing between components through the probability density that data point *i* is generated from mixture component *m*.

$$p\left( m | i,\theta\right)= \frac{\alpha_{m}p_{m}(i|m,\theta)}{\sum_{j=1}^{N} \alpha_{j}p_{j}(i|j,\theta)} .$$

This is derived from Bayes’ rule: *p_m_* is the probability density for mixture component *m*, θ denotes the (current) vector of all parameters, and the mixing coefficients α_m_ can be interpreted as prior probabilities for membership of each component. The first step of the EM algorithm is to calculate the marginal densities $p(m|i,\theta)$, using the parameters of the best solution from the heuristic search. Following this, EM updates for all the parameters can be calculated exactly from the equations that follow. For the mixing parameters

$$\alpha_{m}^{'}=\frac{1}{N}\sum_{i=1}^{N} p(m|i,\theta);$$

for the Bernoulli parameters

$$p_{mj}^{'}= \frac{\sum_{i=1}^{N} \delta_{{1,r}_{ij}}p(m|i,\theta)}{\sum_{i=1}^{N} p(m|i,\theta)} ;$$

and the Normal parameters

$$\mu_{ml}^{'}=\frac{\sum_{i=1}^{N} e_{il}p(m|i,\theta)}{\sum_{i=1}^{N} p(m|i,\theta)}$$

$$\sigma_{ml}^{'}=\frac{\sum_{i=1}^{N} {(e}_{il}-\mu_{ml})^{2}p(m|i,\theta)}{\sum_{i=1}^{N} p(m|i,\theta)} ;$$

where *θ* denotes the old parameter values, and the prime denotes the new parameter value Here $\delta_{ij}=1 \text{if }i=j \text{and} 0 \text{otherwise.}$ The *p*(*m*|*i, θ*) can then be recalculated with the new parameter values and the process iterated to convergence.

**1.2 Parametrising the algorithms**

A full list of algorithm parameters is given in Table A below. Suitable values for these parameters were determined by examining algorithm performance on simulated data. Most notably, the final result was insensitive to the starting point (agglomerative or divisive) for any reasonable annealing schedule. We found it effective to set the starting temperature high enough to achieve a high (>80%) percentage of accepted Monte-Carlo moves, adjusting the other parameters to give an annealing schedule able to find the optimal solution without consuming excessive time.

| **Runtime parameter (default)** | **Functional description** |
| --- | --- |
| NItems | Number of data points/entities to be clustered ($N$) |
| NBinary | Number of binary variables ($n_{r}$) |
| NContinuous | Number of continuous variables ($n_{e}$) |
| **Agglomerative**/Divisive | Starting point option for clustering, if agglomerative start with all data points in separate clusters, if divisive start with all in a single cluster. |
| IC (AIC/AIC$\lambda$ /BIC/HQC/CAIC) | Scoring function/information criterion for the model selection (see main paper for details). |
| StartTemp (**500**) | Starting temperature for simulated annealing. Fixed by experimentation to give a high move acceptance ratio. |
| TempFactor (**0.999**) | Factor by which the temperature is reduced at each iteration of the temperature loop. |
| MaxTemps (**1000**) | Maximum number of temperatures (termination criterion, controls ending temperature) |
| MaximumIterations (**100**) | Number of Monte-Carlo moves at each temperature |
| MergeSplitProbability (**0.25**) | There are 3 possible Monte Carlo moves, chosen according to the following scheme. A standard move (swapping a single entity into another cluster) occurs with probability 1-MergeSplitProbability. Otherwise either merging two clusters into one, or splitting one cluster into 2 are chosen with equal probability. |
| MaxRepIters (**2000**) | Maximum number of best score repetitions in SA (convergence criterion, the algorithm stops if the best solution has not changed in this number of iterations) |
| Seed (**1**) | The seed of the random number generator |
| EMIterations (**100**) | The maximum number of EM iterations if EM parameters values have not converged |
| OutInterval | Intervals at which the solution is printed in the output and at which the heat maps are updated on GUI |
| ClustFile | The name of the final clusters output file |
| NormExp (1/**0**) | Normalise continuous inputs to zero mean and unit standard deviation for each data point (if 1, otherwise don’t normalise). |
| Nrun (**0**/any integer) | Number of re-run of the program after initial run. The final clusters found by SA will be use as a new starting point for instance to carry out a more extensive heuristic search. |

**Table A:** A list of runtime parameters that were simulated in optimizing the model. Runtime parameters in red could be changed from default values (in bold) to user specified values depending upon input datasets. Other parameters can also be changed but default values are suggested to be reasonable.

**1.3 Termination and convergence criteria for SA and EM.**

There are a termination and a convergence criterion in both SA and EM. The termination criterion in SA is the maximum temperature or MaxTemps in the runtime parameters as in Table 1 whereas, the convergence criterion is the maximum number of best score repetition (MaxRepIters in Table 1). The SA will run until the temperature loop has terminated (MaxTemps) or the best score does not improve for a specified number of times (MaxRepIters). The convergence criterion in EM is if the difference in *p*(*m*|*i, θ*) for current and previous step and termination criterion for EM is similar to SA where after specified number of iterations reached, the EM calculation will be terminated (EMIterations in Table1).

**1.4 Input files for examples in the paper**

The files input-AML.txt and input-yeast.txt are the input files that should be used to generate the results discussed in the paper. These contain both algorithm parameters and data and should be used with the command line version of the program.

1. Kirkpatrick, S., Gelatt, C.D. and Vecchi, M.P. (1983) Optimization by Simulated Annealing. *Science*, **220**, 671-680.

2. Bilmes, J. (1997), *A Gentle Tutorial of the EM algorithm and its application to parameter estimation for Gaussian mixture and hidden Markov models*. International Computer Science Institute, University of California Berkeley.
