# Supplementary material for: Flexible model-based clustering of mixed binary and continuous data: application to genetic regulation and cancer
Source: Nucleic Acids Res. 2016 Dec 19;45(7):e53. doi: 10.1093/nar/gkw1270 (PMC5399749; doi:10.1093/nar/gkw1270)
Supplement: Supplementary Data [file gkw1270_supplementary_data.zip › SuplementaryFigureS2.pdf]

Expression patterns	Regulatory input patterns
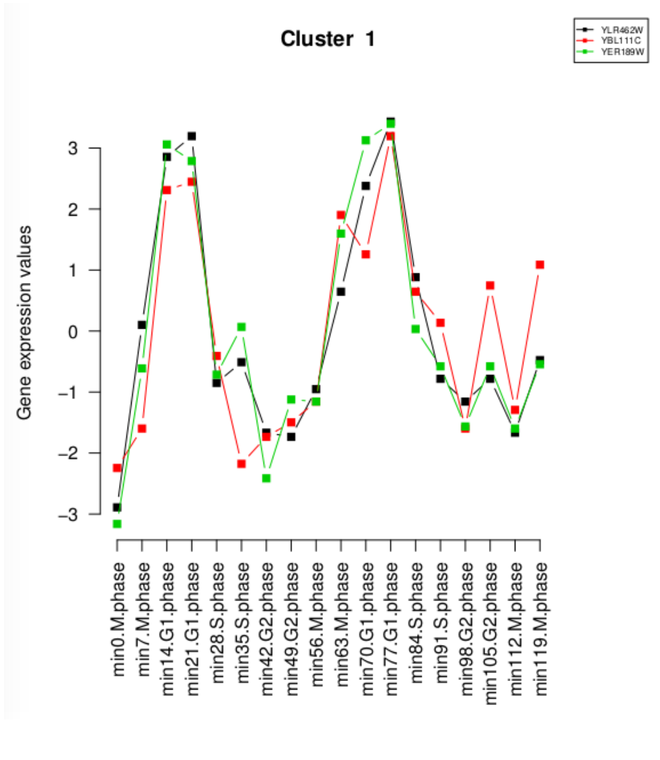	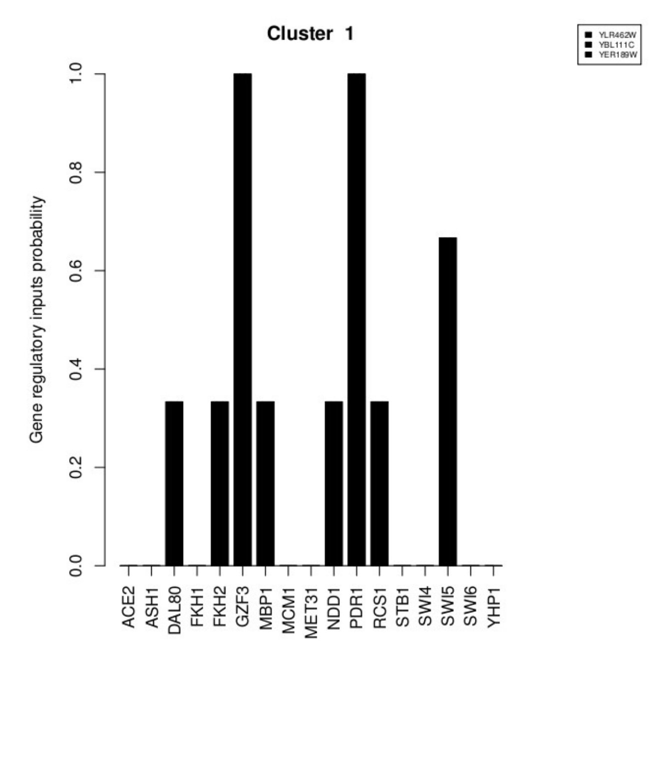
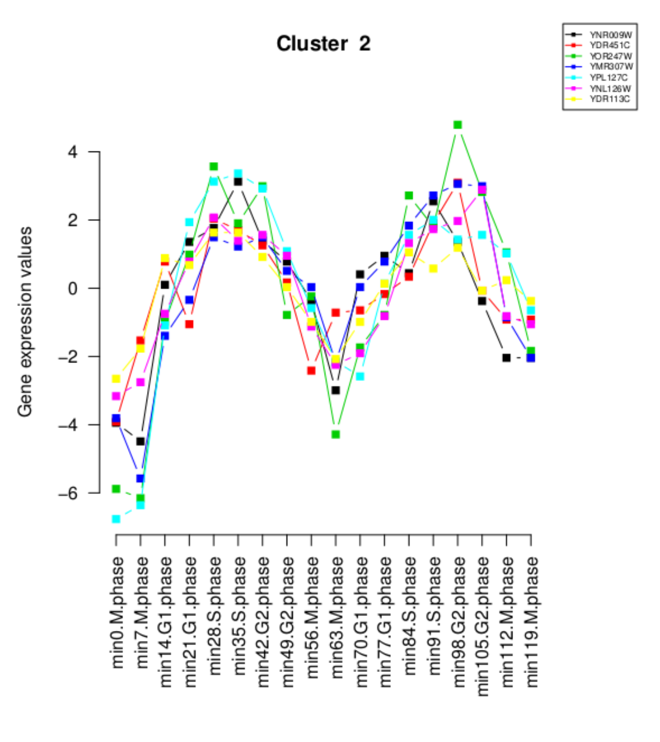	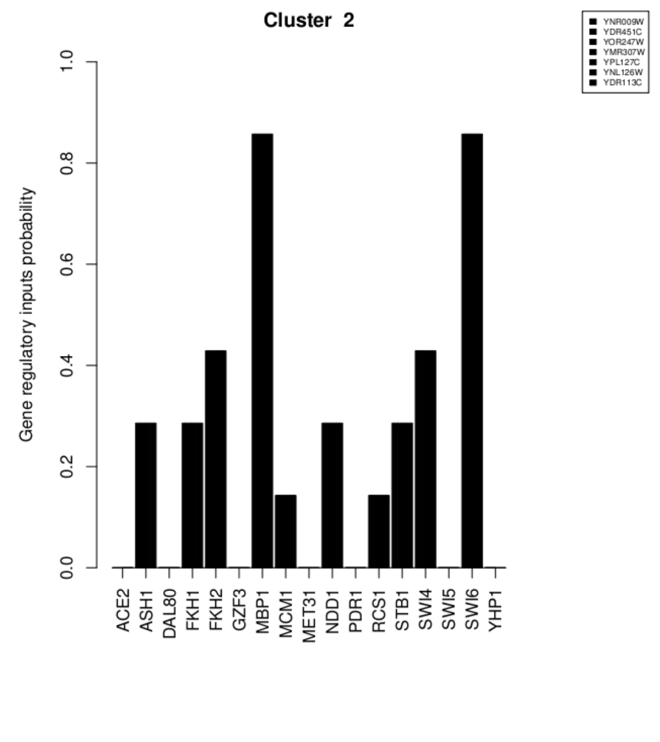

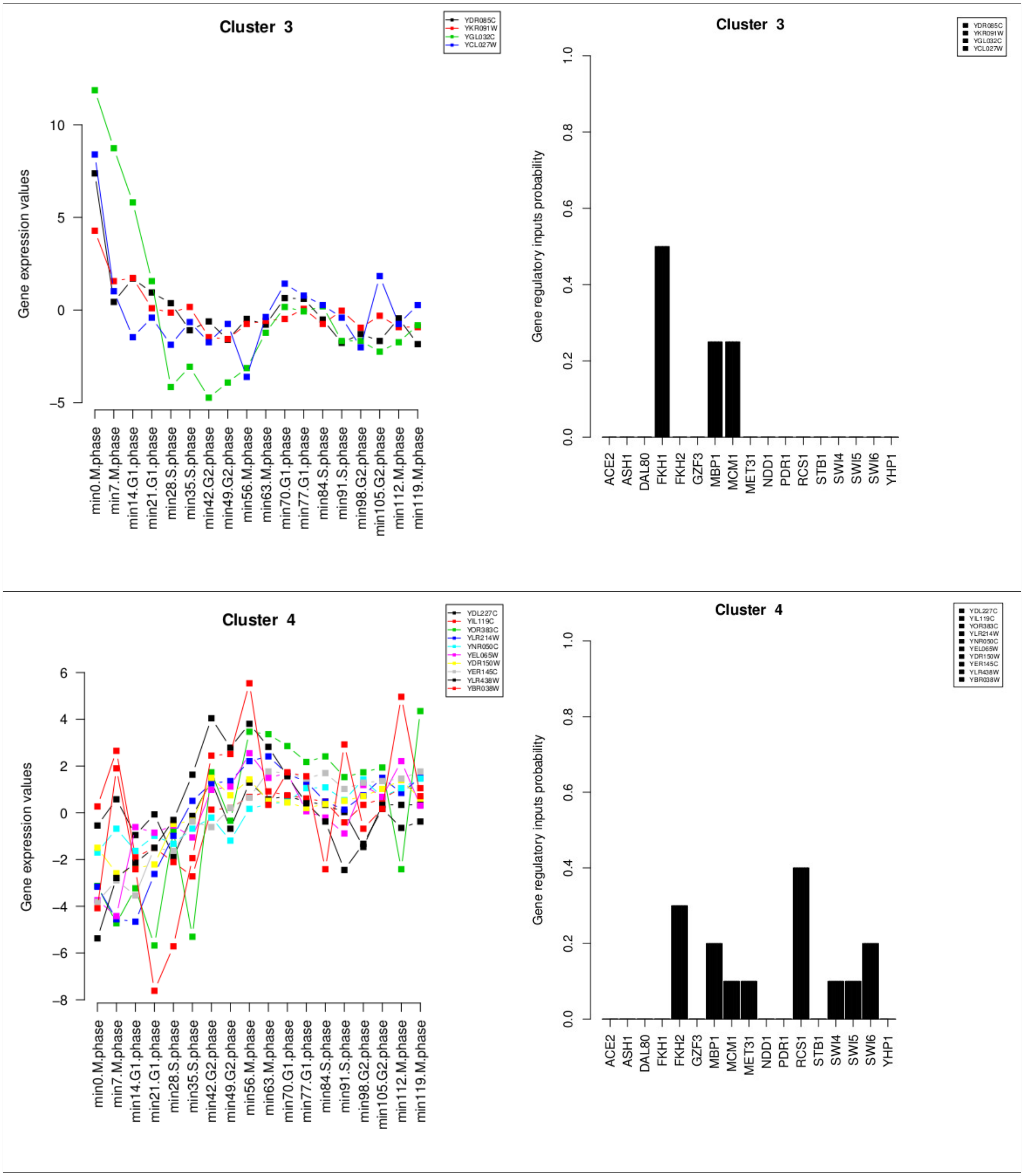

Cluster 3
YDR085C
YKR091W
YGL032C
YCL027W
Gene expression values
10
5
0
-5
min0.M.phase
min7.M.phase
min14.G1.phase
min21.G1.phase
min28.S.phase
min35.S.phase
min42.G2.phase
min49.G2.phase
min56.M.phase
min63.M.phase
min70.G1.phase
min77.G1.phase
min84.S.phase
min91.S.phase
min98.G2.phase
min105.G2.phase
min112.M.phase
min119.M.phase
Cluster 3
YDR085C
YKR091W
YGL032C
YCL027W
Gene regulatory inputs probability
1.0
0.8
0.6
0.4
0.2
0.0
ACE2
ASH1
DAL80
FKH1
FKH2
GZF3
MBP1
MCM1
MET31
NDD1
PDR1
RCS1
STB1
SWI4
SWI5
SWI6
YHP1
Cluster 4
YDL227C
YIL119C
YOR383C
YLR214W
YNR050C
YEL065W
YDR150W
YER145C
YLR438W
YBR038W
Gene expression values
6
4
2
0
-2
-4
-6
-8
min0.M.phase
min7.M.phase
min14.G1.phase
min21.G1.phase
min28.S.phase
min35.S.phase
min42.G2.phase
min49.G2.phase
min56.M.phase
min63.M.phase
min70.G1.phase
min77.G1.phase
min84.S.phase
min91.S.phase
min98.G2.phase
min105.G2.phase
min112.M.phase
min119.M.phase
Cluster 4
YDL227C
YIL119C
YOR383C
YLR214W
YNR050C
YEL065W
YDR150W
YER145C
YLR438W
YBR038W
Gene regulatory inputs probability
1.0
0.8
0.6
0.4
0.2
0.0
ACE2
ASH1
DAL80
FKH1
FKH2
GZF3
MBP1
MCM1
MET31
NDD1
PDR1
RCS1
STB1
SWI4
SWI5
SWI6
YHP1

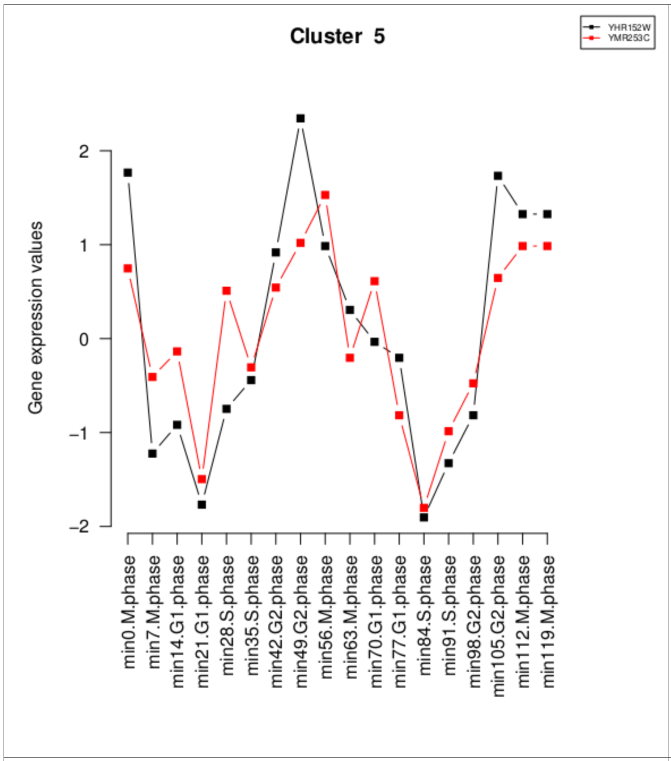
Cluster 5
YHR152W
YMR253C
Gene expression values
2
1
0
−1
−2
min0.M.phase
min7.M.phase
min14.G1.phase
min21.G1.phase
min28.S.phase
min35.S.phase
min42.G2.phase
min49.G2.phase
min56.M.phase
min63.M.phase
min70.G1.phase
min77.G1.phase
min84.S.phase
min91.S.phase
min98.G2.phase
min105.G2.phase
min112.M.phase
min119.M.phase
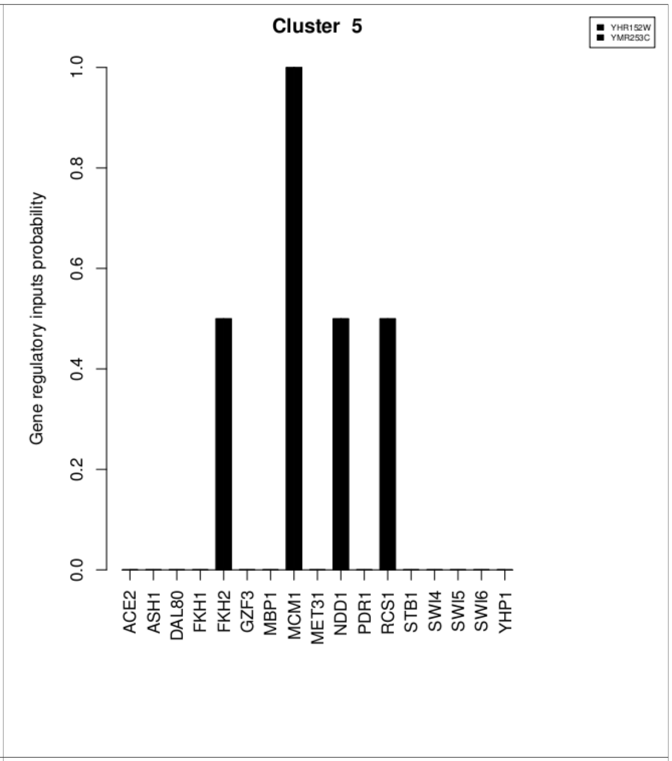
Cluster 5
YHR152W
YMR253C
Gene regulatory inputs probability
0.0
0.2
0.4
0.6
0.8
1.0
ACE2
ASH1
DAL80
FKH1
FKH2
GZF3
MBP1
MCM1
MET31
NDD1
PDR1
RCS1
STB1
SWI4
SWI5
SWI6
YHP1
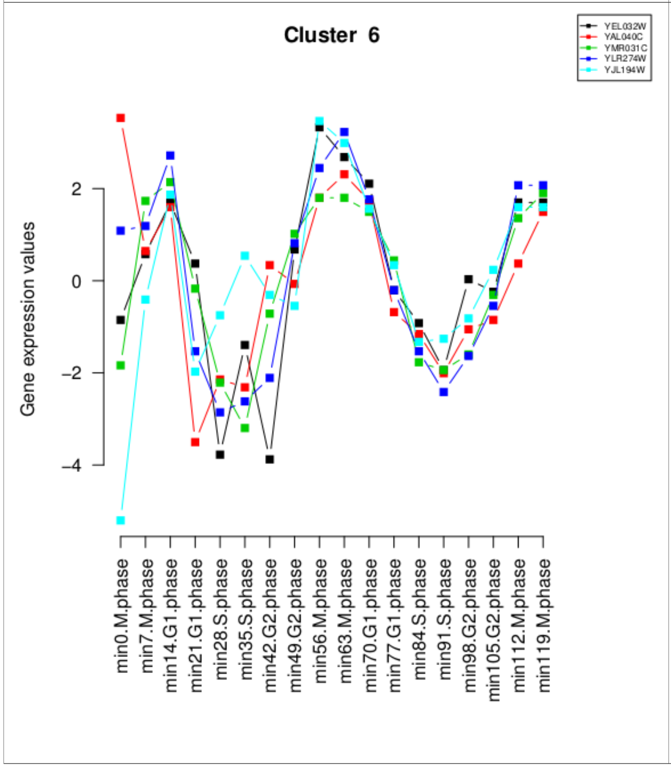
Cluster 6
YEL032W
YAL040C
YMR031C
YLR274W
YJL194W
Gene expression values
2
0
−2
−4
min0.M.phase
min7.M.phase
min14.G1.phase
min21.G1.phase
min28.S.phase
min35.S.phase
min42.G2.phase
min49.G2.phase
min56.M.phase
min63.M.phase
min70.G1.phase
min77.G1.phase
min84.S.phase
min91.S.phase
min98.G2.phase
min105.G2.phase
min112.M.phase
min119.M.phase
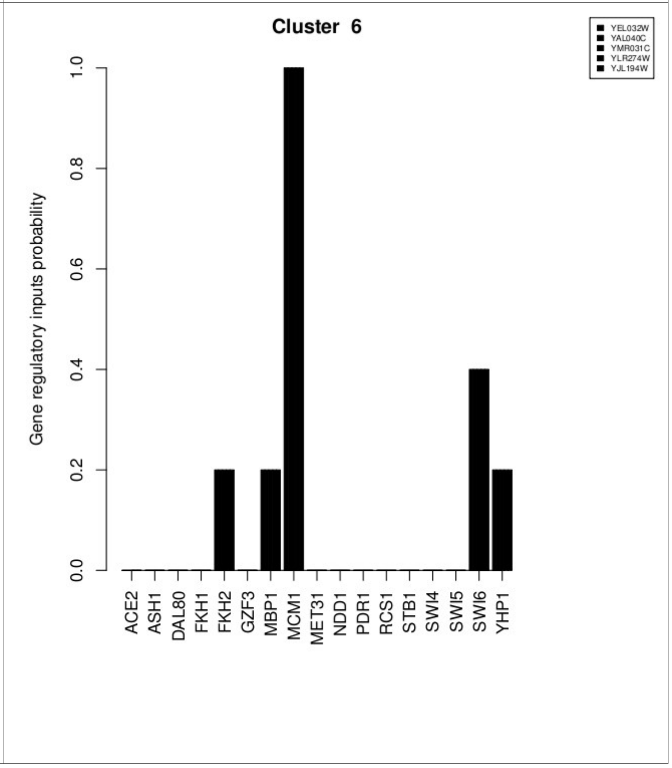
Cluster 6
YEL032W
YAL040C
YMR031C
YLR274W
YJL194W
Gene regulatory inputs probability
0.0
0.2
0.4
0.6
0.8
1.0
ACE2
ASH1
DAL80
FKH1
FKH2
GZF3
MBP1
MCM1
MET31
NDD1
PDR1
RCS1
STB1
SWI4
SWI5
SWI6
YHP1

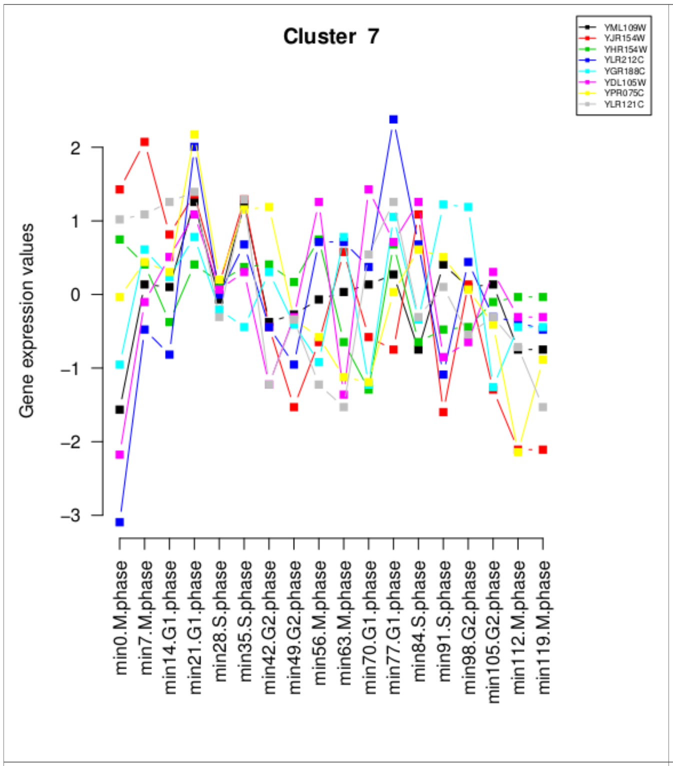

Cluster 7
YML109W
YJR154W
YHR154W
YLR212C
YGR188C
YDL105W
YPR075C
YLR121C
Gene expression values
2
1
0
−1
−2
−3
min0.M.phase
min7.M.phase
min14.G1.phase
min21.G1.phase
min28.S.phase
min35.S.phase
min42.G2.phase
min49.G2.phase
min56.M.phase
min63.M.phase
min70.G1.phase
min77.G1.phase
min84.S.phase
min91.S.phase
min98.G2.phase
min105.G2.phase
min112.M.phase
min119.M.phase

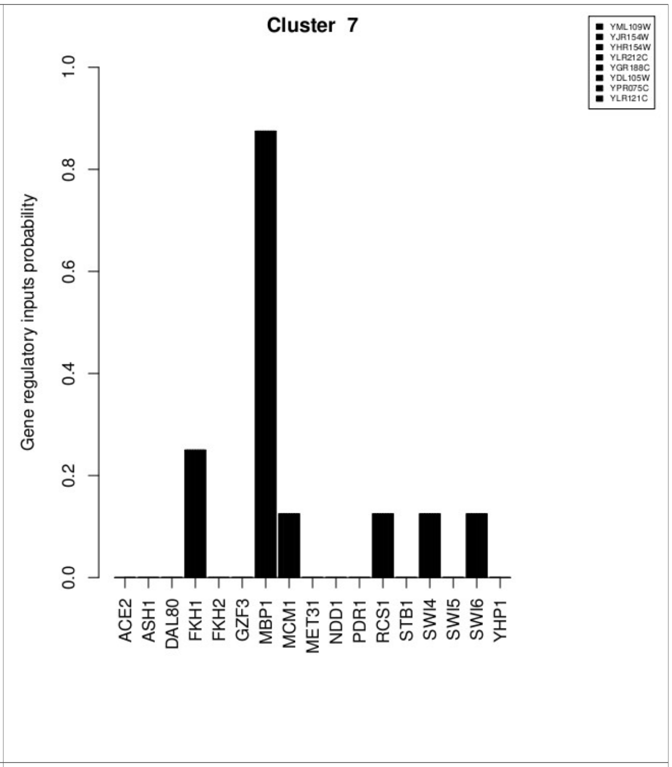

Cluster 7
YML109W
YJR154W
YHR154W
YLR212C
YGR188C
YDL105W
YPR075C
YLR121C
Gene regulatory inputs probability
1.0
0.8
0.6
0.4
0.2
0.0
ACE2
ASH1
DAL80
FKH1
FKH2
GZF3
MBP1
MCM1
MET31
NDD1
PDR1
RCS1
STB1
SWI4
SWI5
SWI6
YHP1

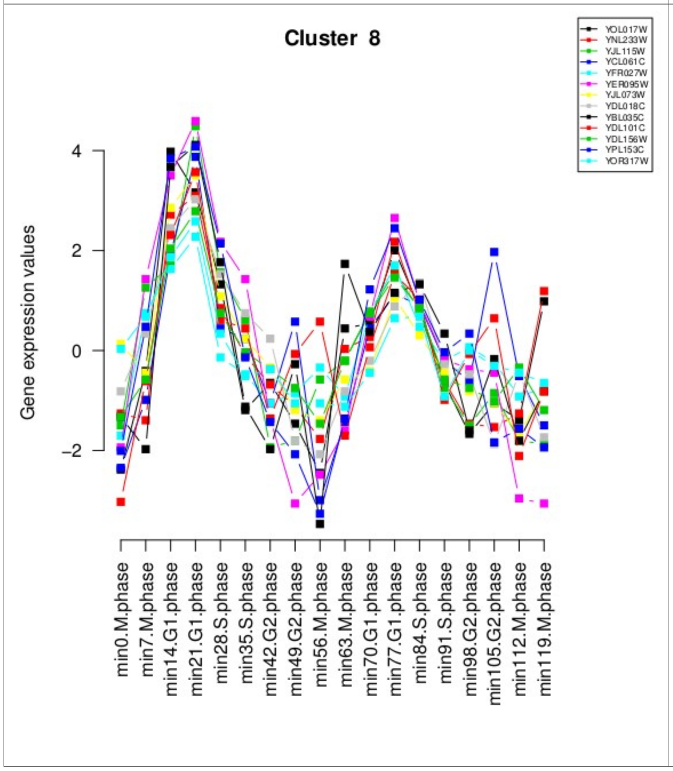

Cluster 8
YOL017W
YNL233W
YJL115W
YCL061C
YFR027W
YER095W
YJL073W
YDL018C
YBL035C
YDL101C
YDL156W
YPL153C
YOR317W
Gene expression values
4
2
0
−2
min0.M.phase
min7.M.phase
min14.G1.phase
min21.G1.phase
min28.S.phase
min35.S.phase
min42.G2.phase
min49.G2.phase
min56.M.phase
min63.M.phase
min70.G1.phase
min77.G1.phase
min84.S.phase
min91.S.phase
min98.G2.phase
min105.G2.phase
min112.M.phase
min119.M.phase

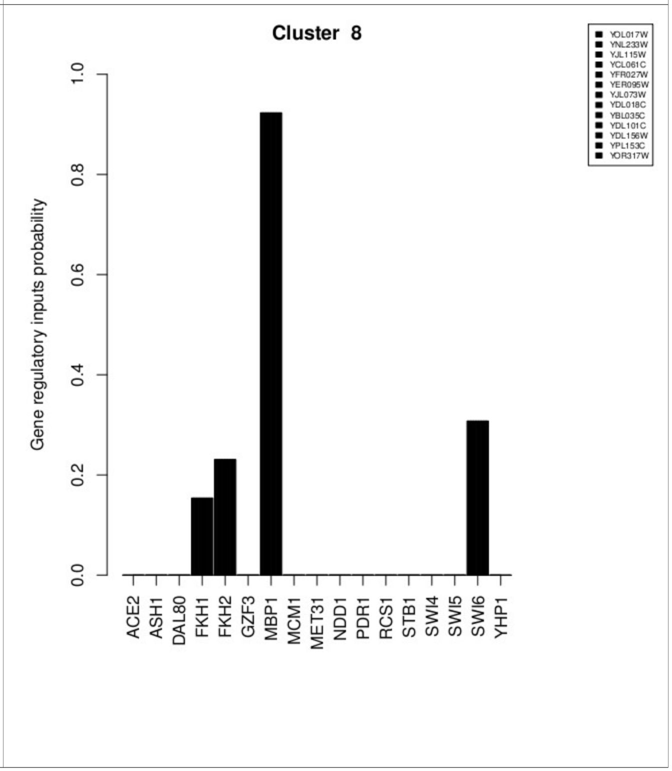

Cluster 8
YOL017W
YNL233W
YJL115W
YCL061C
YFR027W
YER095W
YJL073W
YDL018C
YBL035C
YDL101C
YDL156W
YPL153C
YOR317W
Gene regulatory inputs probability
1.0
0.8
0.6
0.4
0.2
0.0
ACE2
ASH1
DAL80
FKH1
FKH2
GZF3
MBP1
MCM1
MET31
NDD1
PDR1
RCS1
STB1
SWI4
SWI5
SWI6
YHP1

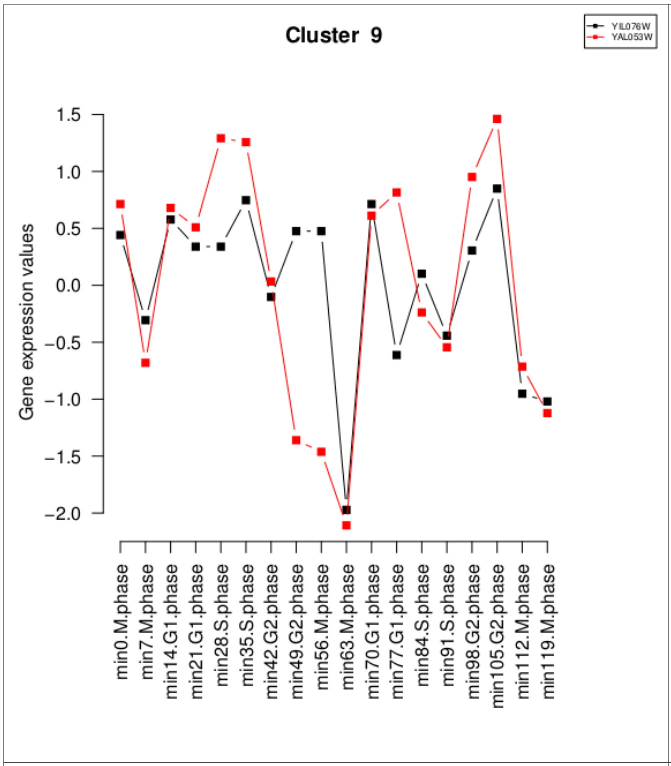
Cluster 9
YIL076W
YAL053W
Gene expression values
1.5
1.0
0.5
0.0
−0.5
−1.0
−1.5
−2.0
min0.M.phase
min7.M.phase
min14.G1.phase
min21.G1.phase
min28.S.phase
min35.S.phase
min42.G2.phase
min49.G2.phase
min56.M.phase
min63.M.phase
min70.G1.phase
min77.G1.phase
min84.S.phase
min91.S.phase
min98.G2.phase
min105.G2.phase
min112.M.phase
min119.M.phase

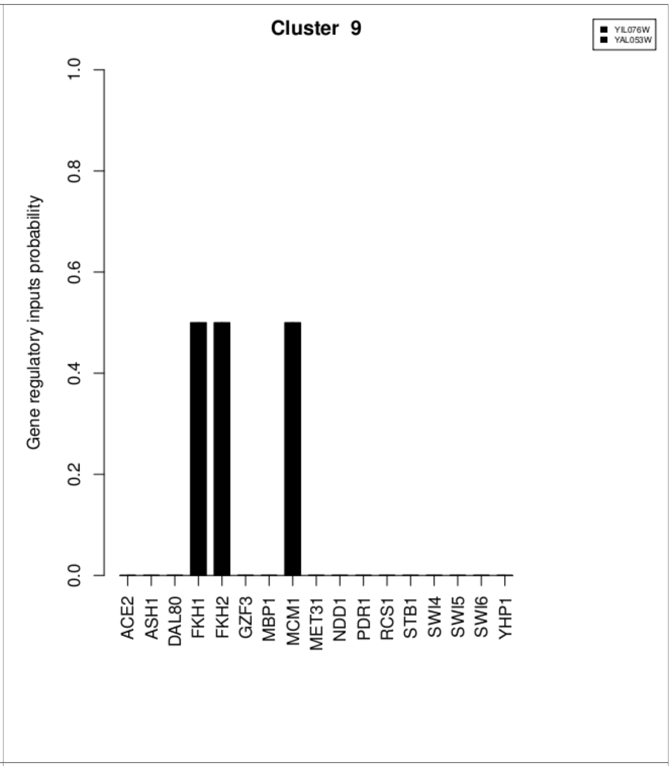
Cluster 9
YIL076W
YAL053W
Gene regulatory inputs probability
0.0
0.2
0.4
0.6
0.8
1.0
ACE2
ASH1
DAL80
FKH1
FKH2
GZF3
MBP1
MCM1
MET31
NDD1
PDR1
RCS1
STB1
SWI4
SWI5
SWI6
YHP1

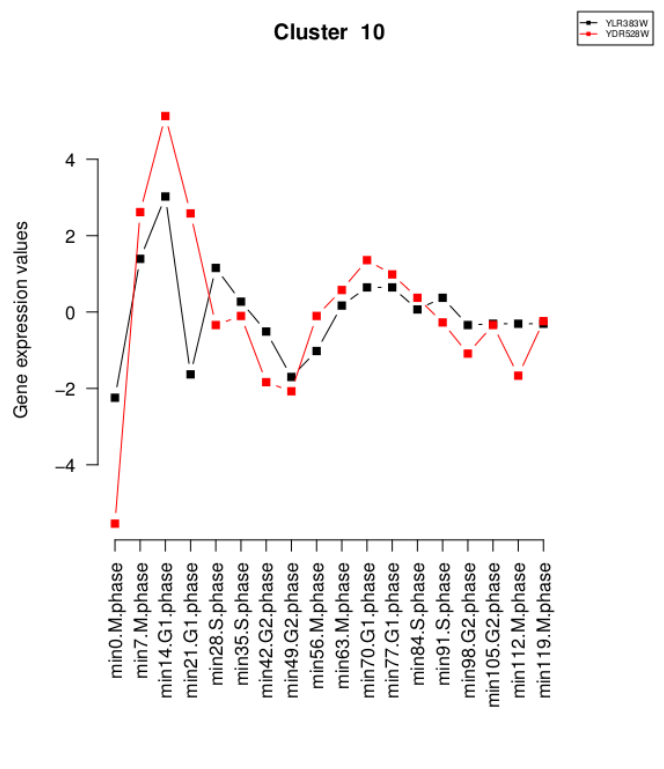
Cluster 10
YLR383W
YDR528W
Gene expression values
4
2
0
−2
−4
min0.M.phase
min7.M.phase
min14.G1.phase
min21.G1.phase
min28.S.phase
min35.S.phase
min42.G2.phase
min49.G2.phase
min56.M.phase
min63.M.phase
min70.G1.phase
min77.G1.phase
min84.S.phase
min91.S.phase
min98.G2.phase
min105.G2.phase
min112.M.phase
min119.M.phase

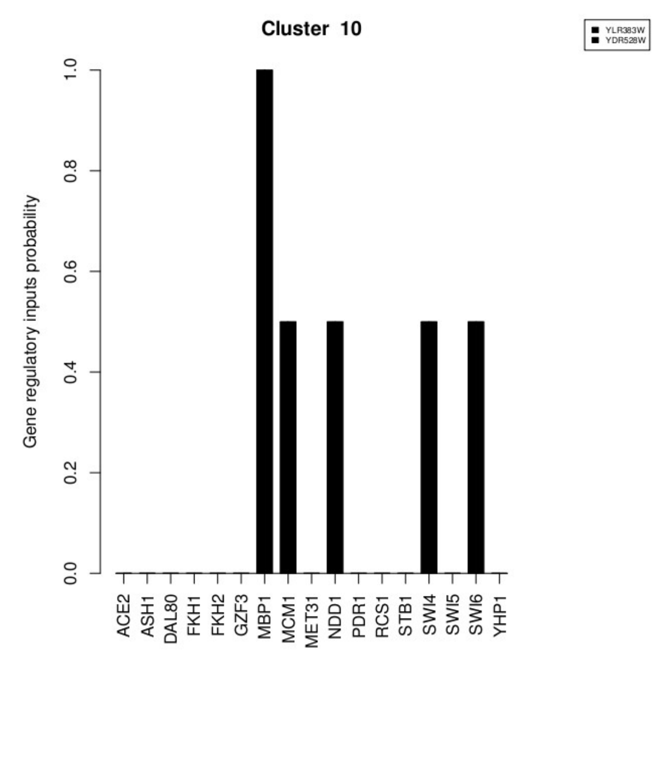
Cluster 10
YLR383W
YDR528W
Gene regulatory inputs probability
0.0
0.2
0.4
0.6
0.8
1.0
ACE2
ASH1
DAL80
FKH1
FKH2
GZF3
MBP1
MCM1
MET31
NDD1
PDR1
RCS1
STB1
SWI4
SWI5
SWI6
YHP1

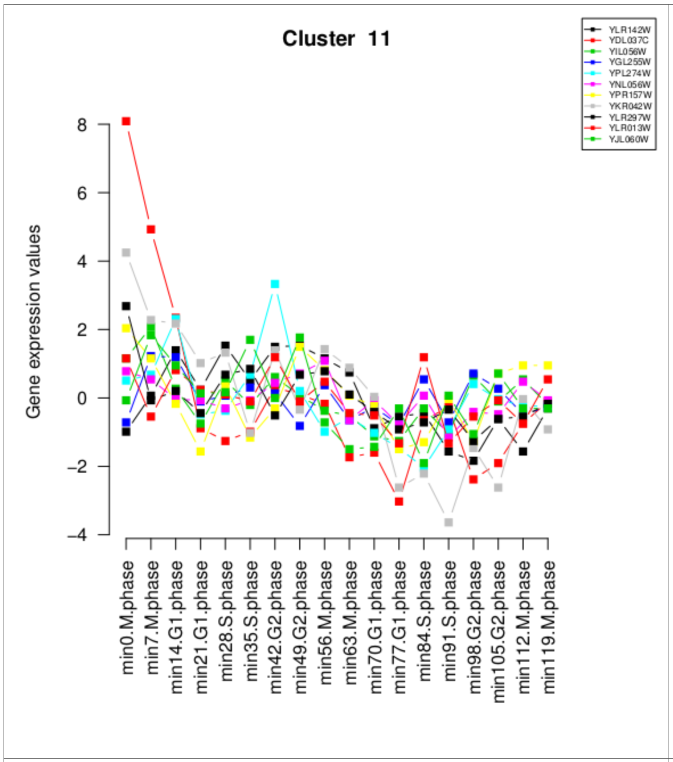
Cluster 11
Gene expression values
8
6
4
2
0
−2
−4
min0.M.phase
min7.M.phase
min14.G1.phase
min21.G1.phase
min28.S.phase
min35.S.phase
min42.G2.phase
min49.G2.phase
min56.M.phase
min63.M.phase
min70.G1.phase
min77.G1.phase
min84.S.phase
min91.S.phase
min98.G2.phase
min105.G2.phase
min112.M.phase
min119.M.phase
YLR142W
YDL037C
YIL056W
YGL255W
YPL274W
YNL056W
YPR157W
YKR042W
YLR297W
YLR013W
YJL060W

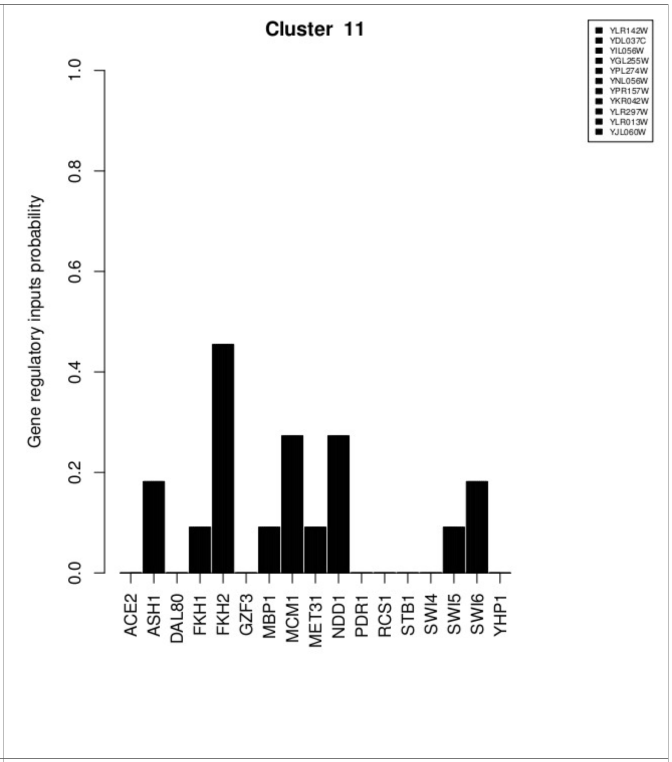
Cluster 11
Gene regulatory inputs probability
0.0
0.2
0.4
0.6
0.8
1.0
ACE2
ASH1
DAL80
FKH1
FKH2
GZF3
MBP1
MCM1
MET31
NDD1
PDR1
RCS1
STB1
SWI4
SWI5
SWI6
YHP1
YLR142W
YDL037C
YIL056W
YGL255W
YPL274W
YNL056W
YPR157W
YKR042W
YLR297W
YLR013W
YJL060W

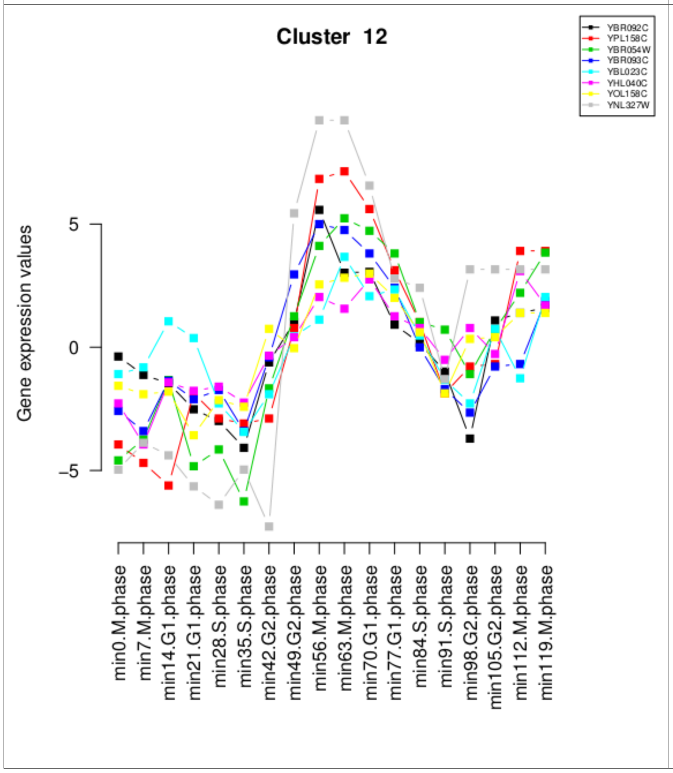
Cluster 12
Gene expression values
5
0
−5
min0.M.phase
min7.M.phase
min14.G1.phase
min21.G1.phase
min28.S.phase
min35.S.phase
min42.G2.phase
min49.G2.phase
min56.M.phase
min63.M.phase
min70.G1.phase
min77.G1.phase
min84.S.phase
min91.S.phase
min98.G2.phase
min105.G2.phase
min112.M.phase
min119.M.phase
YBR092C
YPL158C
YBR054W
YBR093C
YBL023C
YHL040C
YOL158C
YNL327W

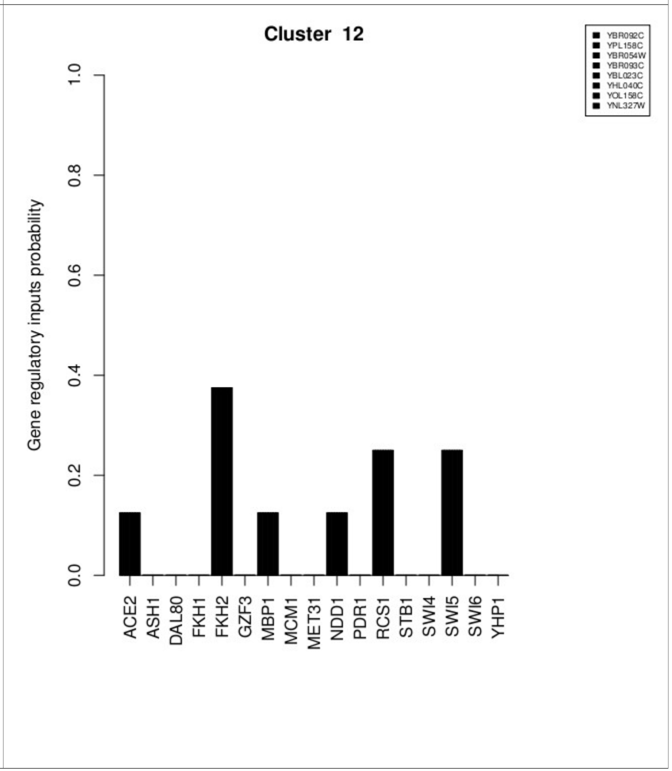
Cluster 12
Gene regulatory inputs probability
0.0
0.2
0.4
0.6
0.8
1.0
ACE2
ASH1
DAL80
FKH1
FKH2
GZF3
MBP1
MCM1
MET31
NDD1
PDR1
RCS1
STB1
SWI4
SWI5
SWI6
YHP1
YBR092C
YPL158C
YBR054W
YBR093C
YBL023C
YHL040C
YOL158C
YNL327W

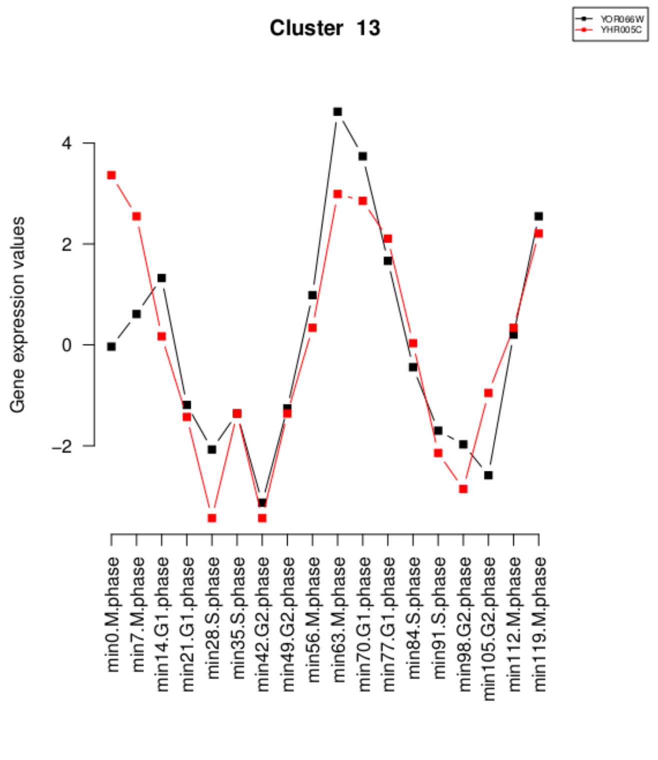
Cluster 13
YOR066W
YHR005C
Gene expression values
4
2
0
−2
min0.M.phase
min7.M.phase
min14.G1.phase
min21.G1.phase
min28.S.phase
min35.S.phase
min42.G2.phase
min49.G2.phase
min56.M.phase
min63.M.phase
min70.G1.phase
min77.G1.phase
min84.S.phase
min91.S.phase
min98.G2.phase
min105.G2.phase
min112.M.phase
min119.M.phase

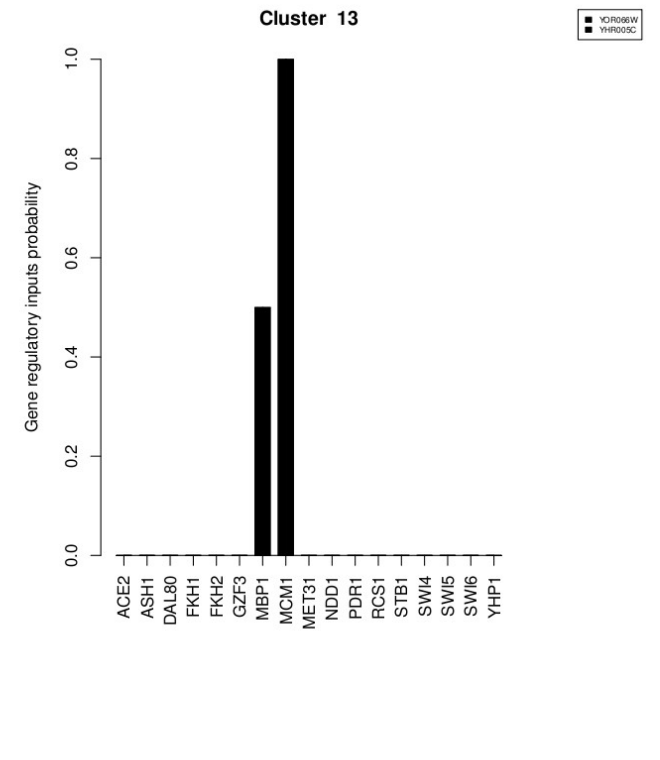
Cluster 13
YOR066W
YHR005C
Gene regulatory inputs probability
0.0
0.2
0.4
0.6
0.8
1.0
ACE2
ASH1
DAL80
FKH1
FKH2
GZF3
MBP1
MCM1
MET31
NDD1
PDR1
RCS1
STB1
SWI4
SWI5
SWI6
YHP1

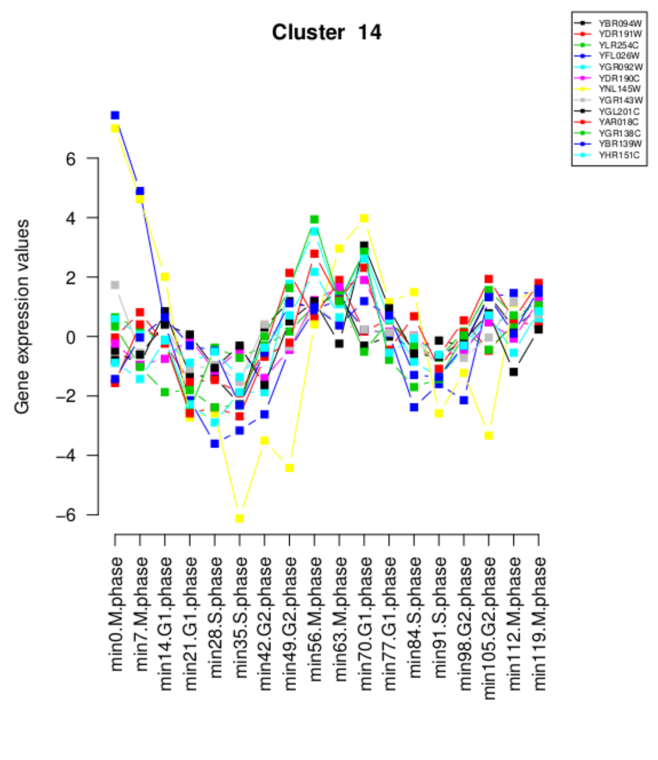
Cluster 14
YBR094W
YDR191W
YLR254C
YFL026W
YGR092W
YDR190C
YNL145W
YGR143W
YGL201C
YAR018C
YGR138C
YBR139W
YHR151C
Gene expression values
6
4
2
0
−2
−4
−6
min0.M.phase
min7.M.phase
min14.G1.phase
min21.G1.phase
min28.S.phase
min35.S.phase
min42.G2.phase
min49.G2.phase
min56.M.phase
min63.M.phase
min70.G1.phase
min77.G1.phase
min84.S.phase
min91.S.phase
min98.G2.phase
min105.G2.phase
min112.M.phase
min119.M.phase

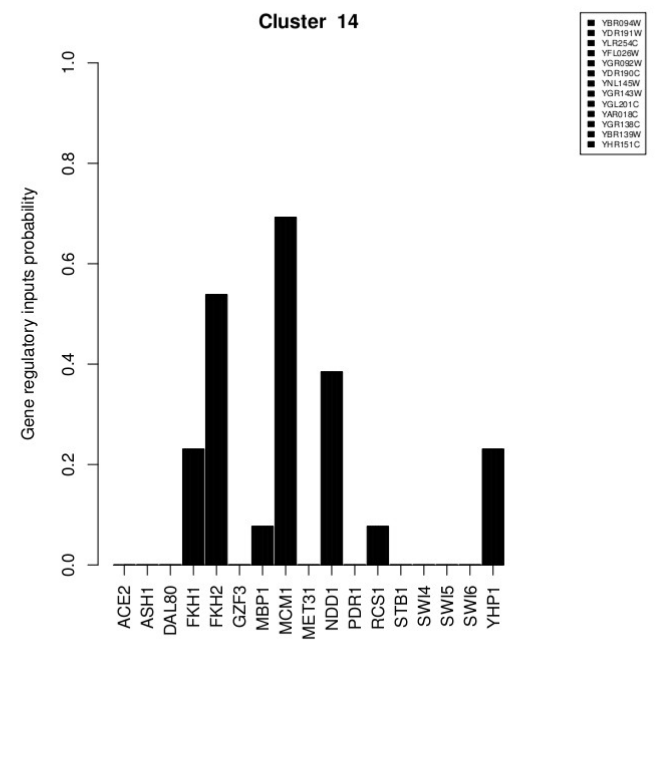
Cluster 14
YBR094W
YDR191W
YLR254C
YFL026W
YGR092W
YDR190C
YNL145W
YGR143W
YGL201C
YAR018C
YGR138C
YBR139W
YHR151C
Gene regulatory inputs probability
0.0
0.2
0.4
0.6
0.8
1.0
ACE2
ASH1
DAL80
FKH1
FKH2
GZF3
MBP1
MCM1
MET31
NDD1
PDR1
RCS1
STB1
SWI4
SWI5
SWI6
YHP1

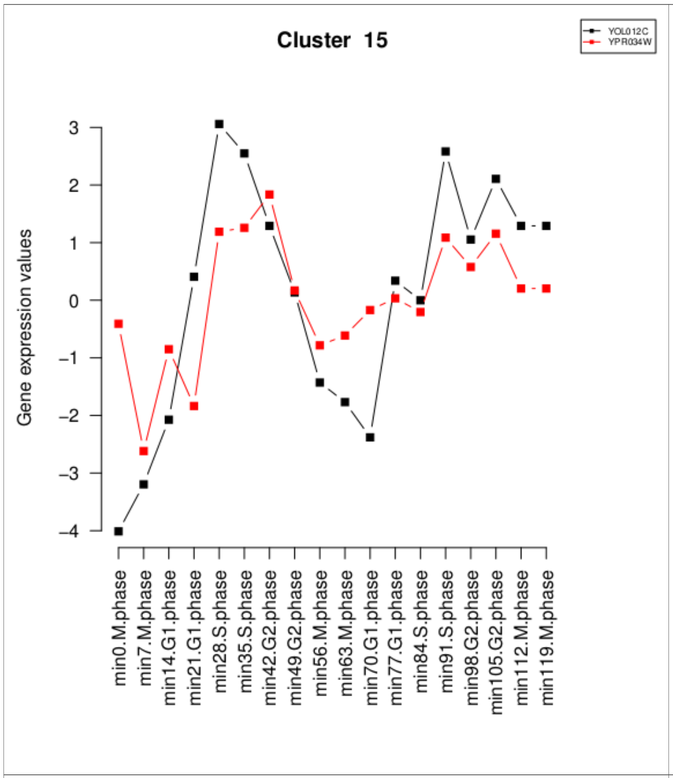
Cluster 15
YOL012C
YPR034W
Gene expression values
3
2
1
0
−1
−2
−3
−4
min0.M.phase
min7.M.phase
min14.G1.phase
min21.G1.phase
min28.S.phase
min35.S.phase
min42.G2.phase
min49.G2.phase
min56.M.phase
min63.M.phase
min70.G1.phase
min77.G1.phase
min84.S.phase
min91.S.phase
min98.G2.phase
min105.G2.phase
min112.M.phase
min119.M.phase

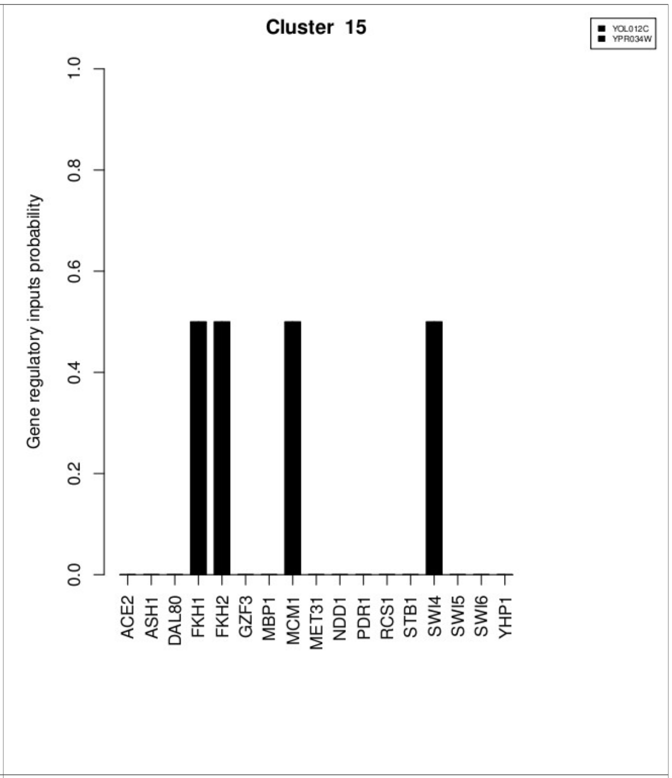
Cluster 15
YOL012C
YPR034W
Gene regulatory inputs probability
0.0
0.2
0.4
0.6
0.8
1.0
ACE2
ASH1
DAL80
FKH1
FKH2
GZF3
MBP1
MCM1
MET31
NDD1
PDR1
RCS1
STB1
SWI4
SWI5
SWI6
YHP1

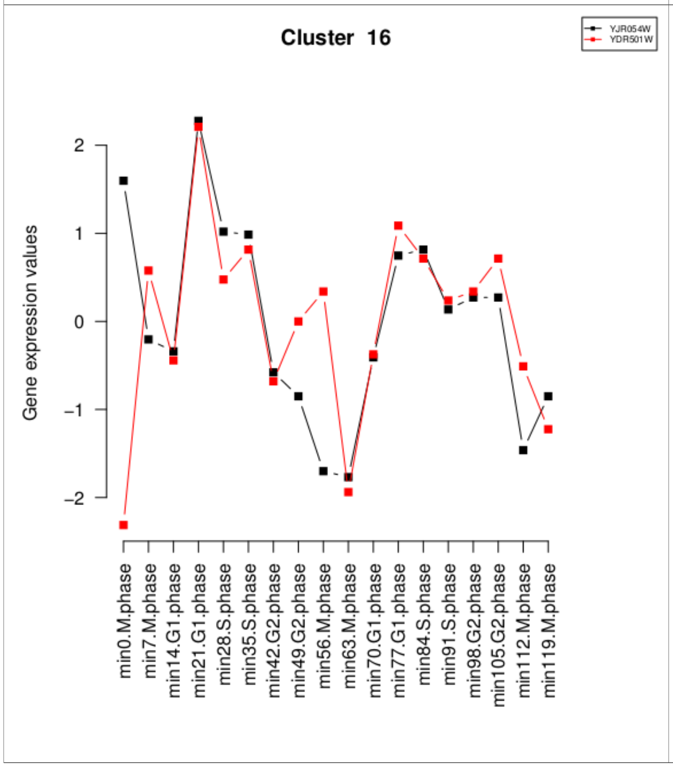
Cluster 16
YJR054W
YDR501W
Gene expression values
2
1
0
−1
−2
min0.M.phase
min7.M.phase
min14.G1.phase
min21.G1.phase
min28.S.phase
min35.S.phase
min42.G2.phase
min49.G2.phase
min56.M.phase
min63.M.phase
min70.G1.phase
min77.G1.phase
min84.S.phase
min91.S.phase
min98.G2.phase
min105.G2.phase
min112.M.phase
min119.M.phase

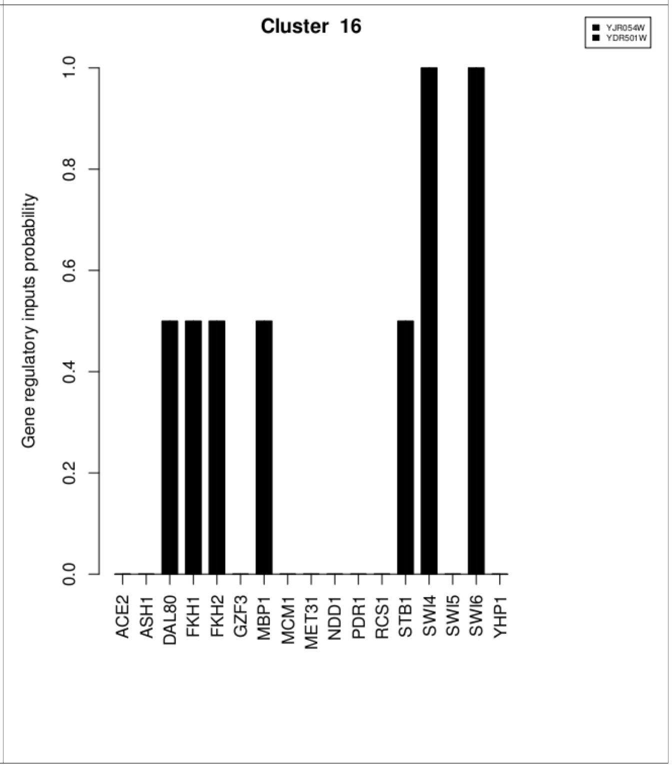
Cluster 16
YJR054W
YDR501W
Gene regulatory inputs probability
0.0
0.2
0.4
0.6
0.8
1.0
ACE2
ASH1
DAL80
FKH1
FKH2
GZF3
MBP1
MCM1
MET31
NDD1
PDR1
RCS1
STB1
SWI4
SWI5
SWI6
YHP1

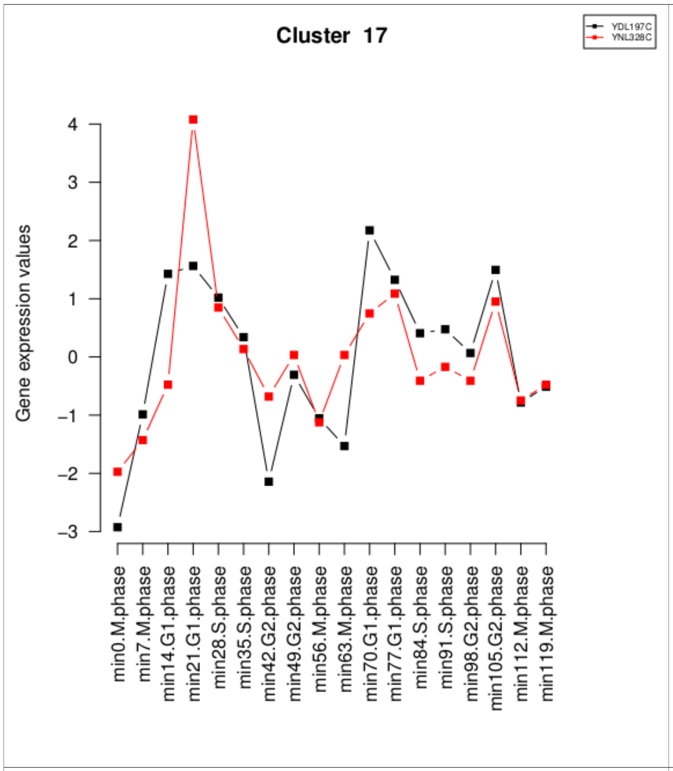
Cluster 17
YDL197C
YNL328C
Gene expression values
4
3
2
1
0
−1
−2
−3
min0.M.phase
min7.M.phase
min14.G1.phase
min21.G1.phase
min28.S.phase
min35.S.phase
min42.G2.phase
min49.G2.phase
min56.M.phase
min63.M.phase
min70.G1.phase
min77.G1.phase
min84.S.phase
min91.S.phase
min98.G2.phase
min105.G2.phase
min112.M.phase
min119.M.phase

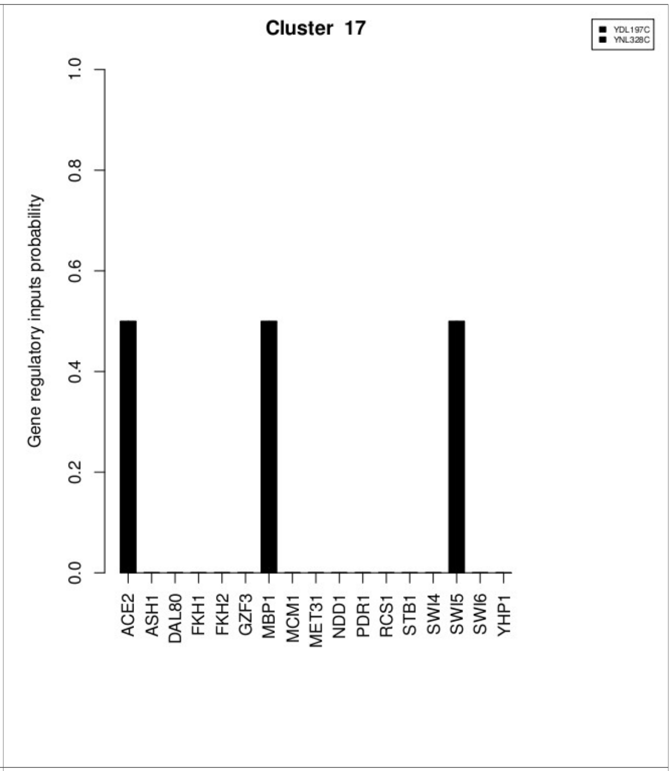
Cluster 17
YDL197C
YNL328C
Gene regulatory inputs probability
1.0
0.8
0.6
0.4
0.2
0.0
ACE2
ASH1
DAL80
FKH1
FKH2
GZF3
MBP1
MCM1
MET31
NDD1
PDR1
RCS1
STB1
SWI4
SWI5
SWI6
YHP1

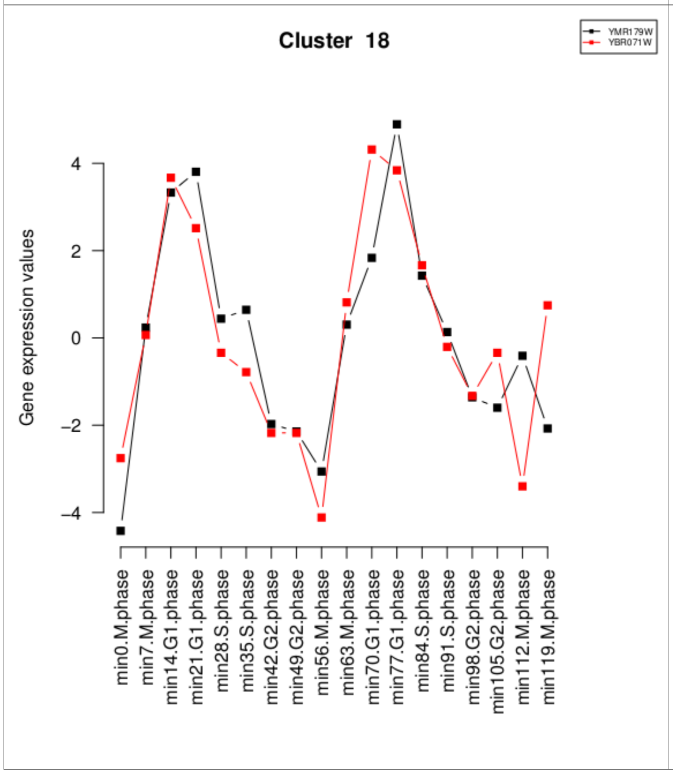
Cluster 18
YMR179W
YBR071W
Gene expression values
4
2
0
−2
−4
min0.M.phase
min7.M.phase
min14.G1.phase
min21.G1.phase
min28.S.phase
min35.S.phase
min42.G2.phase
min49.G2.phase
min56.M.phase
min63.M.phase
min70.G1.phase
min77.G1.phase
min84.S.phase
min91.S.phase
min98.G2.phase
min105.G2.phase
min112.M.phase
min119.M.phase

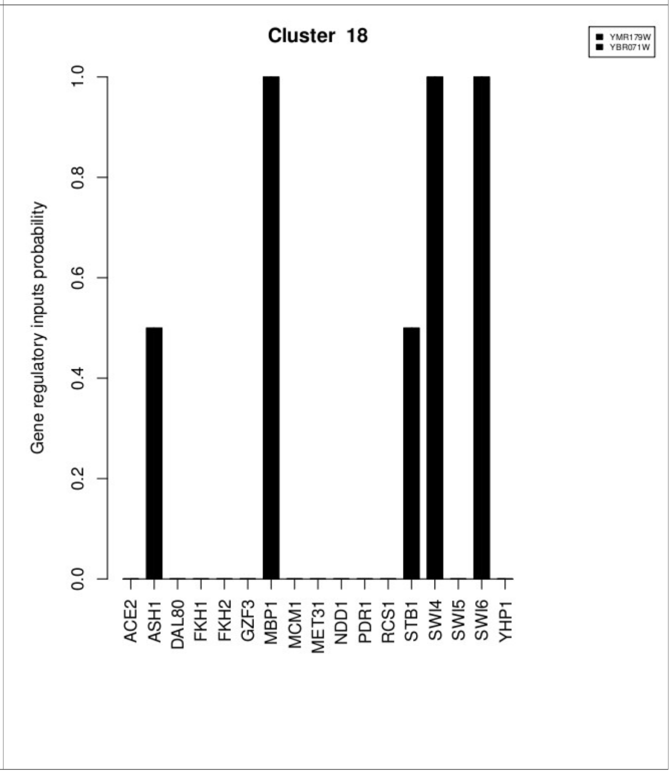
Cluster 18
YMR179W
YBR071W
Gene regulatory inputs probability
1.0
0.8
0.6
0.4
0.2
0.0
ACE2
ASH1
DAL80
FKH1
FKH2
GZF3
MBP1
MCM1
MET31
NDD1
PDR1
RCS1
STB1
SWI4
SWI5
SWI6
YHP1

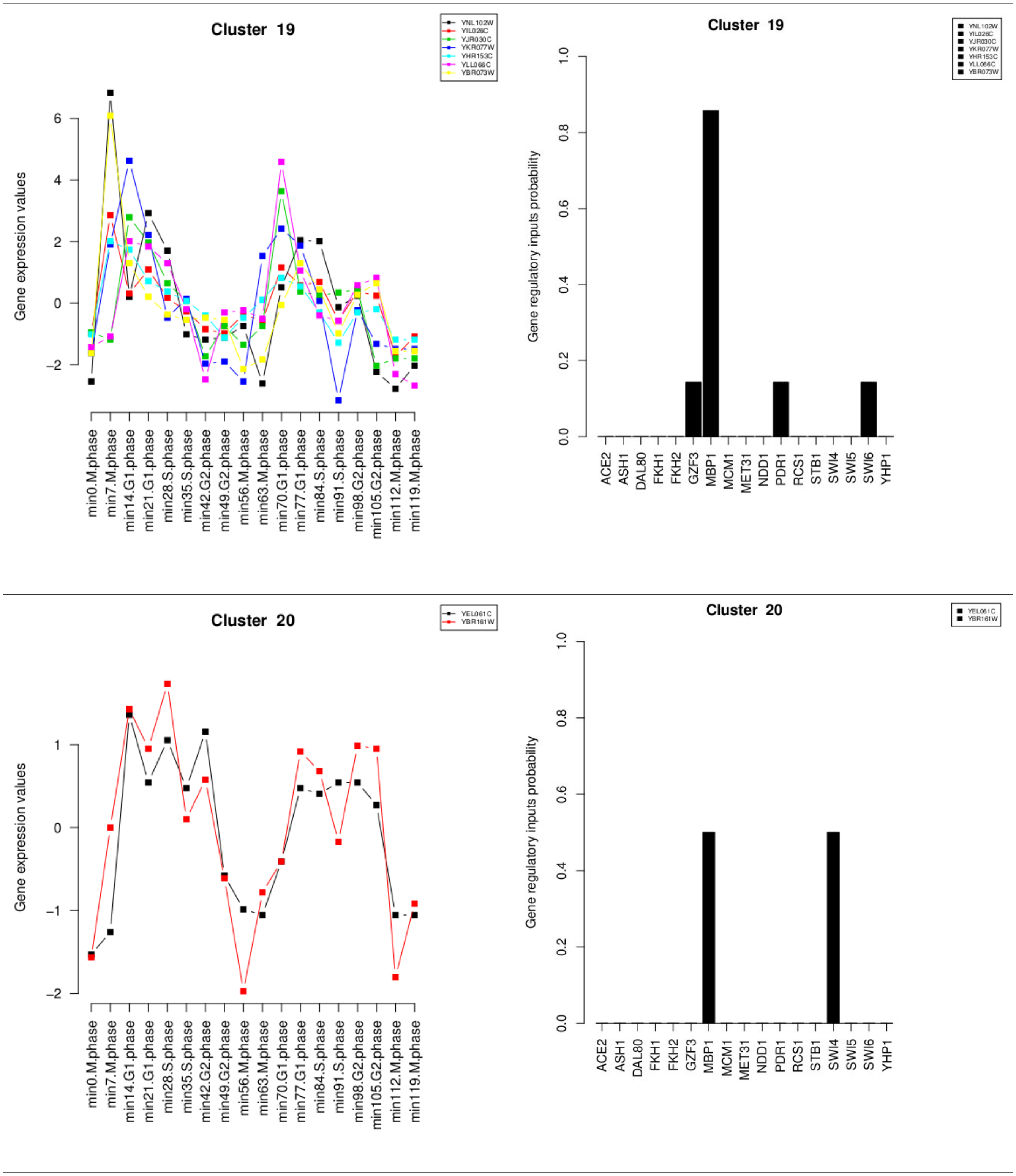
Cluster 19
YNL102W
YIL026C
YJR030C
YKR077W
YHR153C
YLL066C
YBR073W
Gene expression values
min0.M.phase
min7.M.phase
min14.G1.phase
min21.G1.phase
min28.S.phase
min35.S.phase
min42.G2.phase
min49.G2.phase
min56.M.phase
min63.M.phase
min70.G1.phase
min77.G1.phase
min84.S.phase
min91.S.phase
min98.G2.phase
min105.G2.phase
min112.M.phase
min119.M.phase
Cluster 19
Gene regulatory inputs probability
ACE2
ASH1
DAL80
FKH1
FKH2
GZF3
MBP1
MCM1
MET31
NDD1
PDR1
RCS1
STB1
SWI4
SWI5
SWI6
YHP1
Cluster 20
YEL061C
YBR161W
Gene expression values
Cluster 20
Gene regulatory inputs probability

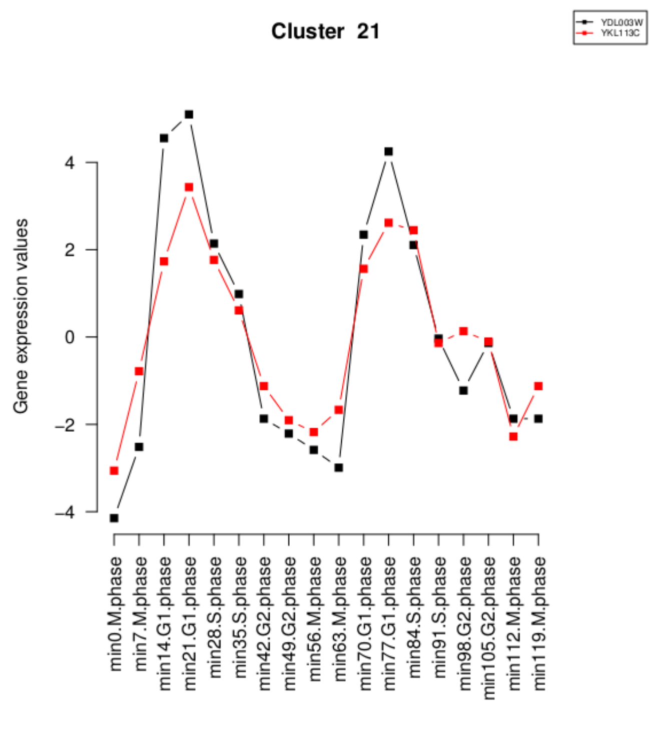
Cluster 21
YDL003W
YKL113C
Gene expression values
4
2
0
−2
−4
min0.M.phase
min7.M.phase
min14.G1.phase
min21.G1.phase
min28.S.phase
min35.S.phase
min42.G2.phase
min49.G2.phase
min56.M.phase
min63.M.phase
min70.G1.phase
min77.G1.phase
min84.S.phase
min91.S.phase
min98.G2.phase
min105.G2.phase
min112.M.phase
min119.M.phase

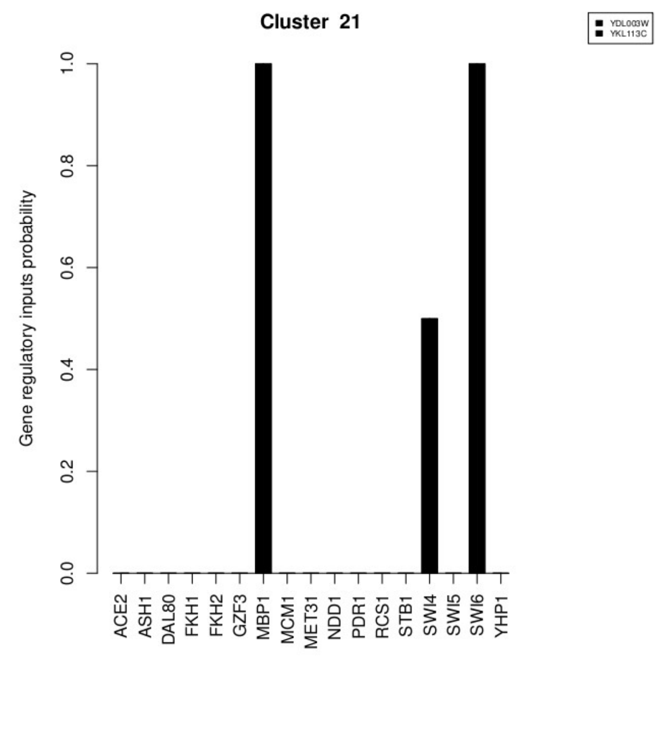
Cluster 21
YDL003W
YKL113C
Gene regulatory inputs probability
1.0
0.8
0.6
0.4
0.2
0.0
ACE2
ASH1
DAL80
FKH1
FKH2
GZF3
MBP1
MCM1
MET31
NDD1
PDR1
RCS1
STB1
SWI4
SWI5
SWI6
YHP1

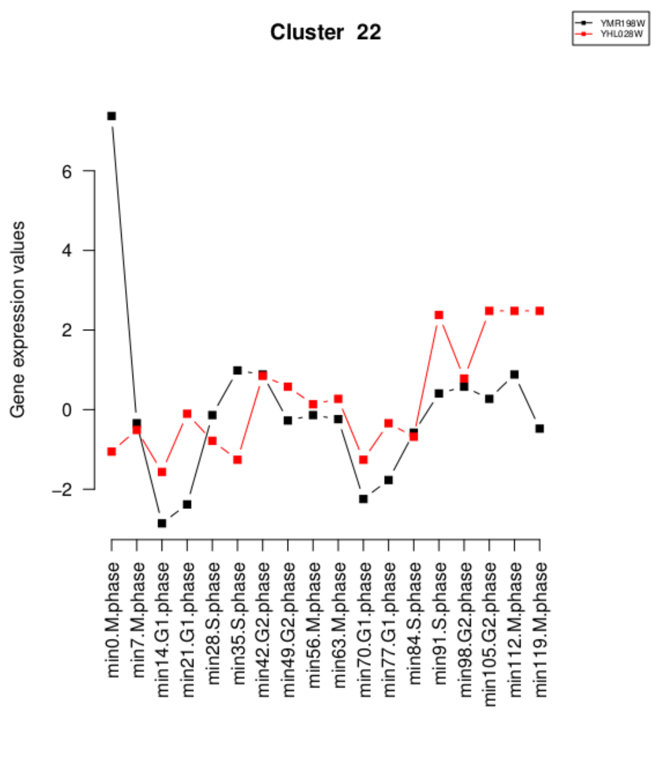
Cluster 22
YMR198W
YHL028W
Gene expression values
6
4
2
0
−2
min0.M.phase
min7.M.phase
min14.G1.phase
min21.G1.phase
min28.S.phase
min35.S.phase
min42.G2.phase
min49.G2.phase
min56.M.phase
min63.M.phase
min70.G1.phase
min77.G1.phase
min84.S.phase
min91.S.phase
min98.G2.phase
min105.G2.phase
min112.M.phase
min119.M.phase

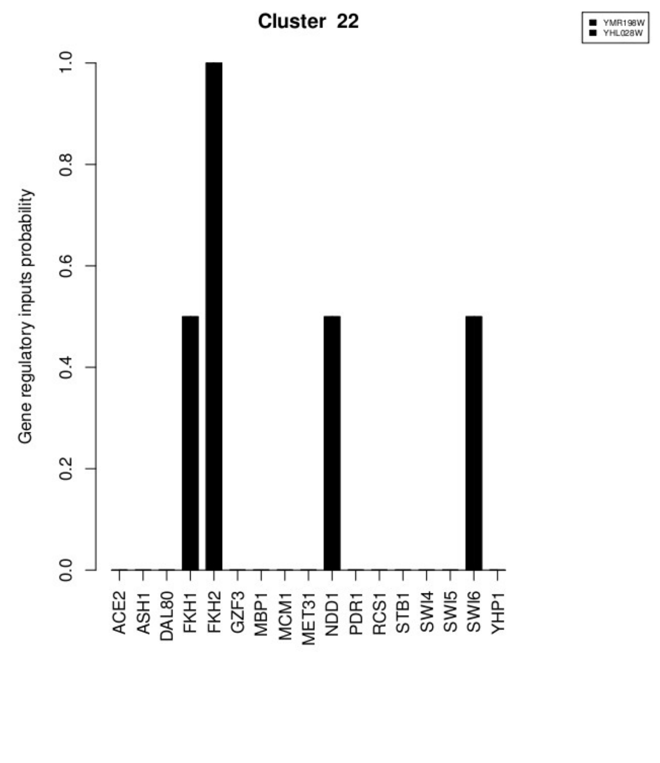
Cluster 22
YMR198W
YHL028W
Gene regulatory inputs probability
1.0
0.8
0.6
0.4
0.2
0.0
ACE2
ASH1
DAL80
FKH1
FKH2
GZF3
MBP1
MCM1
MET31
NDD1
PDR1
RCS1
STB1
SWI4
SWI5
SWI6
YHP1

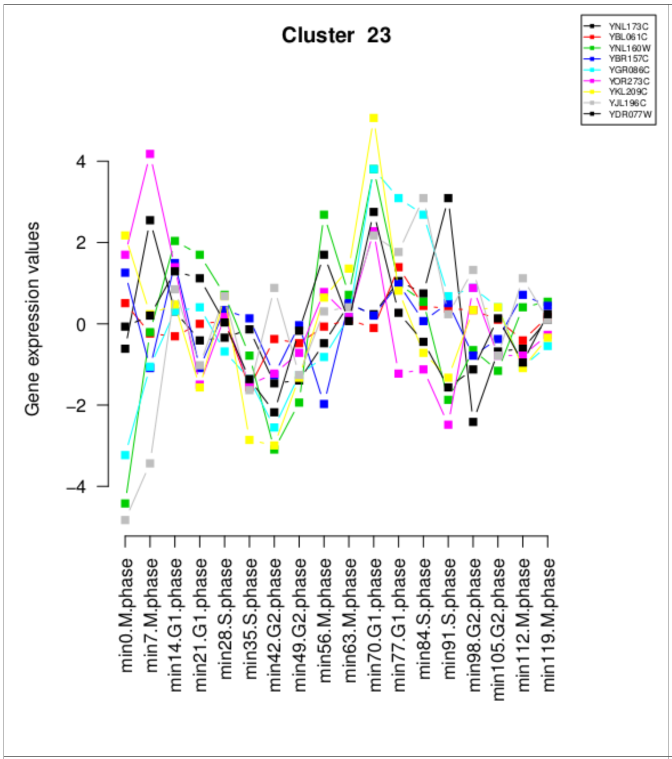
Cluster 23
YNL173C
YBL061C
YNL160W
YBR157C
YGR086C
YOR273C
YKL209C
YJL196C
YDR077W
Gene expression values
4
2
0
-2
-4
min0.M.phase
min7.M.phase
min14.G1.phase
min21.G1.phase
min28.S.phase
min35.S.phase
min42.G2.phase
min49.G2.phase
min56.M.phase
min63.M.phase
min70.G1.phase
min77.G1.phase
min84.S.phase
min91.S.phase
min98.G2.phase
min105.G2.phase
min112.M.phase
min119.M.phase

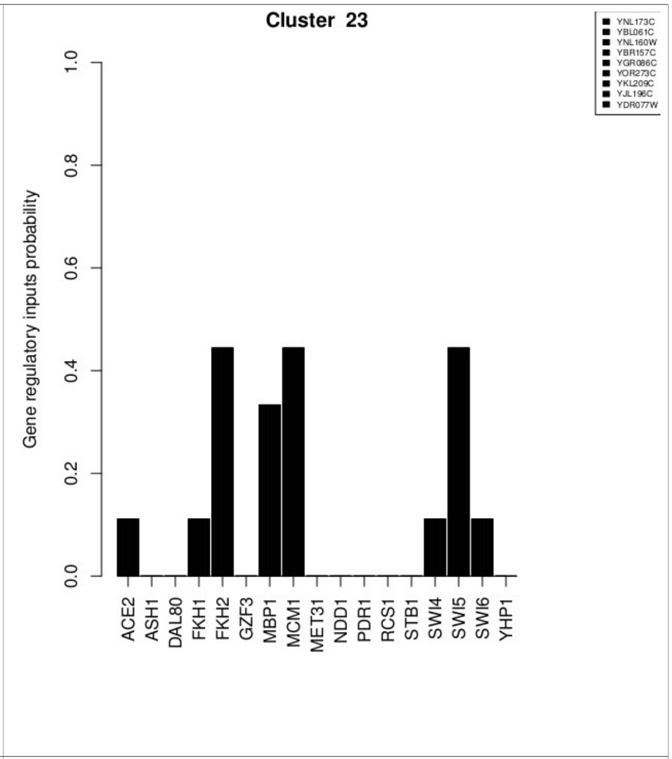
Cluster 23
YNL173C
YBL061C
YNL160W
YBR157C
YGR086C
YOR273C
YKL209C
YJL196C
YDR077W
Gene regulatory inputs probability
0.0
0.2
0.4
0.6
0.8
1.0
ACE2
ASH1
DAL80
FKH1
FKH2
GZF3
MBP1
MCM1
MET31
NDD1
PDR1
RCS1
STB1
SWI4
SWI5
SWI6
YHP1

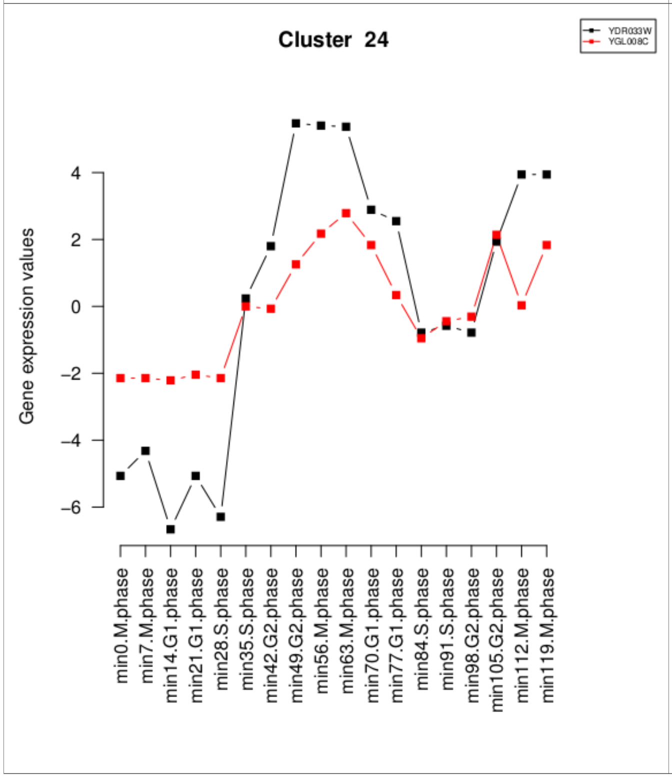
Cluster 24
YDR033W
YGL008C
Gene expression values
4
2
0
-2
-4
-6
min0.M.phase
min7.M.phase
min14.G1.phase
min21.G1.phase
min28.S.phase
min35.S.phase
min42.G2.phase
min49.G2.phase
min56.M.phase
min63.M.phase
min70.G1.phase
min77.G1.phase
min84.S.phase
min91.S.phase
min98.G2.phase
min105.G2.phase
min112.M.phase
min119.M.phase

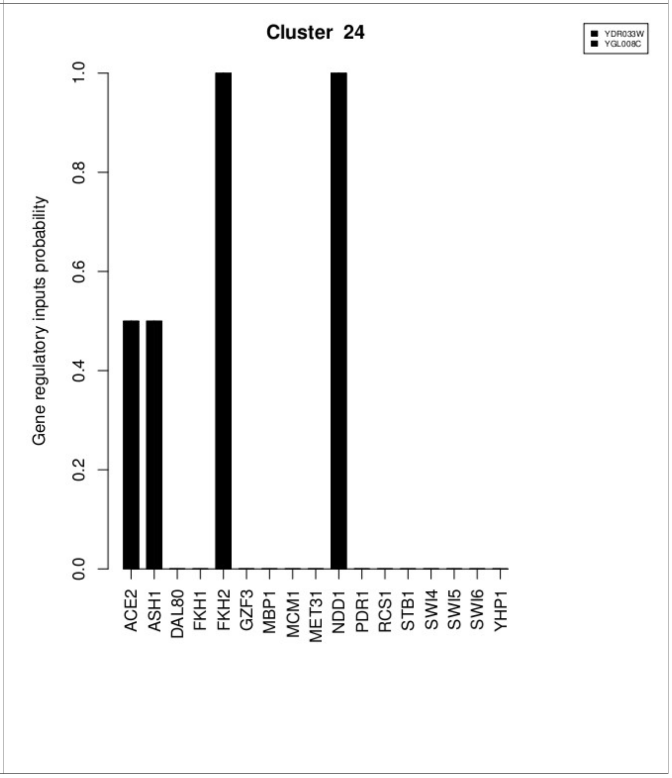
Cluster 24
YDR033W
YGL008C
Gene regulatory inputs probability
0.0
0.2
0.4
0.6
0.8
1.0
ACE2
ASH1
DAL80
FKH1
FKH2
GZF3
MBP1
MCM1
MET31
NDD1
PDR1
RCS1
STB1
SWI4
SWI5
SWI6
YHP1

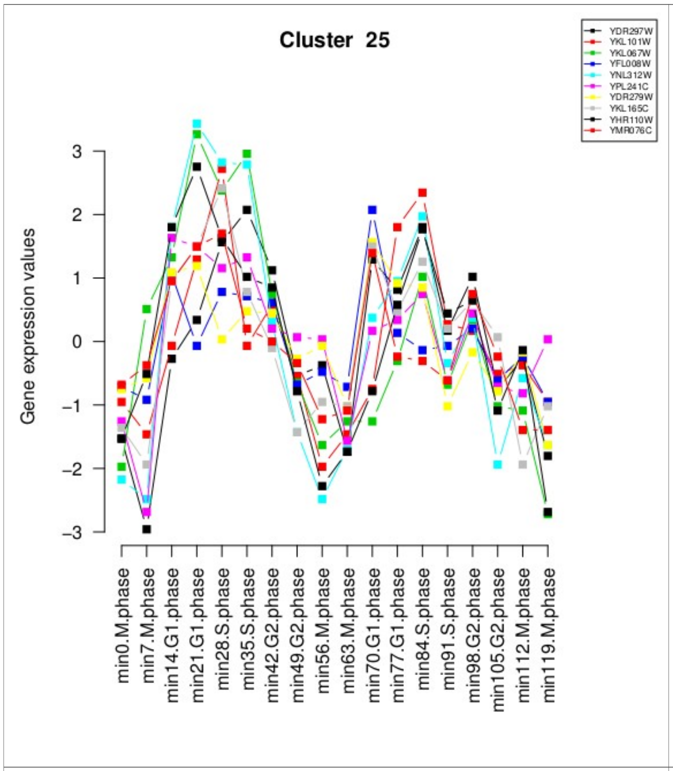
Cluster 25
Gene expression values
3
2
1
0
−1
−2
−3
min0.M.phase
min7.M.phase
min14.G1.phase
min21.G1.phase
min28.S.phase
min35.S.phase
min42.G2.phase
min49.G2.phase
min56.M.phase
min63.M.phase
min70.G1.phase
min77.G1.phase
min84.S.phase
min91.S.phase
min98.G2.phase
min105.G2.phase
min112.M.phase
min119.M.phase
YDR297W
YKL101W
YKL067W
YFL008W
YNL312W
YPL241C
YDR279W
YKL165C
YHR110W
YMR076C

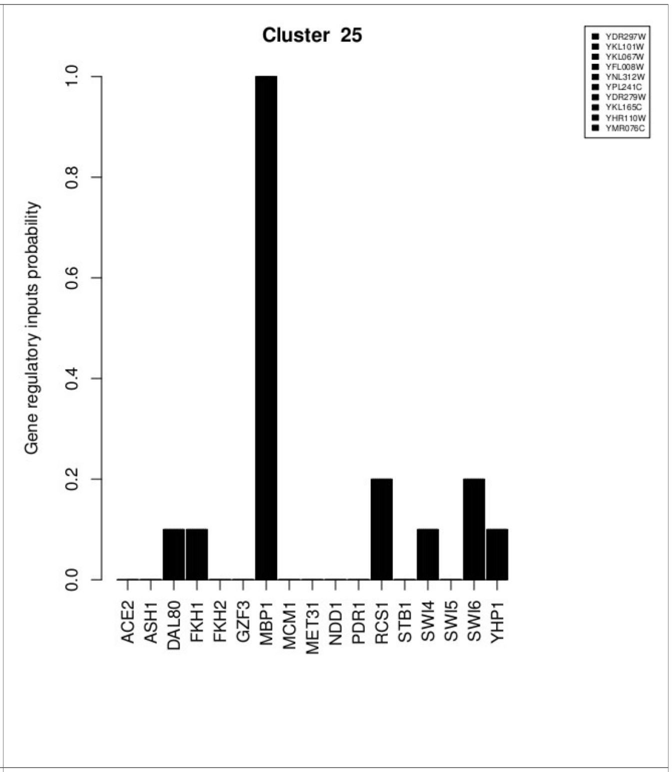
Cluster 25
Gene regulatory inputs probability
1.0
0.8
0.6
0.4
0.2
0.0
ACE2
ASH1
DAL80
FKH1
FKH2
GZF3
MBP1
MCM1
MET31
NDD1
PDR1
RCS1
STB1
SWI4
SWI5
SWI6
YHP1
YDR297W
YKL101W
YKL067W
YFL008W
YNL312W
YPL241C
YDR279W
YKL165C
YHR110W
YMR076C

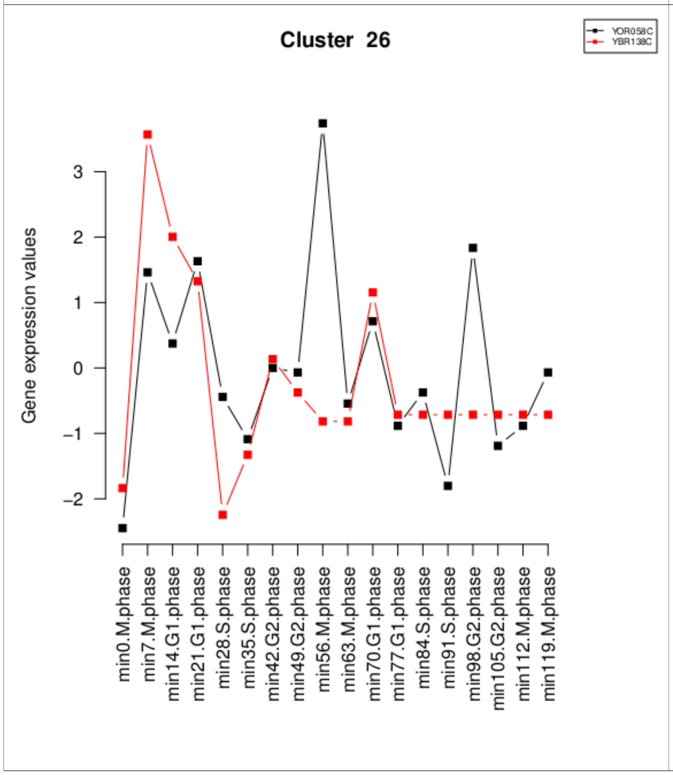
Cluster 26
Gene expression values
3
2
1
0
−1
−2
min0.M.phase
min7.M.phase
min14.G1.phase
min21.G1.phase
min28.S.phase
min35.S.phase
min42.G2.phase
min49.G2.phase
min56.M.phase
min63.M.phase
min70.G1.phase
min77.G1.phase
min84.S.phase
min91.S.phase
min98.G2.phase
min105.G2.phase
min112.M.phase
min119.M.phase
YOR058C
YBR138C

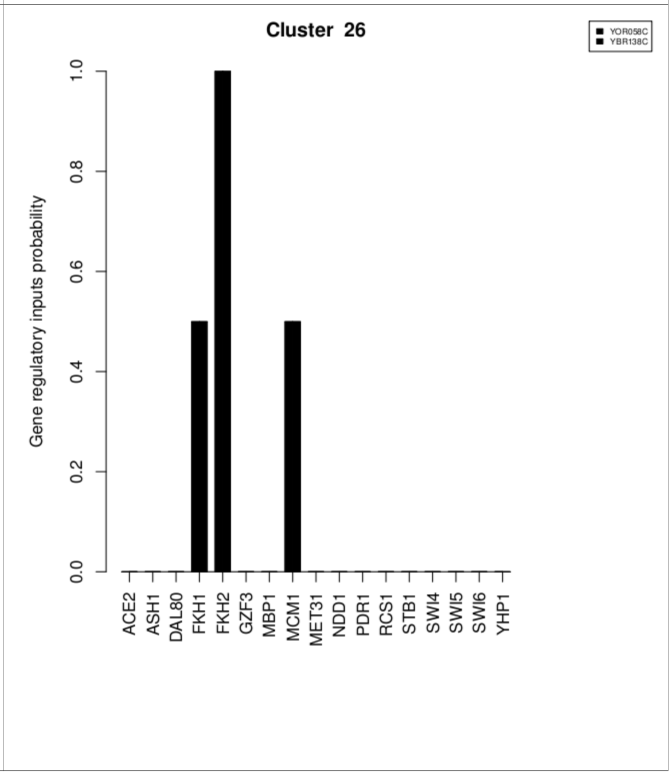
Cluster 26
Gene regulatory inputs probability
1.0
0.8
0.6
0.4
0.2
0.0
ACE2
ASH1
DAL80
FKH1
FKH2
GZF3
MBP1
MCM1
MET31
NDD1
PDR1
RCS1
STB1
SWI4
SWI5
SWI6
YHP1
YOR058C
YBR138C

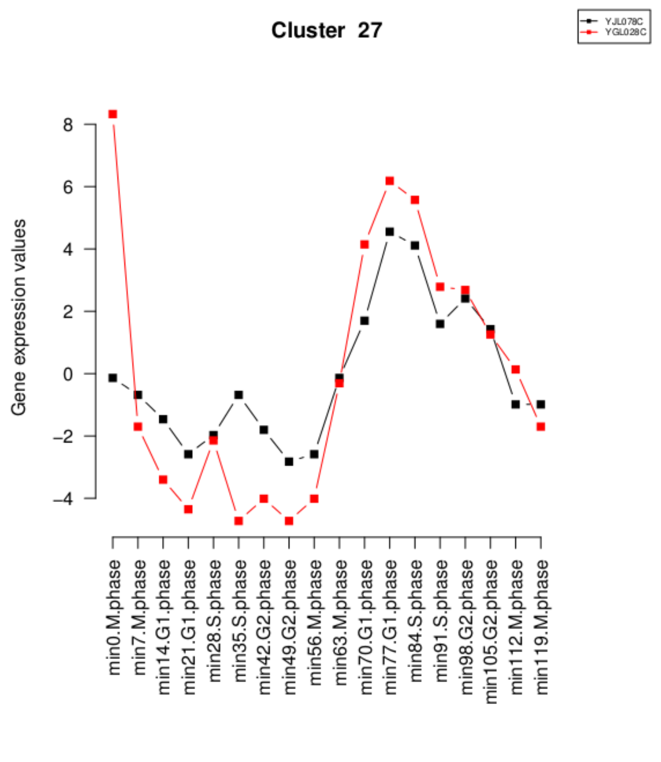
Cluster 27
YJL078C
YGL028C
Gene expression values
8
6
4
2
0
−2
−4
min0.M.phase
min7.M.phase
min14.G1.phase
min21.G1.phase
min28.S.phase
min35.S.phase
min42.G2.phase
min49.G2.phase
min56.M.phase
min63.M.phase
min70.G1.phase
min77.G1.phase
min84.S.phase
min91.S.phase
min98.G2.phase
min105.G2.phase
min112.M.phase
min119.M.phase

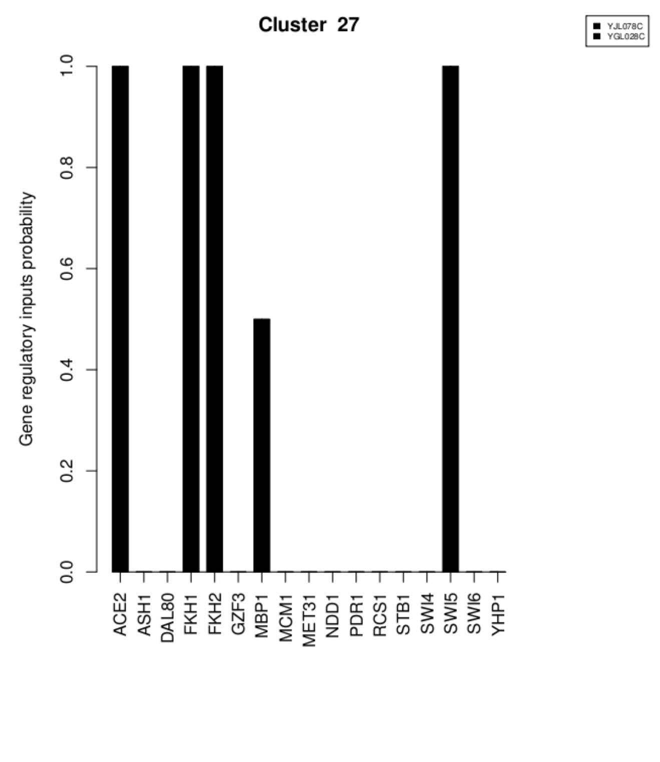
Cluster 27
YJL078C
YGL028C
Gene regulatory inputs probability
1.0
0.8
0.6
0.4
0.2
0.0
ACE2
ASH1
DAL80
FKH1
FKH2
GZF3
MBP1
MCM1
MET31
NDD1
PDR1
RCS1
STB1
SWI4
SWI5
SWI6
YHP1

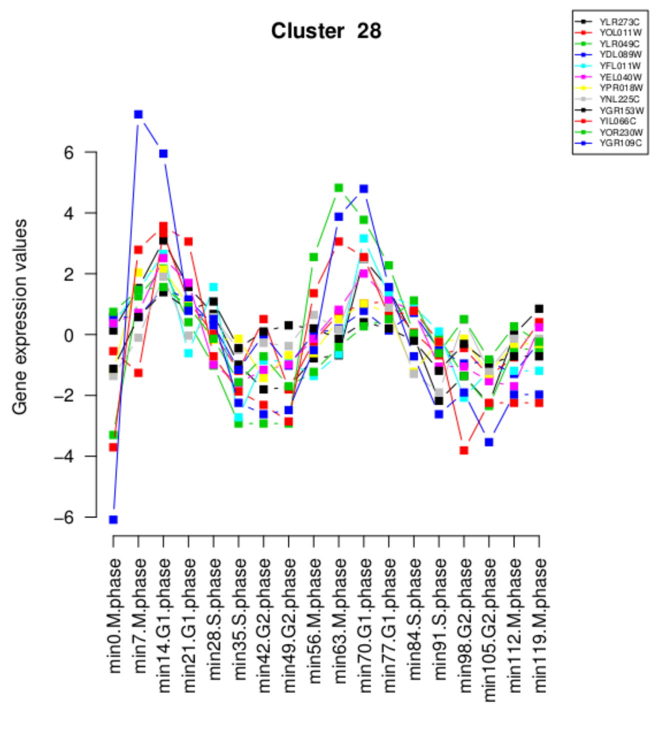
Cluster 28
YLR273C
YOL011W
YLR049C
YDL089W
YFL011W
YEL040W
YPR018W
YNL225C
YGR153W
YIL066C
YOR230W
YGR109C
Gene expression values
6
4
2
0
−2
−4
−6
min0.M.phase
min7.M.phase
min14.G1.phase
min21.G1.phase
min28.S.phase
min35.S.phase
min42.G2.phase
min49.G2.phase
min56.M.phase
min63.M.phase
min70.G1.phase
min77.G1.phase
min84.S.phase
min91.S.phase
min98.G2.phase
min105.G2.phase
min112.M.phase
min119.M.phase

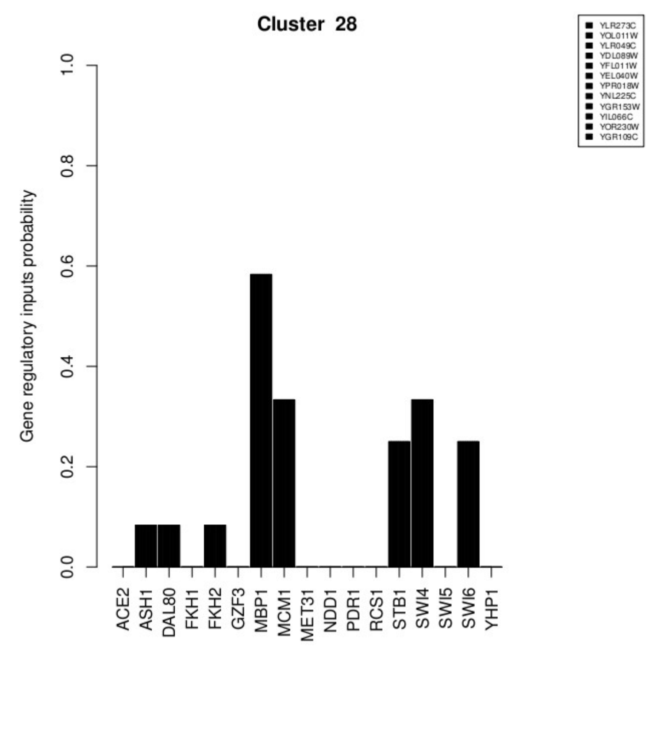
Cluster 28
YLR273C
YOL011W
YLR049C
YDL089W
YFL011W
YEL040W
YPR018W
YNL225C
YGR153W
YIL066C
YOR230W
YGR109C
Gene regulatory inputs probability
1.0
0.8
0.6
0.4
0.2
0.0
ACE2
ASH1
DAL80
FKH1
FKH2
GZF3
MBP1
MCM1
MET31
NDD1
PDR1
RCS1
STB1
SWI4
SWI5
SWI6
YHP1

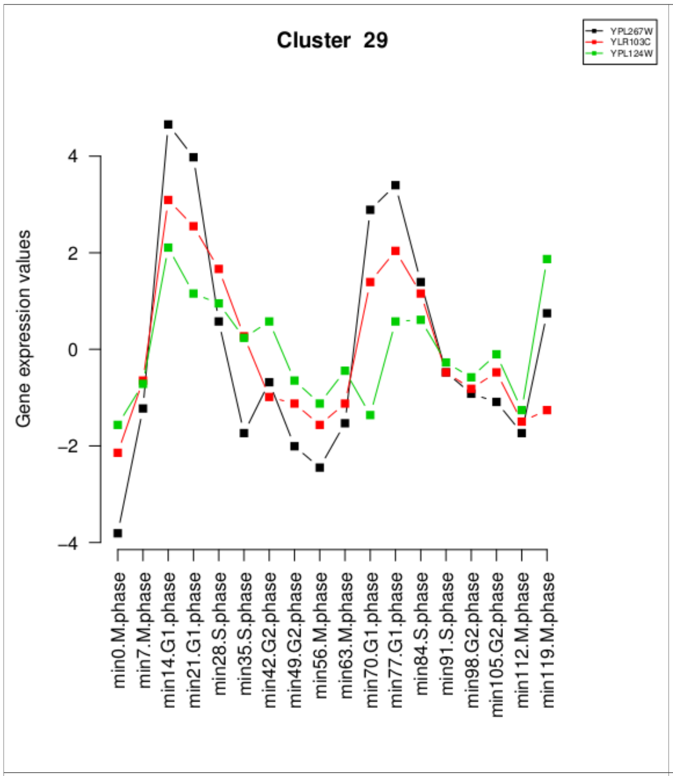
Cluster 29
YPL267W
YLR103C
YPL124W
Gene expression values
4
2
0
−2
−4
min0.M.phase
min7.M.phase
min14.G1.phase
min21.G1.phase
min28.S.phase
min35.S.phase
min42.G2.phase
min49.G2.phase
min56.M.phase
min63.M.phase
min70.G1.phase
min77.G1.phase
min84.S.phase
min91.S.phase
min98.G2.phase
min105.G2.phase
min112.M.phase
min119.M.phase

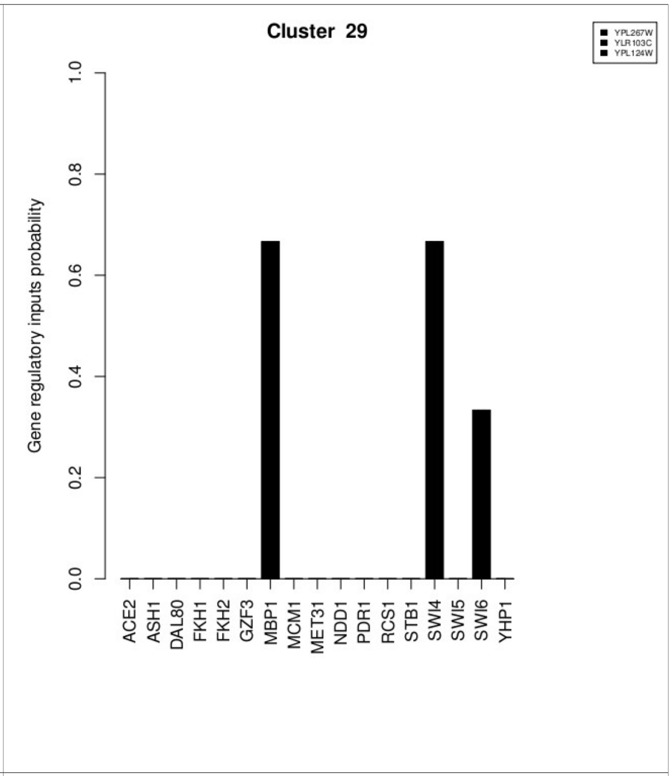
Cluster 29
YPL267W
YLR103C
YPL124W
Gene regulatory inputs probability
0.0
0.2
0.4
0.6
0.8
1.0
ACE2
ASH1
DAL80
FKH1
FKH2
GZF3
MBP1
MCM1
MET31
NDD1
PDR1
RCS1
STB1
SWI4
SWI5
SWI6
YHP1

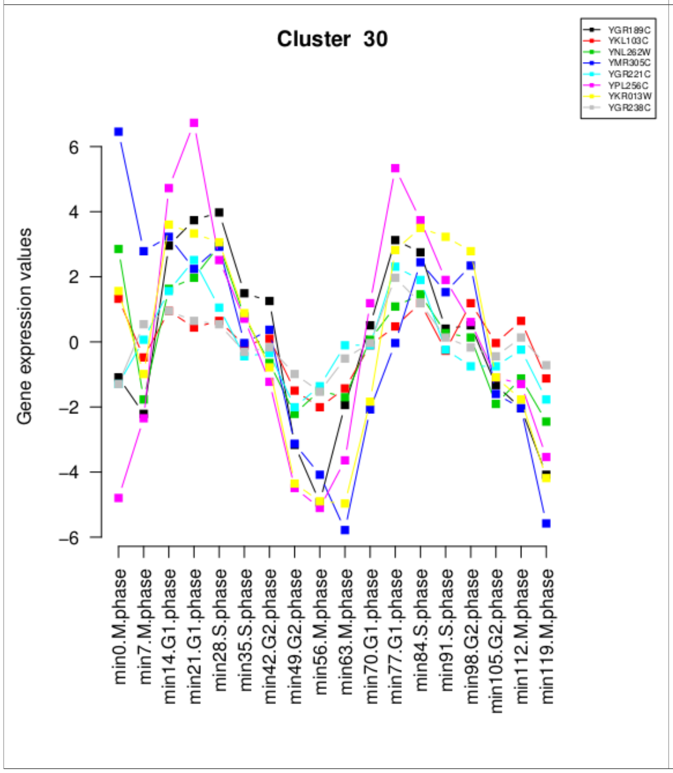
Cluster 30
YGR189C
YKL103C
YNL262W
YMR305C
YGR221C
YPL256C
YKR013W
YGR238C
Gene expression values
6
4
2
0
−2
−4
−6
min0.M.phase
min7.M.phase
min14.G1.phase
min21.G1.phase
min28.S.phase
min35.S.phase
min42.G2.phase
min49.G2.phase
min56.M.phase
min63.M.phase
min70.G1.phase
min77.G1.phase
min84.S.phase
min91.S.phase
min98.G2.phase
min105.G2.phase
min112.M.phase
min119.M.phase

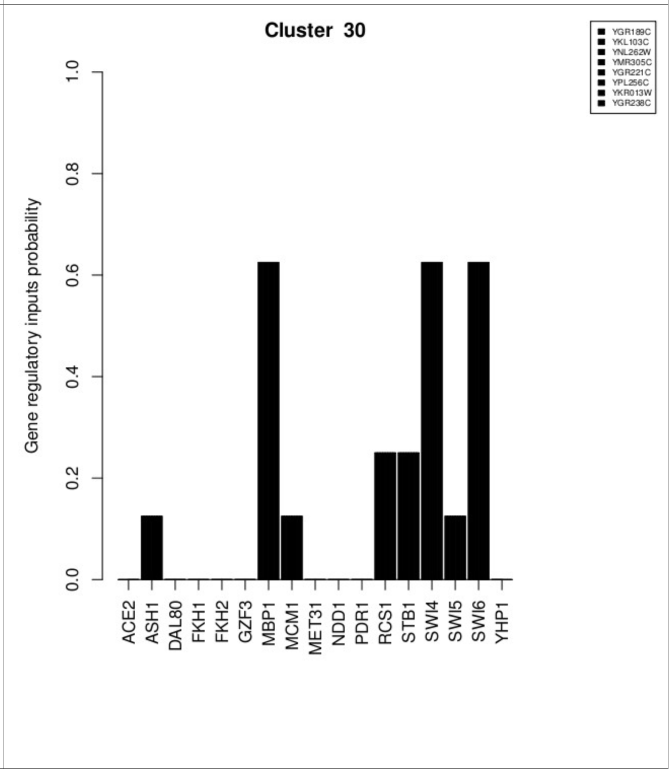
Cluster 30
YGR189C
YKL103C
YNL262W
YMR305C
YGR221C
YPL256C
YKR013W
YGR238C
Gene regulatory inputs probability
0.0
0.2
0.4
0.6
0.8
1.0
ACE2
ASH1
DAL80
FKH1
FKH2
GZF3
MBP1
MCM1
MET31
NDD1
PDR1
RCS1
STB1
SWI4
SWI5
SWI6
YHP1

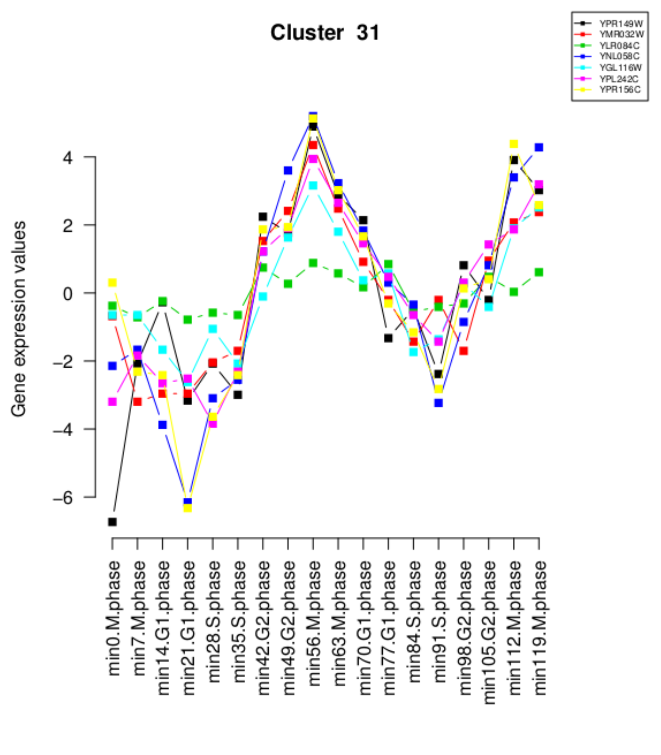

Cluster 31
YPR149W
YMR032W
YLR084C
YNL058C
YGL116W
YPL242C
YPR156C
Gene expression values
4
2
0
−2
−4
−6
min0.M.phase
min7.M.phase
min14.G1.phase
min21.G1.phase
min28.S.phase
min35.S.phase
min42.G2.phase
min49.G2.phase
min56.M.phase
min63.M.phase
min70.G1.phase
min77.G1.phase
min84.S.phase
min91.S.phase
min98.G2.phase
min105.G2.phase
min112.M.phase
min119.M.phase

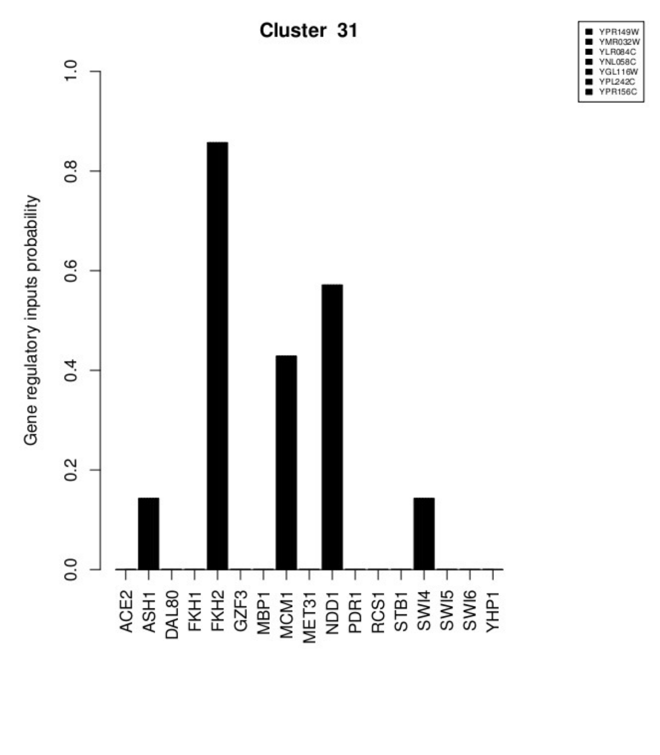

Cluster 31
YPR149W
YMR032W
YLR084C
YNL058C
YGL116W
YPL242C
YPR156C
Gene regulatory inputs probability
1.0
0.8
0.6
0.4
0.2
0.0
ACE2
ASH1
DAL80
FKH1
FKH2
GZF3
MBP1
MCM1
MET31
NDD1
PDR1
RCS1
STB1
SWI4
SWI5
SWI6
YHP1

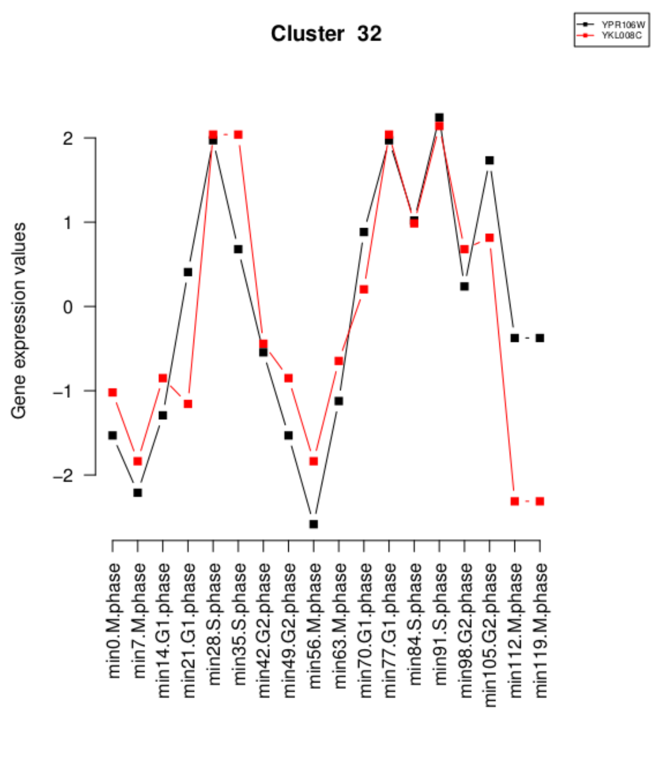

Cluster 32
YPR106W
YKL008C
Gene expression values
2
1
0
−1
−2
min0.M.phase
min7.M.phase
min14.G1.phase
min21.G1.phase
min28.S.phase
min35.S.phase
min42.G2.phase
min49.G2.phase
min56.M.phase
min63.M.phase
min70.G1.phase
min77.G1.phase
min84.S.phase
min91.S.phase
min98.G2.phase
min105.G2.phase
min112.M.phase
min119.M.phase

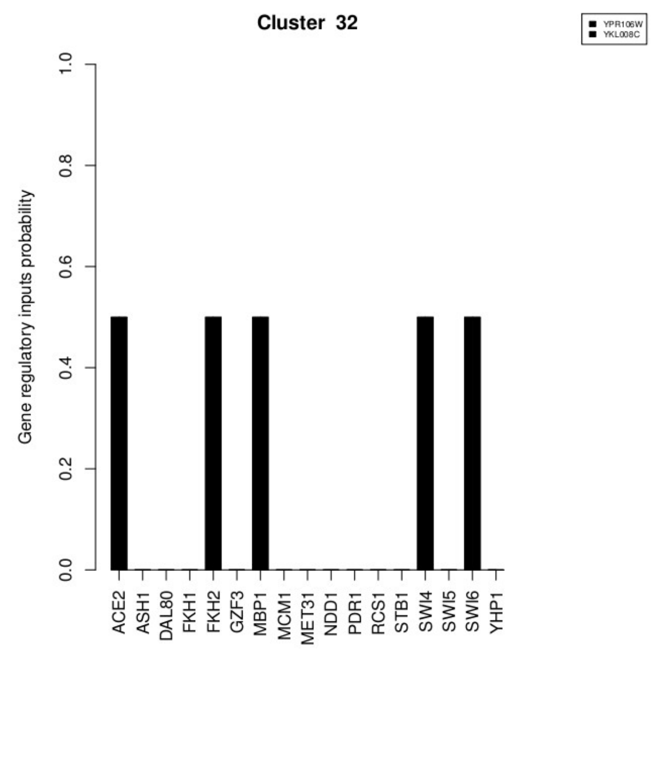

Cluster 32
YPR106W
YKL008C
Gene regulatory inputs probability
1.0
0.8
0.6
0.4
0.2
0.0
ACE2
ASH1
DAL80
FKH1
FKH2
GZF3
MBP1
MCM1
MET31
NDD1
PDR1
RCS1
STB1
SWI4
SWI5
SWI6
YHP1

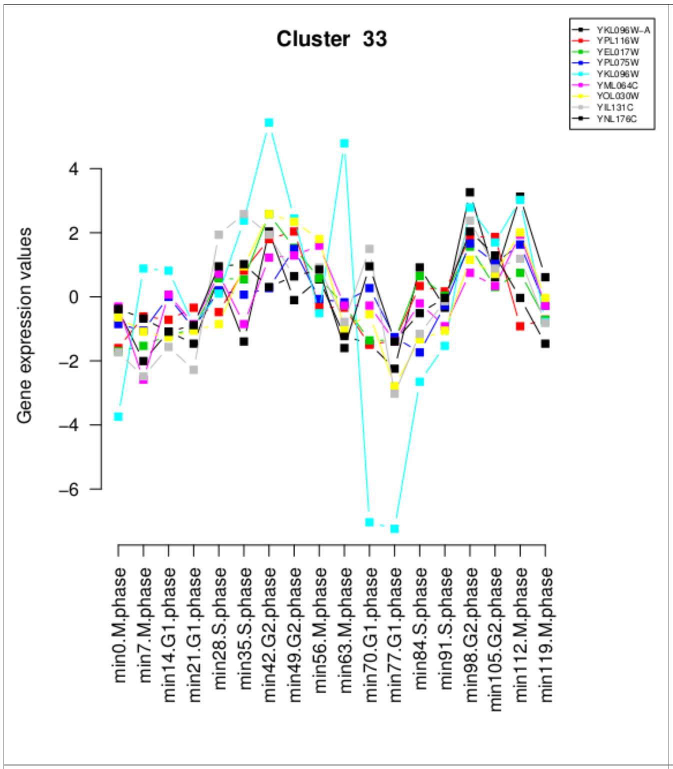
Cluster 33
YKL096W-A
YPL116W
YEL017W
YPL075W
YKL096W
YML064C
YOL030W
YIL131C
YNL176C
Gene expression values
4
2
0
−2
−4
−6
min0.M.phase
min7.M.phase
min14.G1.phase
min21.G1.phase
min28.S.phase
min35.S.phase
min42.G2.phase
min49.G2.phase
min56.M.phase
min63.M.phase
min70.G1.phase
min77.G1.phase
min84.S.phase
min91.S.phase
min98.G2.phase
min105.G2.phase
min112.M.phase
min119.M.phase

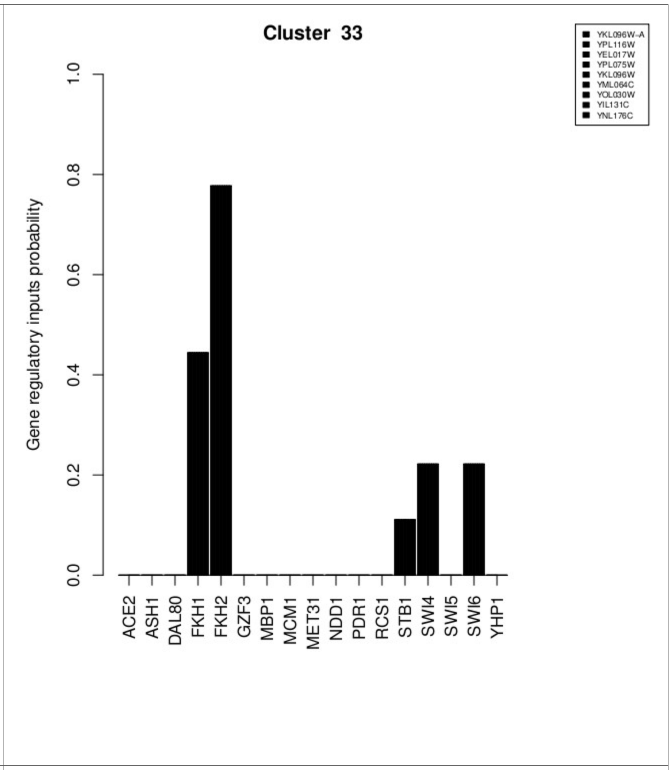
Cluster 33
YKL096W-A
YPL116W
YEL017W
YPL075W
YKL096W
YML064C
YOL030W
YIL131C
YNL176C
Gene regulatory inputs probability
1.0
0.8
0.6
0.4
0.2
0.0
ACE2
ASH1
DAL80
FKH1
FKH2
GZF3
MBP1
MCM1
MET31
NDD1
PDR1
RCS1
STB1
SWI4
SWI5
SWI6
YHP1

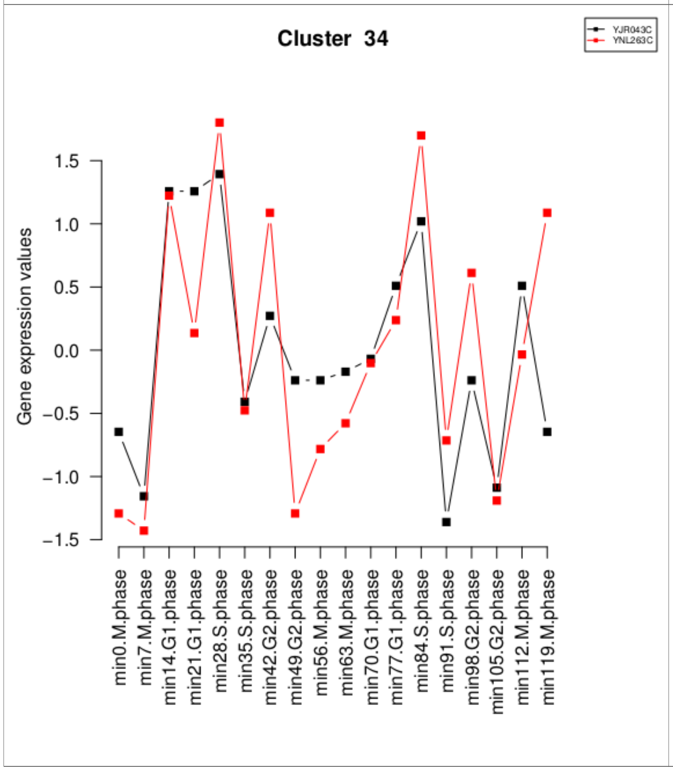
Cluster 34
YJR043C
YNL263C
Gene expression values
1.5
1.0
0.5
0.0
−0.5
−1.0
−1.5
min0.M.phase
min7.M.phase
min14.G1.phase
min21.G1.phase
min28.S.phase
min35.S.phase
min42.G2.phase
min49.G2.phase
min56.M.phase
min63.M.phase
min70.G1.phase
min77.G1.phase
min84.S.phase
min91.S.phase
min98.G2.phase
min105.G2.phase
min112.M.phase
min119.M.phase

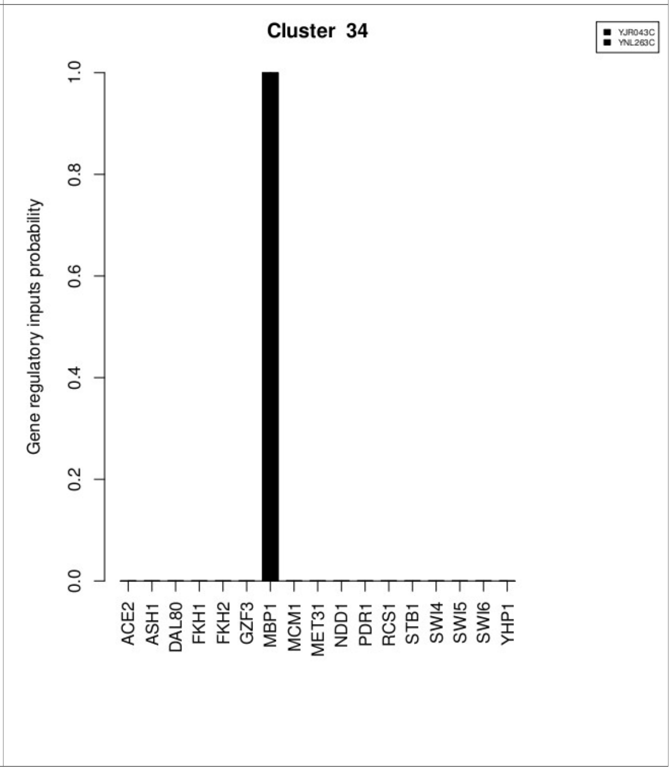
Cluster 34
YJR043C
YNL263C
Gene regulatory inputs probability
1.0
0.8
0.6
0.4
0.2
0.0
ACE2
ASH1
DAL80
FKH1
FKH2
GZF3
MBP1
MCM1
MET31
NDD1
PDR1
RCS1
STB1
SWI4
SWI5
SWI6
YHP1

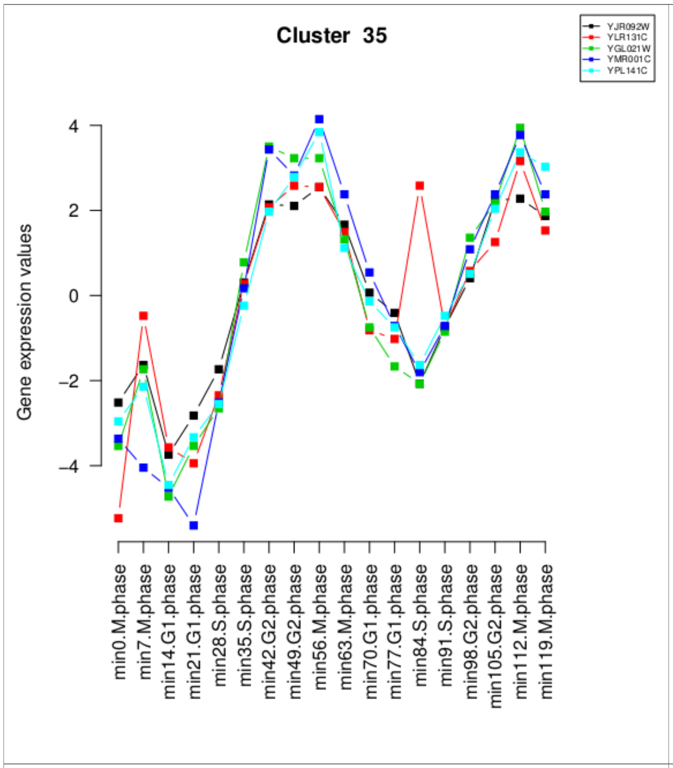
Cluster 35
YJR092W
YLR131C
YGL021W
YMR001C
YPL141C
Gene expression values
4
2
0
−2
−4
min0.M.phase
min7.M.phase
min14.G1.phase
min21.G1.phase
min28.S.phase
min35.S.phase
min42.G2.phase
min49.G2.phase
min56.M.phase
min63.M.phase
min70.G1.phase
min77.G1.phase
min84.S.phase
min91.S.phase
min98.G2.phase
min105.G2.phase
min112.M.phase
min119.M.phase

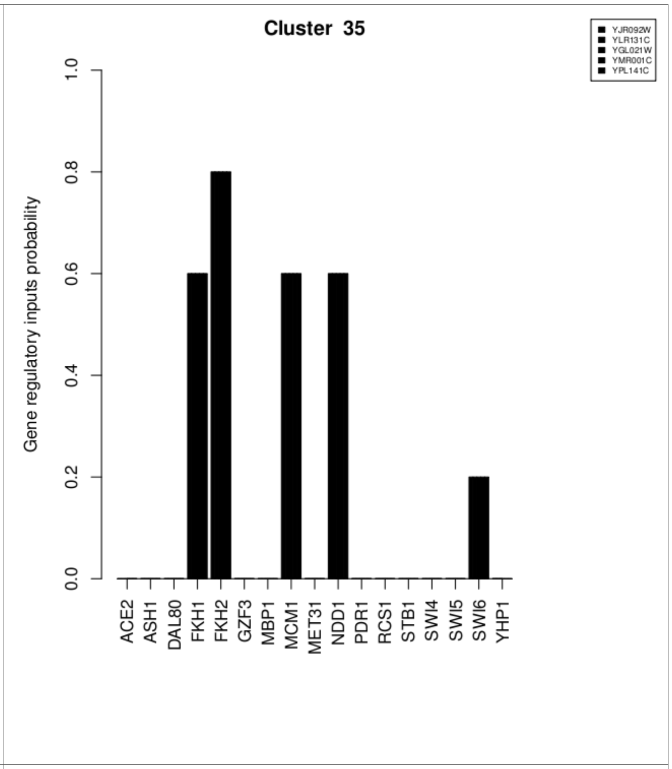
Cluster 35
YJR092W
YLR131C
YGL021W
YMR001C
YPL141C
Gene regulatory inputs probability
0.0
0.2
0.4
0.6
0.8
1.0
ACE2
ASH1
DAL80
FKH1
FKH2
GZF3
MBP1
MCM1
MET31
NDD1
PDR1
RCS1
STB1
SWI4
SWI5
SWI6
YHP1

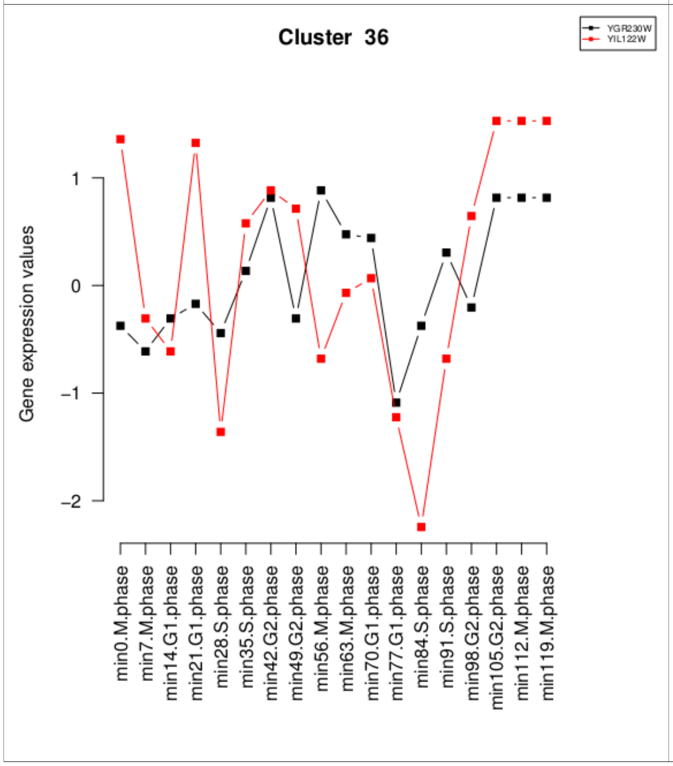
Cluster 36
YGR230W
YIL122W
Gene expression values
1
0
−1
−2
min0.M.phase
min7.M.phase
min14.G1.phase
min21.G1.phase
min28.S.phase
min35.S.phase
min42.G2.phase
min49.G2.phase
min56.M.phase
min63.M.phase
min70.G1.phase
min77.G1.phase
min84.S.phase
min91.S.phase
min98.G2.phase
min105.G2.phase
min112.M.phase
min119.M.phase

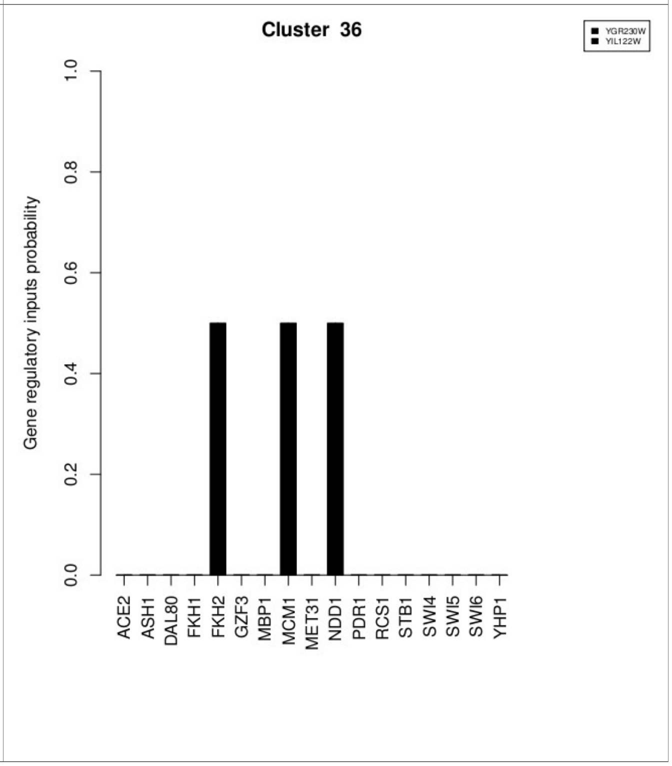
Cluster 36
YGR230W
YIL122W
Gene regulatory inputs probability
0.0
0.2
0.4
0.6
0.8
1.0
ACE2
ASH1
DAL80
FKH1
FKH2
GZF3
MBP1
MCM1
MET31
NDD1
PDR1
RCS1
STB1
SWI4
SWI5
SWI6
YHP1

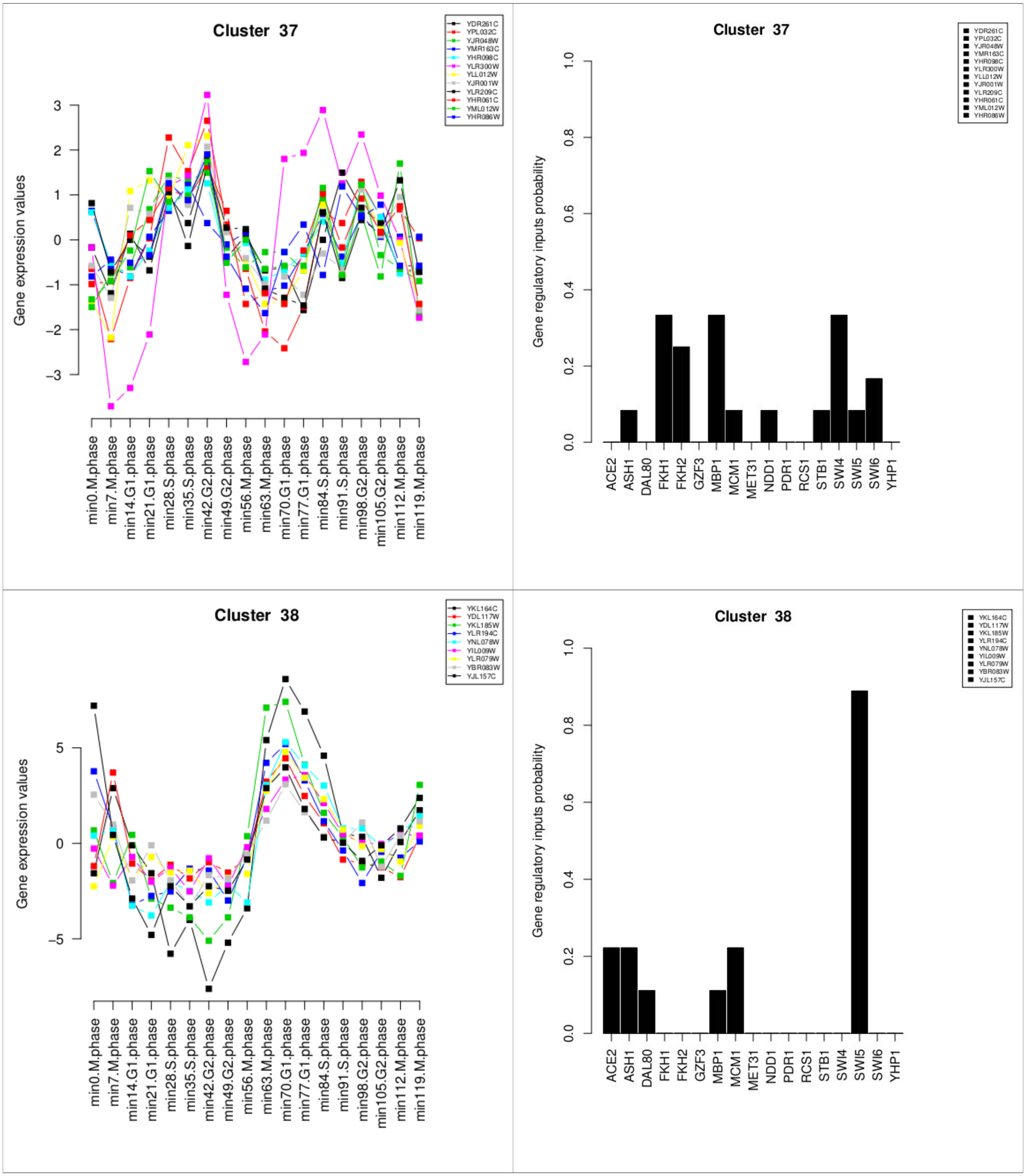
Cluster 37
Gene expression values
YDR261C
YPL032C
YJR048W
YMR163C
YHR098C
YLR300W
YLL012W
YJR001W
YLR209C
YHR061C
YML012W
YHR086W
min0.M.phase
min7.M.phase
min14.G1.phase
min21.G1.phase
min28.S.phase
min35.S.phase
min42.G2.phase
min49.G2.phase
min56.M.phase
min63.M.phase
min70.G1.phase
min77.G1.phase
min84.S.phase
min91.S.phase
min98.G2.phase
min105.G2.phase
min112.M.phase
min119.M.phase
Cluster 37
Gene regulatory inputs probability
ACE2
ASH1
DAL80
FKH1
FKH2
GZF3
MBP1
MCM1
MET31
NDD1
PDR1
RCS1
STB1
SWI4
SWI5
SWI6
YHP1
Cluster 38
Gene expression values
YKL164C
YDL117W
YKL185W
YLR194C
YNL078W
YIL009W
YLR079W
YBR083W
YJL157C
Cluster 38
Gene regulatory inputs probability

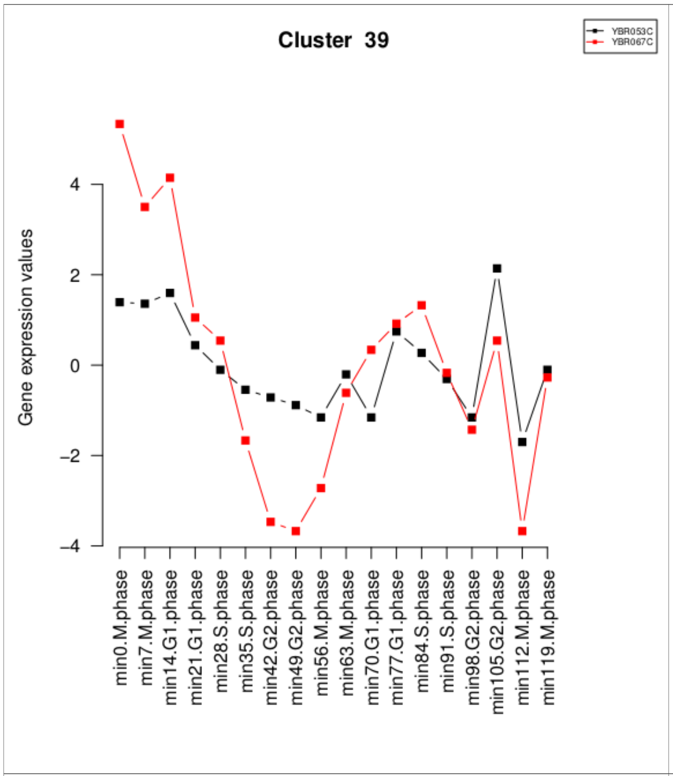
Cluster 39
YBR053C
YBR067C
Gene expression values
4
2
0
−2
−4
min0.M.phase
min7.M.phase
min14.G1.phase
min21.G1.phase
min28.S.phase
min35.S.phase
min42.G2.phase
min49.G2.phase
min56.M.phase
min63.M.phase
min70.G1.phase
min77.G1.phase
min84.S.phase
min91.S.phase
min98.G2.phase
min105.G2.phase
min112.M.phase
min119.M.phase

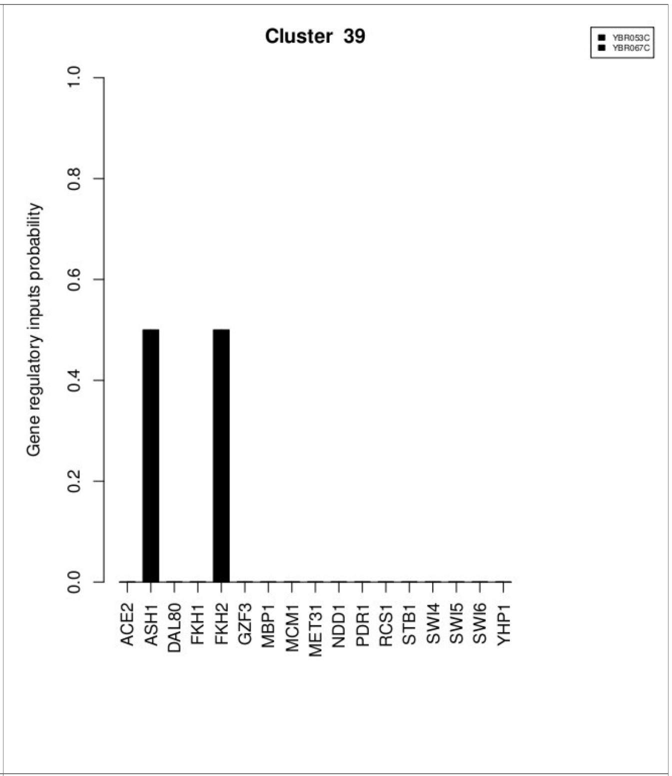
Cluster 39
YBR053C
YBR067C
Gene regulatory inputs probability
1.0
0.8
0.6
0.4
0.2
0.0
ACE2
ASH1
DAL80
FKH1
FKH2
GZF3
MBP1
MCM1
MET31
NDD1
PDR1
RCS1
STB1
SWI4
SWI5
SWI6
YHP1

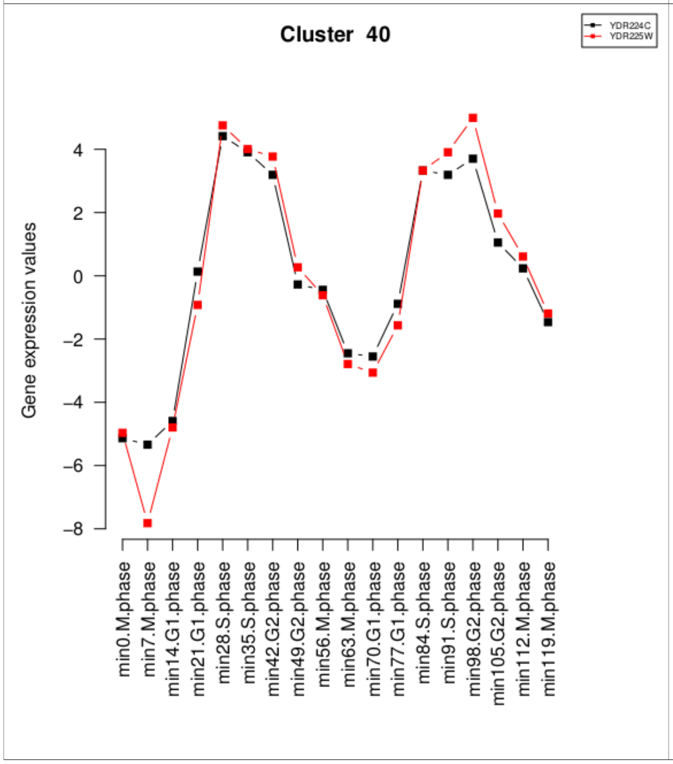
Cluster 40
YDR224C
YDR225W
Gene expression values
4
2
0
−2
−4
−6
−8
min0.M.phase
min7.M.phase
min14.G1.phase
min21.G1.phase
min28.S.phase
min35.S.phase
min42.G2.phase
min49.G2.phase
min56.M.phase
min63.M.phase
min70.G1.phase
min77.G1.phase
min84.S.phase
min91.S.phase
min98.G2.phase
min105.G2.phase
min112.M.phase
min119.M.phase

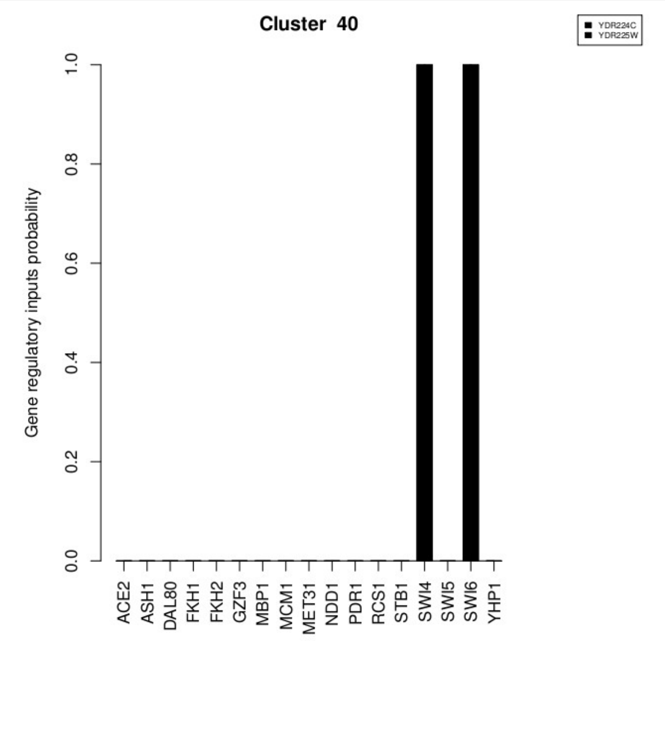
Cluster 40
YDR224C
YDR225W
Gene regulatory inputs probability
1.0
0.8
0.6
0.4
0.2
0.0
ACE2
ASH1
DAL80
FKH1
FKH2
GZF3
MBP1
MCM1
MET31
NDD1
PDR1
RCS1
STB1
SWI4
SWI5
SWI6
YHP1

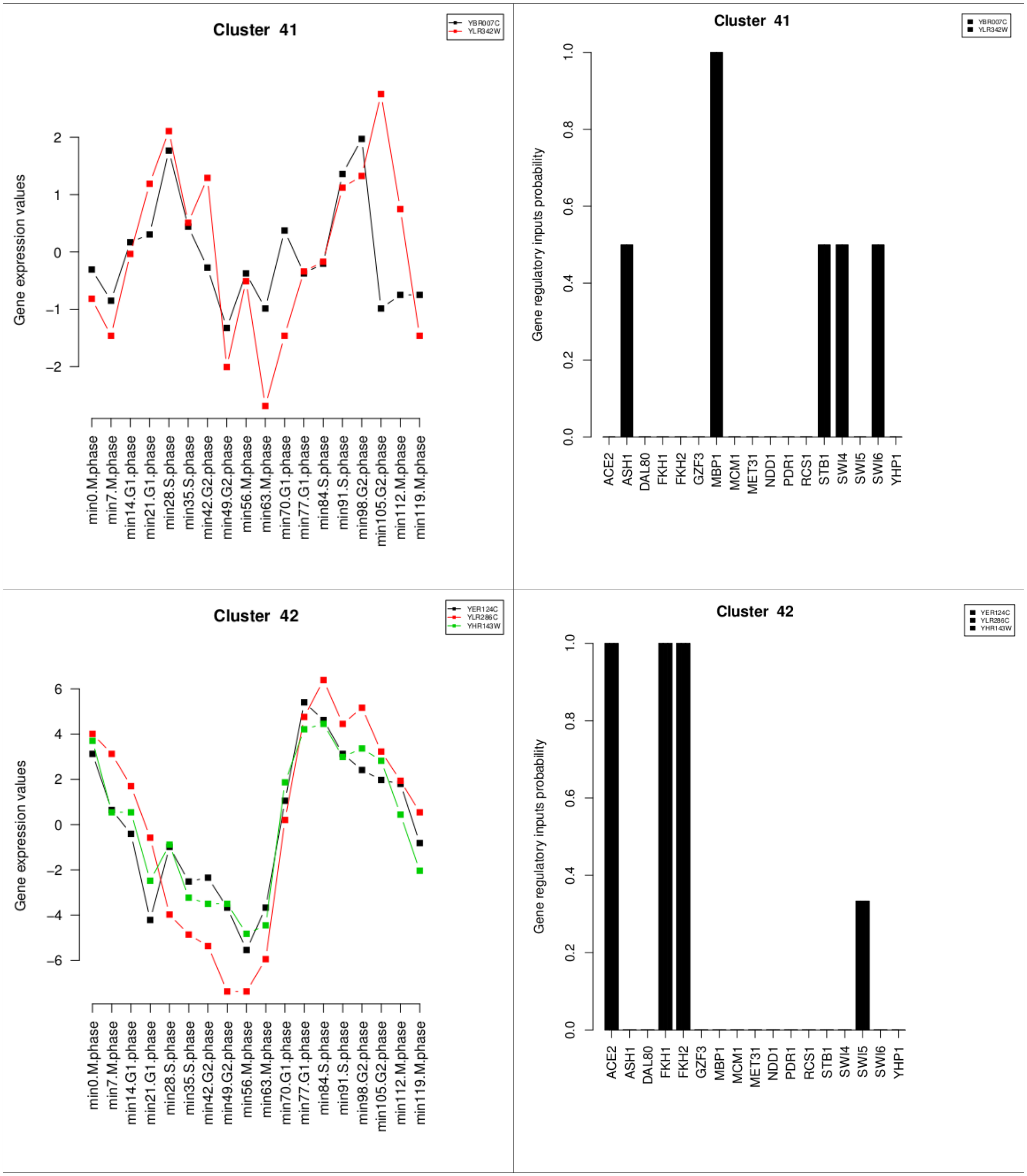

Cluster 41
YBR007C
YLR342W
Gene expression values
2
1
0
−1
−2
min0.M.phase
min7.M.phase
min14.G1.phase
min21.G1.phase
min28.S.phase
min35.S.phase
min42.G2.phase
min49.G2.phase
min56.M.phase
min63.M.phase
min70.G1.phase
min77.G1.phase
min84.S.phase
min91.S.phase
min98.G2.phase
min105.G2.phase
min112.M.phase
min119.M.phase
Cluster 41
YBR007C
YLR342W
Gene regulatory inputs probability
0.0
0.2
0.4
0.6
0.8
1.0
ACE2
ASH1
DAL80
FKH1
FKH2
GZF3
MBP1
MCM1
MET31
NDD1
PDR1
RCS1
STB1
SWI4
SWI5
SWI6
YHP1
Cluster 42
YER124C
YLR286C
YHR143W
Gene expression values
6
4
2
0
−2
−4
−6
min0.M.phase
min7.M.phase
min14.G1.phase
min21.G1.phase
min28.S.phase
min35.S.phase
min42.G2.phase
min49.G2.phase
min56.M.phase
min63.M.phase
min70.G1.phase
min77.G1.phase
min84.S.phase
min91.S.phase
min98.G2.phase
min105.G2.phase
min112.M.phase
min119.M.phase
Cluster 42
YER124C
YLR286C
YHR143W
Gene regulatory inputs probability
0.0
0.2
0.4
0.6
0.8
1.0
ACE2
ASH1
DAL80
FKH1
FKH2
GZF3
MBP1
MCM1
MET31
NDD1
PDR1
RCS1
STB1
SWI4
SWI5
SWI6
YHP1

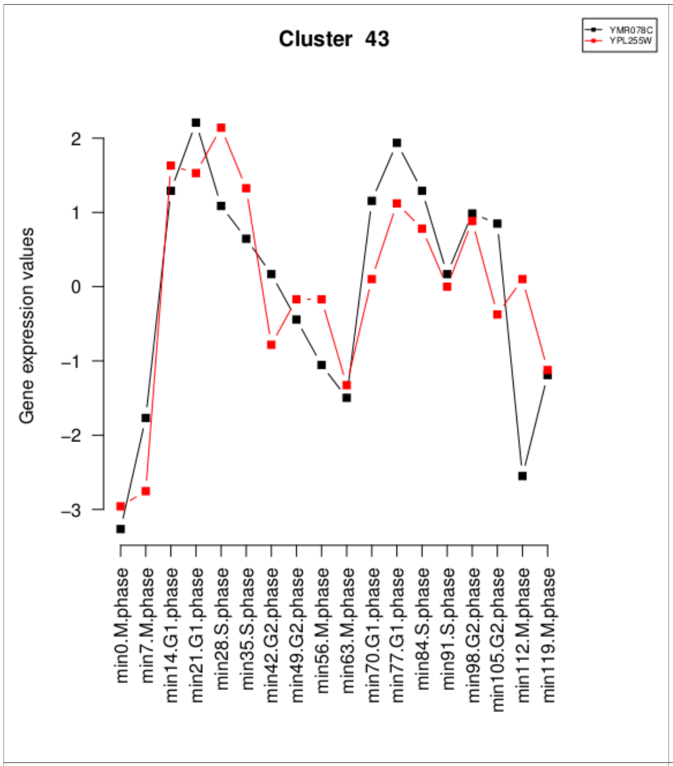
Cluster 43
YMR078C
YPL255W
Gene expression values
2
1
0
−1
−2
−3
min0.M.phase
min7.M.phase
min14.G1.phase
min21.G1.phase
min28.S.phase
min35.S.phase
min42.G2.phase
min49.G2.phase
min56.M.phase
min63.M.phase
min70.G1.phase
min77.G1.phase
min84.S.phase
min91.S.phase
min98.G2.phase
min105.G2.phase
min112.M.phase
min119.M.phase

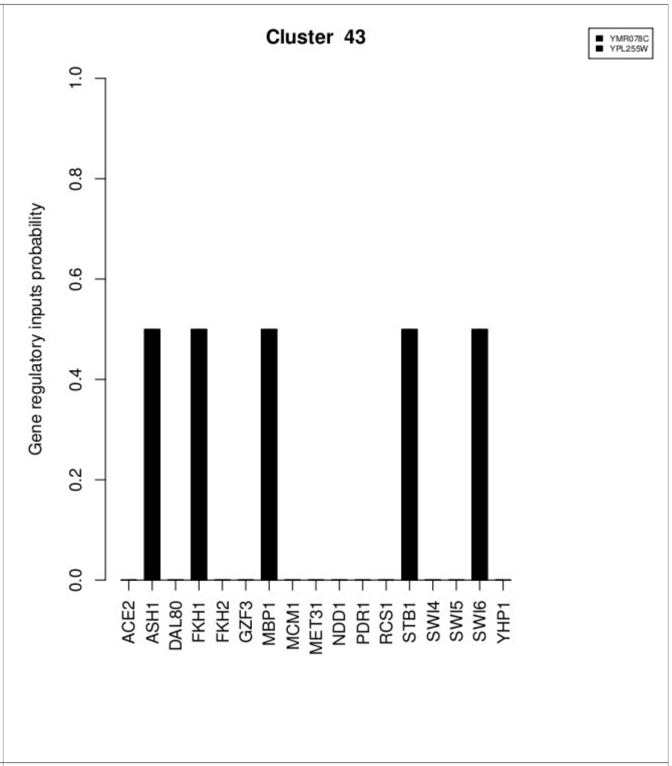
Cluster 43
YMR078C
YPL255W
Gene regulatory inputs probability
0.0
0.2
0.4
0.6
0.8
1.0
ACE2
ASH1
DAL80
FKH1
FKH2
GZF3
MBP1
MCM1
MET31
NDD1
PDR1
RCS1
STB1
SWI4
SWI5
SWI6
YHP1

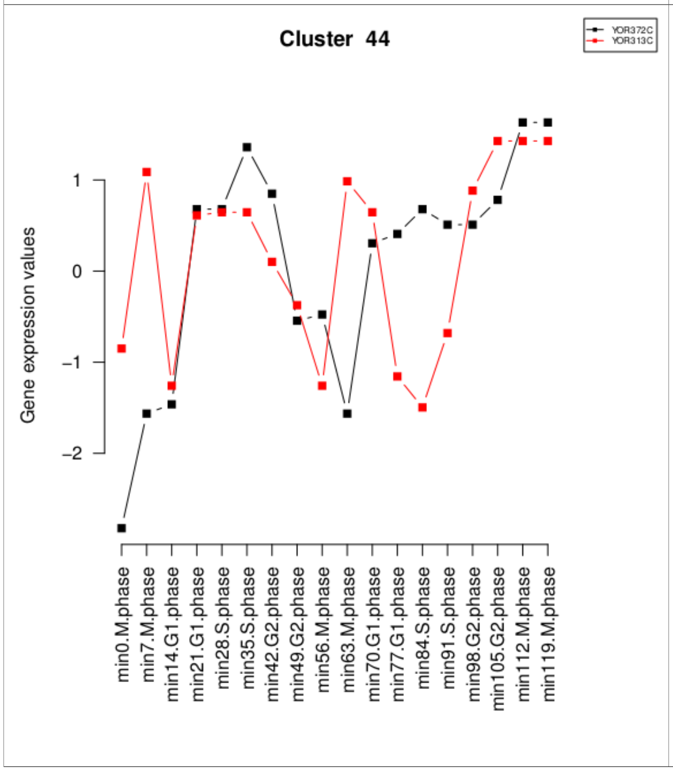
Cluster 44
YOR372C
YOR313C
Gene expression values
1
0
−1
−2
min0.M.phase
min7.M.phase
min14.G1.phase
min21.G1.phase
min28.S.phase
min35.S.phase
min42.G2.phase
min49.G2.phase
min56.M.phase
min63.M.phase
min70.G1.phase
min77.G1.phase
min84.S.phase
min91.S.phase
min98.G2.phase
min105.G2.phase
min112.M.phase
min119.M.phase

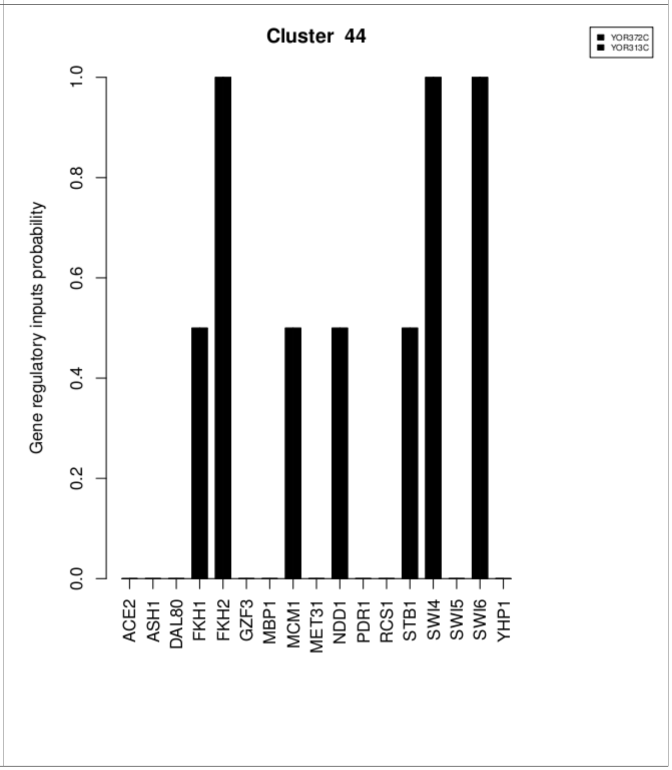
Cluster 44
YOR372C
YOR313C
Gene regulatory inputs probability
0.0
0.2
0.4
0.6
0.8
1.0
ACE2
ASH1
DAL80
FKH1
FKH2
GZF3
MBP1
MCM1
MET31
NDD1
PDR1
RCS1
STB1
SWI4
SWI5
SWI6
YHP1

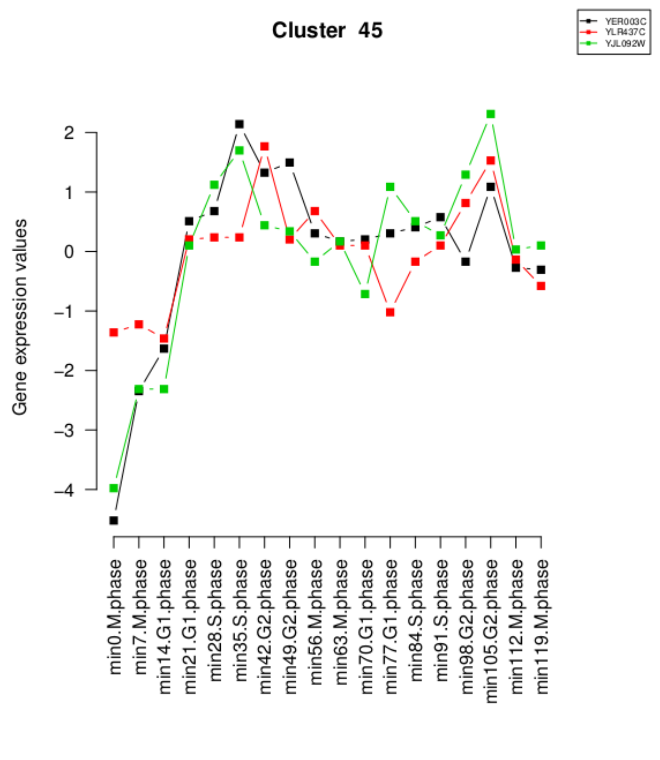
Cluster 45
YER003C
YLR437C
YJL092W
Gene expression values
2
1
0
−1
−2
−3
−4
min0.M.phase
min7.M.phase
min14.G1.phase
min21.G1.phase
min28.S.phase
min35.S.phase
min42.G2.phase
min49.G2.phase
min56.M.phase
min63.M.phase
min70.G1.phase
min77.G1.phase
min84.S.phase
min91.S.phase
min98.G2.phase
min105.G2.phase
min112.M.phase
min119.M.phase

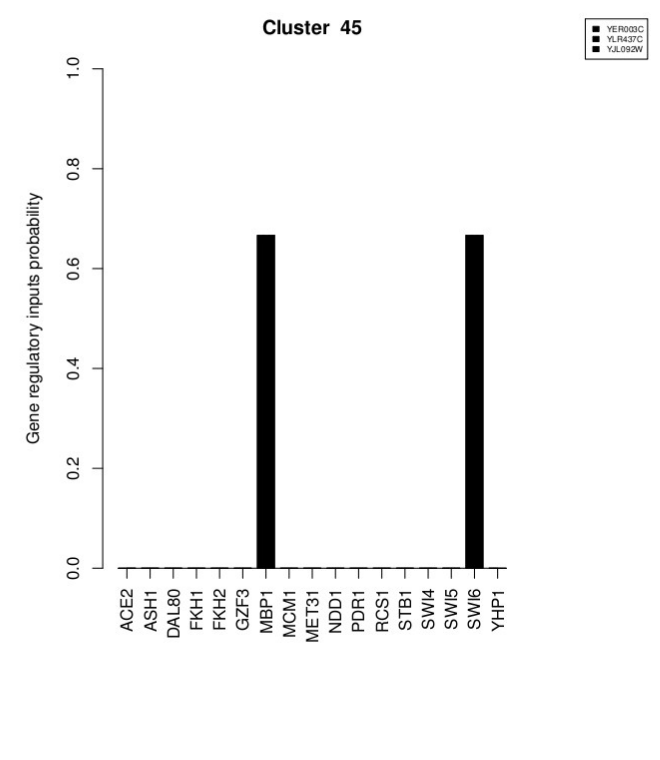
Cluster 45
YER003C
YLR437C
YJL092W
Gene regulatory inputs probability
1.0
0.8
0.6
0.4
0.2
0.0
ACE2
ASH1
DAL80
FKH1
FKH2
GZF3
MBP1
MCM1
MET31
NDD1
PDR1
RCS1
STB1
SWI4
SWI5
SWI6
YHP1

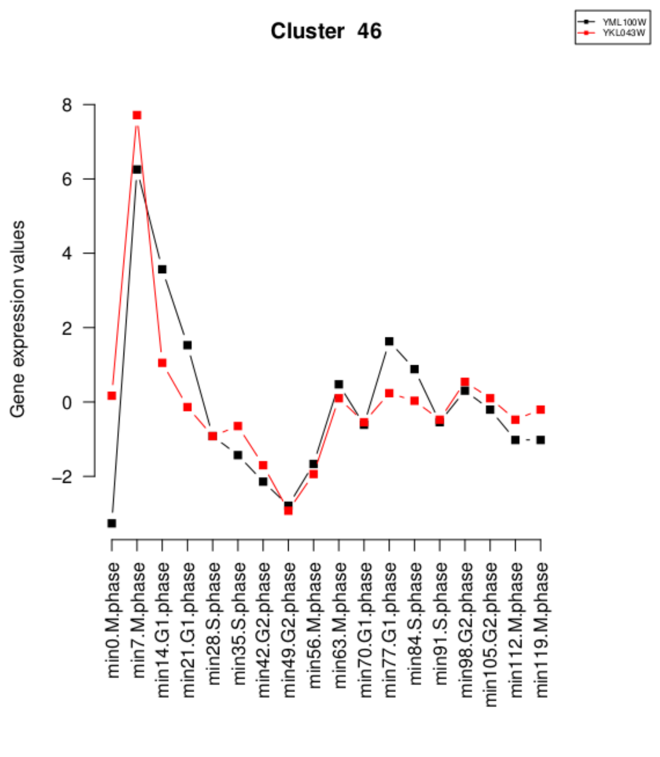
Cluster 46
YML100W
YKL043W
Gene expression values
8
6
4
2
0
−2
min0.M.phase
min7.M.phase
min14.G1.phase
min21.G1.phase
min28.S.phase
min35.S.phase
min42.G2.phase
min49.G2.phase
min56.M.phase
min63.M.phase
min70.G1.phase
min77.G1.phase
min84.S.phase
min91.S.phase
min98.G2.phase
min105.G2.phase
min112.M.phase
min119.M.phase

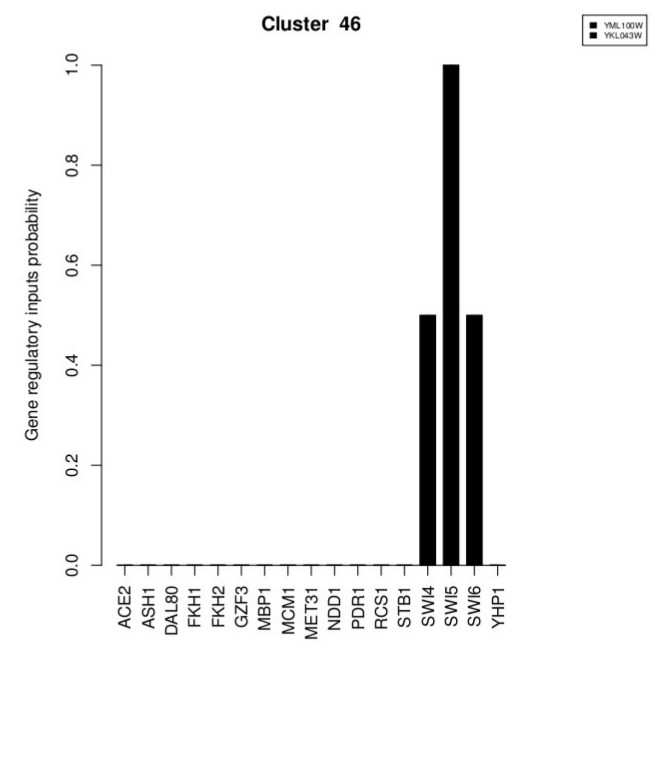
Cluster 46
YML100W
YKL043W
Gene regulatory inputs probability
1.0
0.8
0.6
0.4
0.2
0.0
ACE2
ASH1
DAL80
FKH1
FKH2
GZF3
MBP1
MCM1
MET31
NDD1
PDR1
RCS1
STB1
SWI4
SWI5
SWI6
YHP1

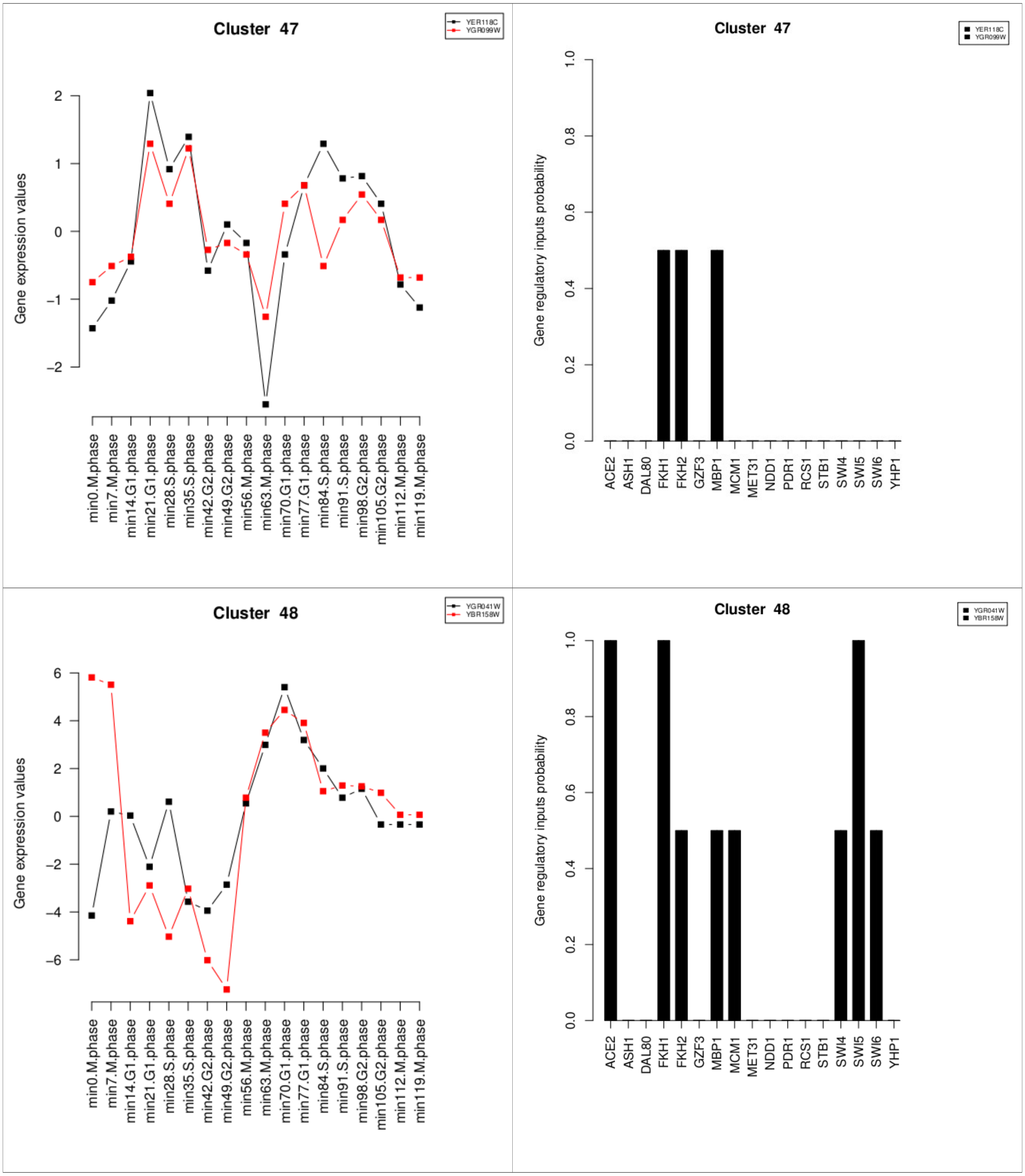

Cluster 47
YER118C
YGR099W
Gene expression values
min0.M.phase
min7.M.phase
min14.G1.phase
min21.G1.phase
min28.S.phase
min35.S.phase
min42.G2.phase
min49.G2.phase
min56.M.phase
min63.M.phase
min70.G1.phase
min77.G1.phase
min84.S.phase
min91.S.phase
min98.G2.phase
min105.G2.phase
min112.M.phase
min119.M.phase
Gene regulatory inputs probability
ACE2
ASH1
DAL80
FKH1
FKH2
GZF3
MBP1
MCM1
MET31
NDD1
PDR1
RCS1
STB1
SWI4
SWI5
SWI6
YHP1
Cluster 48
YGR041W
YBR158W

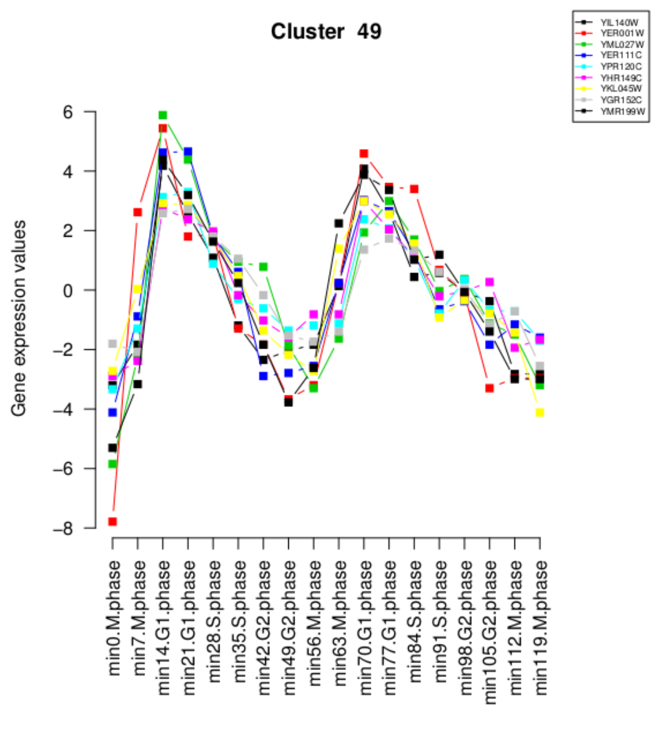
Cluster 49
Gene expression values
YIL140W
YER001W
YML027W
YER111C
YPR120C
YHR149C
YKL045W
YGR152C
YMR199W
min0.M.phase
min7.M.phase
min14.G1.phase
min21.G1.phase
min28.S.phase
min35.S.phase
min42.G2.phase
min49.G2.phase
min56.M.phase
min63.M.phase
min70.G1.phase
min77.G1.phase
min84.S.phase
min91.S.phase
min98.G2.phase
min105.G2.phase
min112.M.phase
min119.M.phase

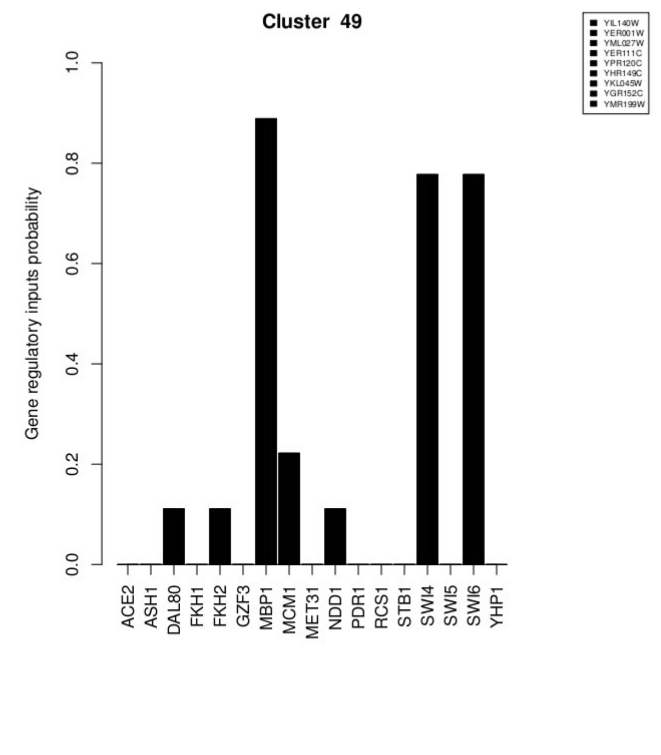
Cluster 49
Gene regulatory inputs probability
ACE2
ASH1
DAL80
FKH1
FKH2
GZF3
MBP1
MCM1
MET31
NDD1
PDR1
RCS1
STB1
SWI4
SWI5
SWI6
YHP1

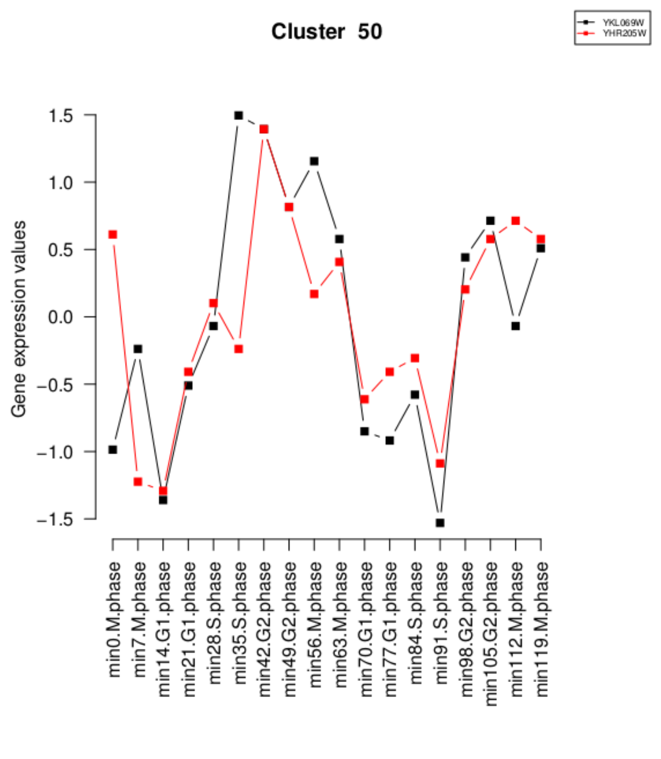
Cluster 50
Gene expression values
YKL069W
YHR205W
min0.M.phase
min7.M.phase
min14.G1.phase
min21.G1.phase
min28.S.phase
min35.S.phase
min42.G2.phase
min49.G2.phase
min56.M.phase
min63.M.phase
min70.G1.phase
min77.G1.phase
min84.S.phase
min91.S.phase
min98.G2.phase
min105.G2.phase
min112.M.phase
min119.M.phase

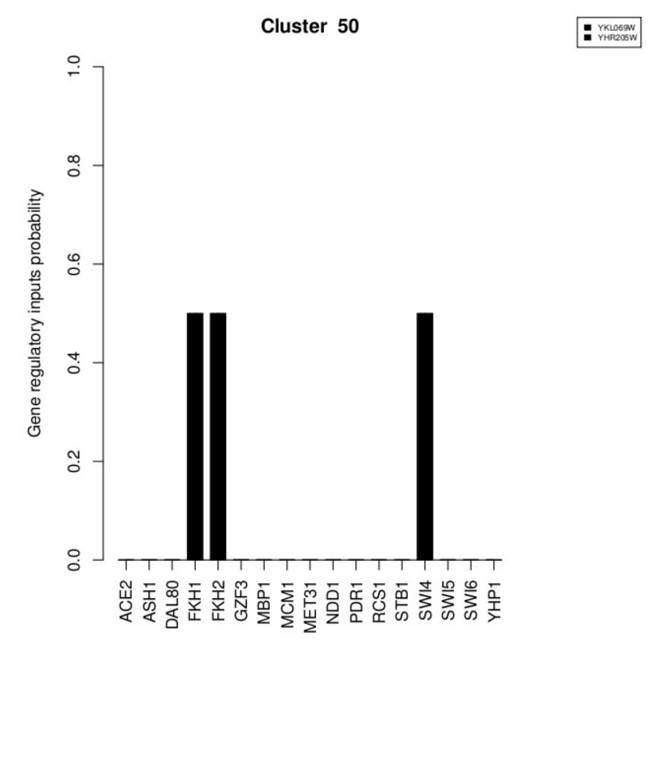
Cluster 50
Gene regulatory inputs probability
YKL069W
YHR205W
ACE2
ASH1
DAL80
FKH1
FKH2
GZF3
MBP1
MCM1
MET31
NDD1
PDR1
RCS1
STB1
SWI4
SWI5
SWI6
YHP1

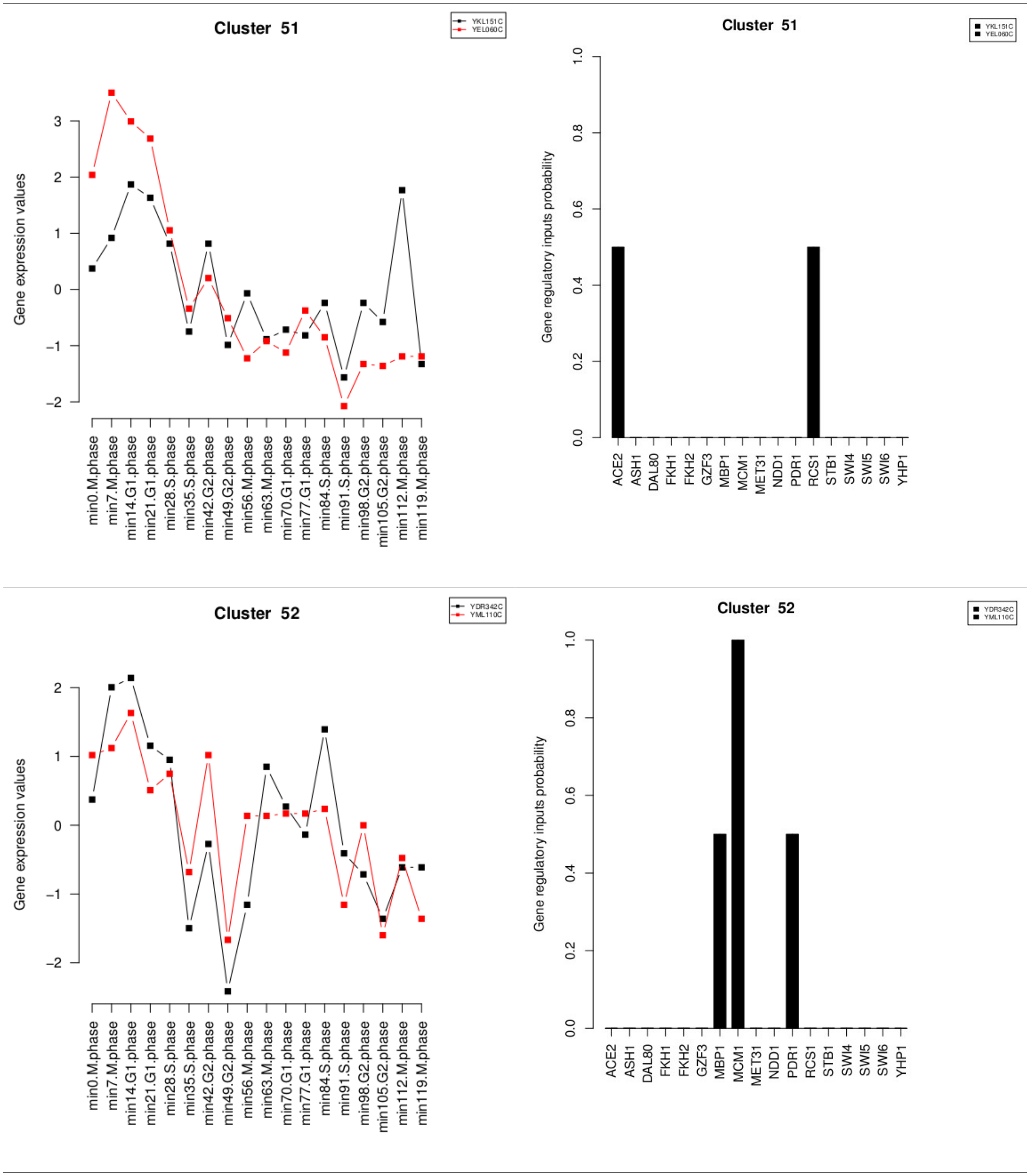

Cluster 51
YKL151C
YEL060C
Gene expression values
min0.M.phase
min7.M.phase
min14.G1.phase
min21.G1.phase
min28.S.phase
min35.S.phase
min42.G2.phase
min49.G2.phase
min56.M.phase
min63.M.phase
min70.G1.phase
min77.G1.phase
min84.S.phase
min91.S.phase
min98.G2.phase
min105.G2.phase
min112.M.phase
min119.M.phase
Cluster 51
YKL151C
YEL060C
Gene regulatory inputs probability
ACE2
ASH1
DAL80
FKH1
FKH2
GZF3
MBP1
MCM1
MET31
NDD1
PDR1
RCS1
STB1
SWI4
SWI5
SWI6
YHP1
Cluster 52
YDR342C
YML110C
Gene expression values
Cluster 52
YDR342C
YML110C
Gene regulatory inputs probability

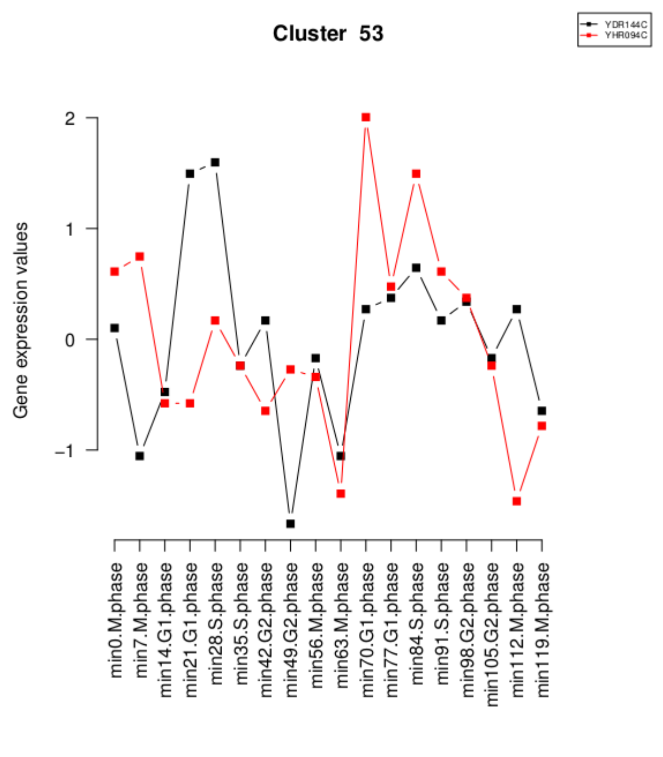
Cluster 53
YDR144C
YHR094C
Gene expression values
2
1
0
−1
min0.M.phase
min7.M.phase
min14.G1.phase
min21.G1.phase
min28.S.phase
min35.S.phase
min42.G2.phase
min49.G2.phase
min56.M.phase
min63.M.phase
min70.G1.phase
min77.G1.phase
min84.S.phase
min91.S.phase
min98.G2.phase
min105.G2.phase
min112.M.phase
min119.M.phase

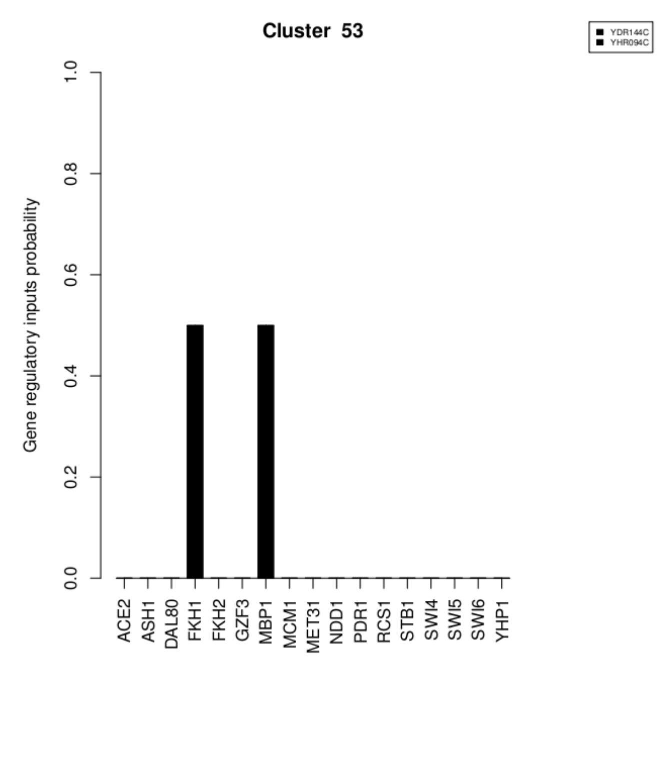
Cluster 53
YDR144C
YHR094C
Gene regulatory inputs probability
0.0
0.2
0.4
0.6
0.8
1.0
ACE2
ASH1
DAL80
FKH1
FKH2
GZF3
MBP1
MCM1
MET31
NDD1
PDR1
RCS1
STB1
SWI4
SWI5
SWI6
YHP1

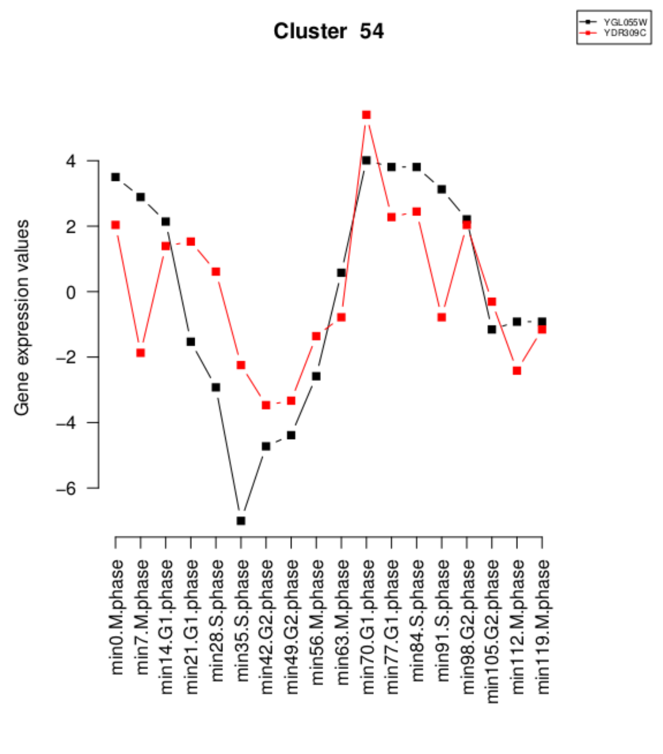
Cluster 54
YGL055W
YDR309C
Gene expression values
4
2
0
−2
−4
−6
min0.M.phase
min7.M.phase
min14.G1.phase
min21.G1.phase
min28.S.phase
min35.S.phase
min42.G2.phase
min49.G2.phase
min56.M.phase
min63.M.phase
min70.G1.phase
min77.G1.phase
min84.S.phase
min91.S.phase
min98.G2.phase
min105.G2.phase
min112.M.phase
min119.M.phase

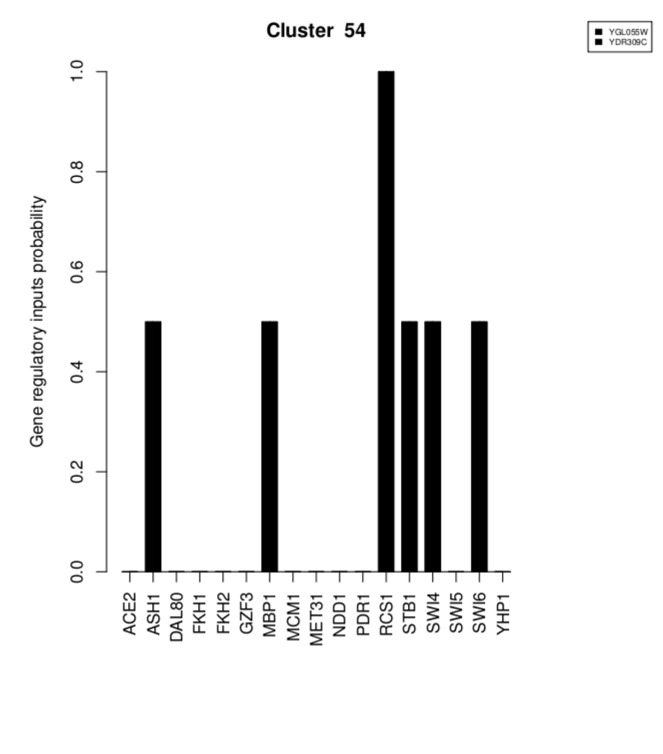
Cluster 54
YGL055W
YDR309C
Gene regulatory inputs probability
0.0
0.2
0.4
0.6
0.8
1.0
ACE2
ASH1
DAL80
FKH1
FKH2
GZF3
MBP1
MCM1
MET31
NDD1
PDR1
RCS1
STB1
SWI4
SWI5
SWI6
YHP1

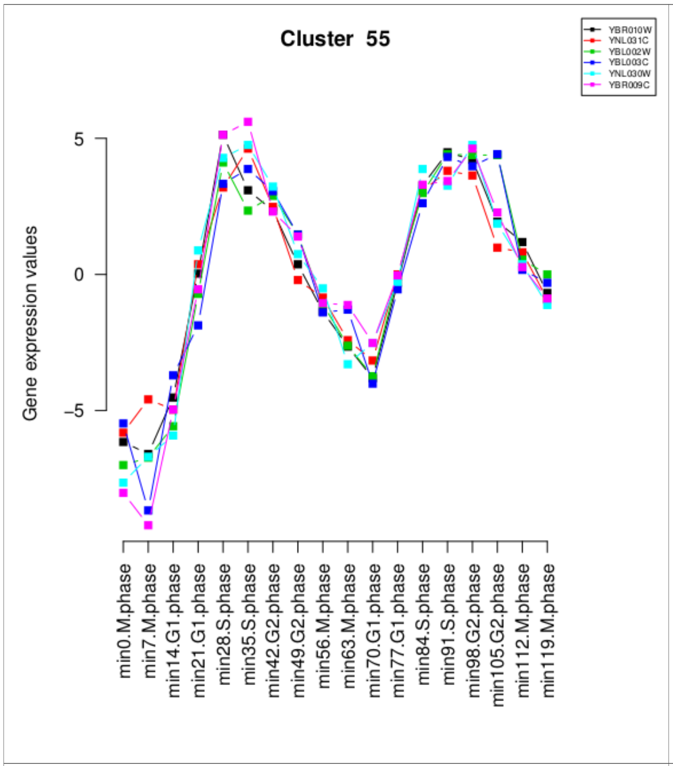
Cluster 55
YBR010W
YNL031C
YBL002W
YBL003C
YNL030W
YBR009C
Gene expression values
5
0
−5
min0.M.phase
min7.M.phase
min14.G1.phase
min21.G1.phase
min28.S.phase
min35.S.phase
min42.G2.phase
min49.G2.phase
min56.M.phase
min63.M.phase
min70.G1.phase
min77.G1.phase
min84.S.phase
min91.S.phase
min98.G2.phase
min105.G2.phase
min112.M.phase
min119.M.phase

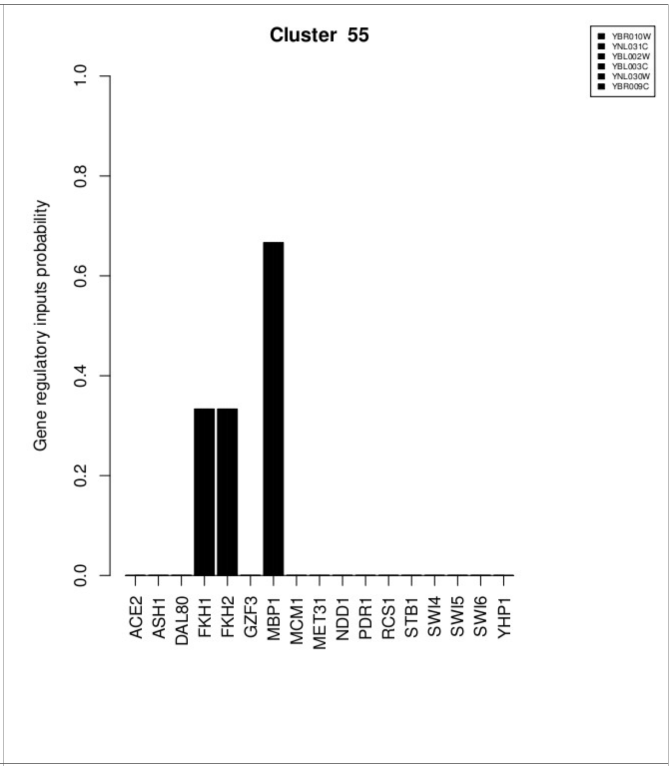
Cluster 55
YBR010W
YNL031C
YBL002W
YBL003C
YNL030W
YBR009C
Gene regulatory inputs probability
1.0
0.8
0.6
0.4
0.2
0.0
ACE2
ASH1
DAL80
FKH1
FKH2
GZF3
MBP1
MCM1
MET31
NDD1
PDR1
RCS1
STB1
SWI4
SWI5
SWI6
YHP1

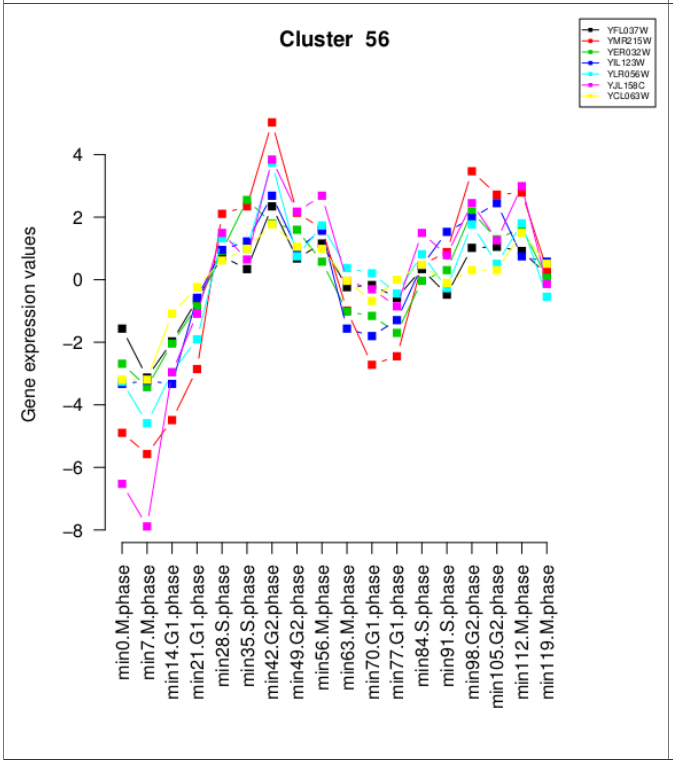
Cluster 56
YFL037W
YMR215W
YER032W
YIL123W
YLR056W
YJL158C
YCL063W
Gene expression values
4
2
0
−2
−4
−6
−8
min0.M.phase
min7.M.phase
min14.G1.phase
min21.G1.phase
min28.S.phase
min35.S.phase
min42.G2.phase
min49.G2.phase
min56.M.phase
min63.M.phase
min70.G1.phase
min77.G1.phase
min84.S.phase
min91.S.phase
min98.G2.phase
min105.G2.phase
min112.M.phase
min119.M.phase

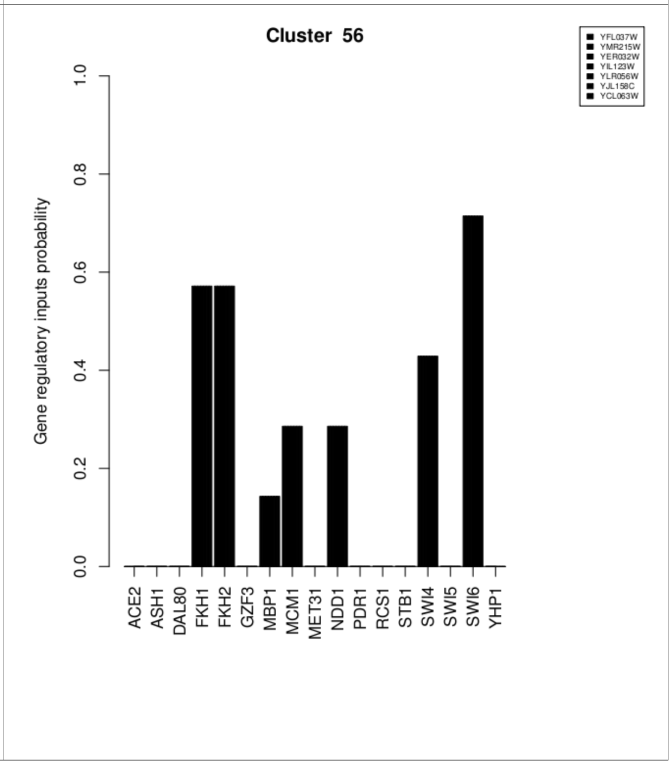
Cluster 56
YFL037W
YMR215W
YER032W
YIL123W
YLR056W
YJL158C
YCL063W
Gene regulatory inputs probability
1.0
0.8
0.6
0.4
0.2
0.0
ACE2
ASH1
DAL80
FKH1
FKH2
GZF3
MBP1
MCM1
MET31
NDD1
PDR1
RCS1
STB1
SWI4
SWI5
SWI6
YHP1

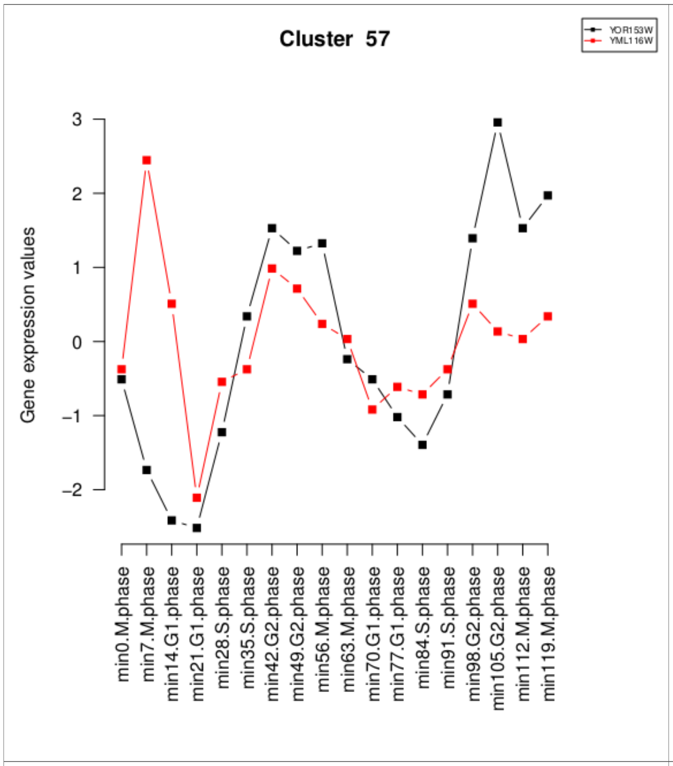

Cluster 57
YOR153W
YML116W
Gene expression values
3
2
1
0
−1
−2
min0.M.phase
min7.M.phase
min14.G1.phase
min21.G1.phase
min28.S.phase
min35.S.phase
min42.G2.phase
min49.G2.phase
min56.M.phase
min63.M.phase
min70.G1.phase
min77.G1.phase
min84.S.phase
min91.S.phase
min98.G2.phase
min105.G2.phase
min112.M.phase
min119.M.phase

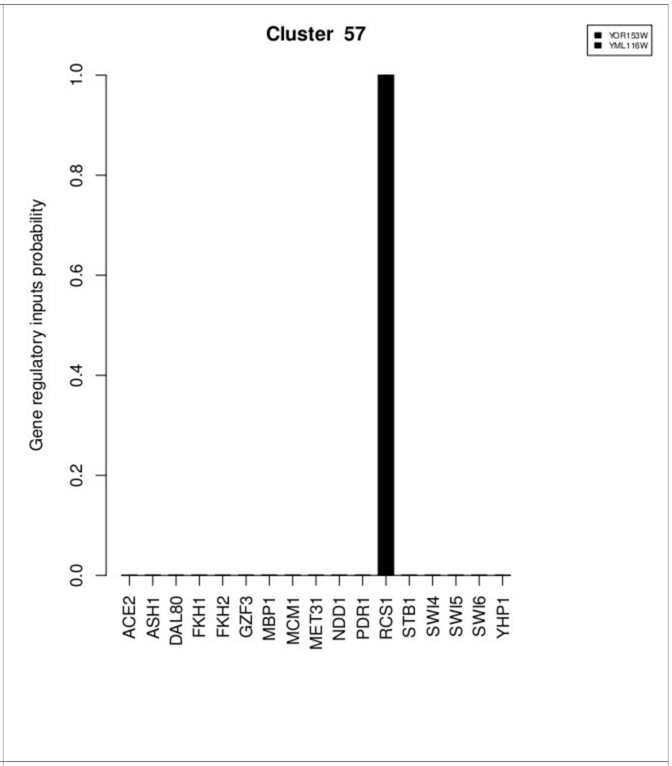

Cluster 57
YOR153W
YML116W
Gene regulatory inputs probability
1.0
0.8
0.6
0.4
0.2
0.0
ACE2
ASH1
DAL80
FKH1
FKH2
GZF3
MBP1
MCM1
MET31
NDD1
PDR1
RCS1
STB1
SWI4
SWI5
SWI6
YHP1

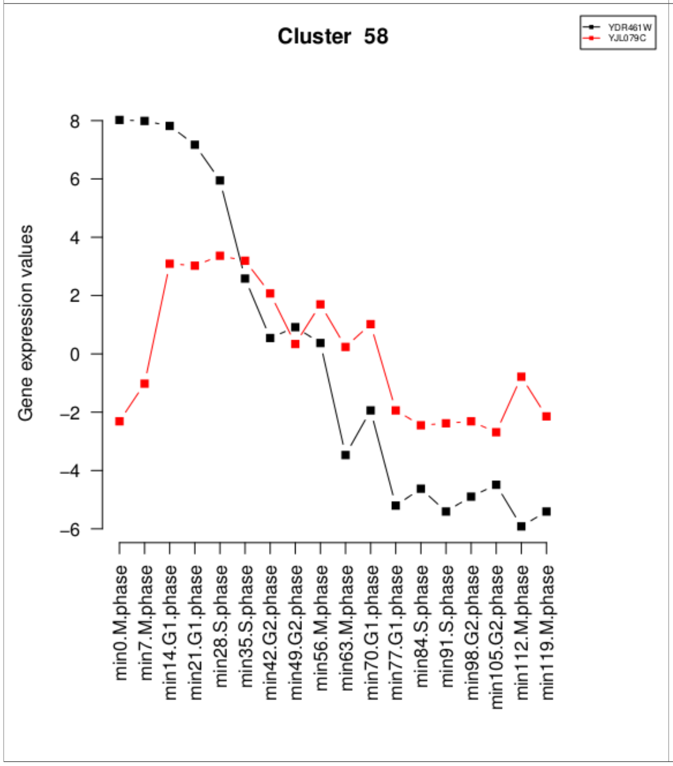

Cluster 58
YDR461W
YJL079C
Gene expression values
8
6
4
2
0
−2
−4
−6
min0.M.phase
min7.M.phase
min14.G1.phase
min21.G1.phase
min28.S.phase
min35.S.phase
min42.G2.phase
min49.G2.phase
min56.M.phase
min63.M.phase
min70.G1.phase
min77.G1.phase
min84.S.phase
min91.S.phase
min98.G2.phase
min105.G2.phase
min112.M.phase
min119.M.phase

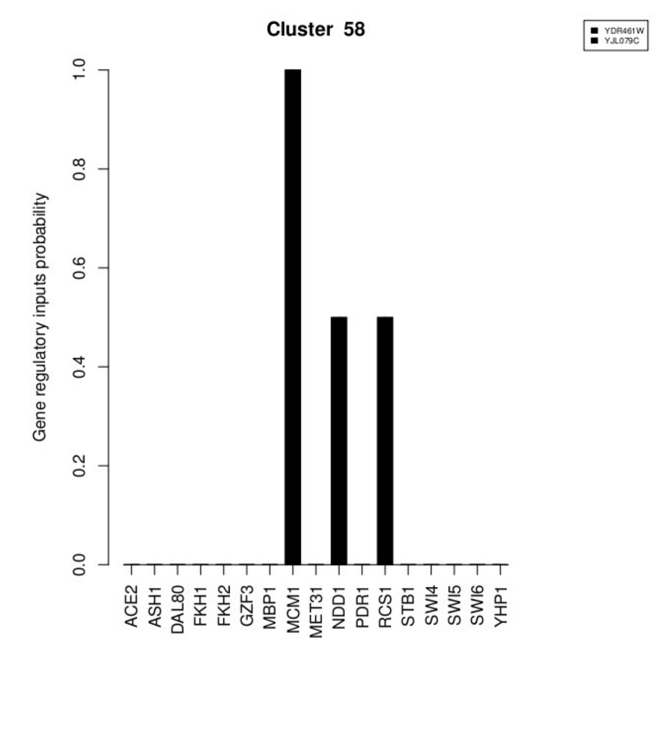

Cluster 58
YDR461W
YJL079C
Gene regulatory inputs probability
1.0
0.8
0.6
0.4
0.2
0.0
ACE2
ASH1
DAL80
FKH1
FKH2
GZF3
MBP1
MCM1
MET31
NDD1
PDR1
RCS1
STB1
SWI4
SWI5
SWI6
YHP1

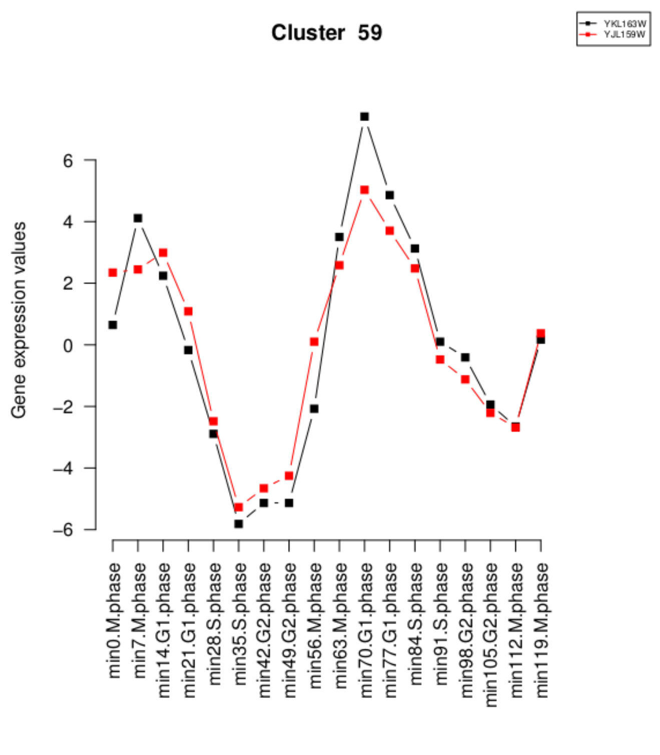
Cluster 59
YKL163W
YJL159W
Gene expression values
6
4
2
0
−2
−4
−6
min0.M.phase
min7.M.phase
min14.G1.phase
min21.G1.phase
min28.S.phase
min35.S.phase
min42.G2.phase
min49.G2.phase
min56.M.phase
min63.M.phase
min70.G1.phase
min77.G1.phase
min84.S.phase
min91.S.phase
min98.G2.phase
min105.G2.phase
min112.M.phase
min119.M.phase

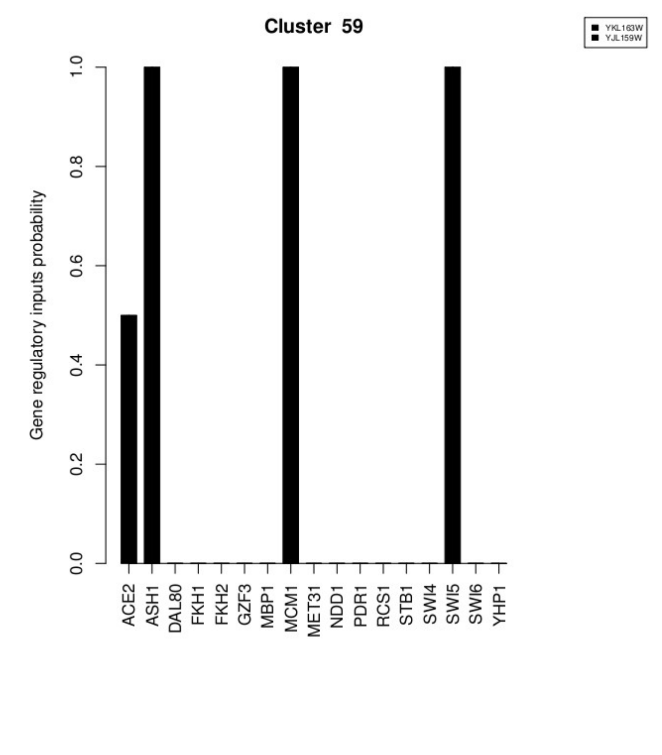
Cluster 59
YKL163W
YJL159W
Gene regulatory inputs probability
0.0
0.2
0.4
0.6
0.8
1.0
ACE2
ASH1
DAL80
FKH1
FKH2
GZF3
MBP1
MCM1
MET31
NDD1
PDR1
RCS1
STB1
SWI4
SWI5
SWI6
YHP1

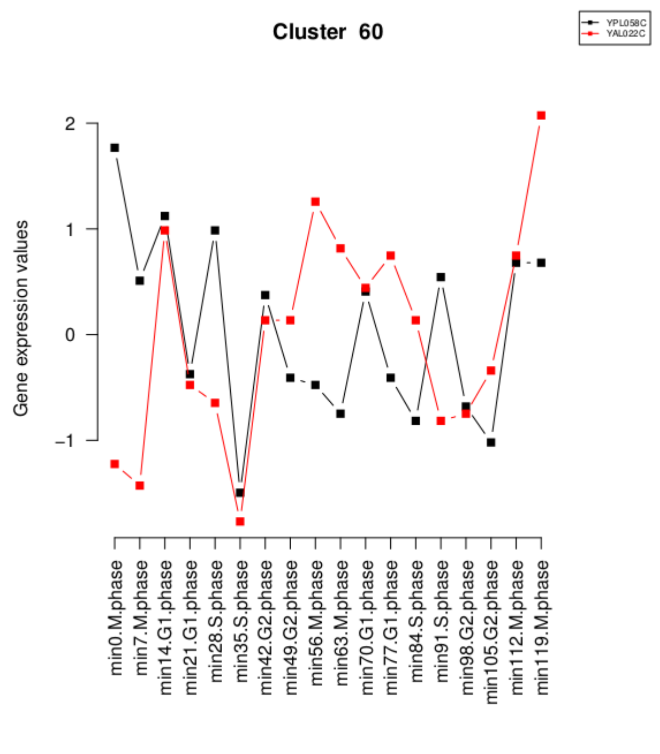
Cluster 60
YPL058C
YAL022C
Gene expression values
2
1
0
−1
min0.M.phase
min7.M.phase
min14.G1.phase
min21.G1.phase
min28.S.phase
min35.S.phase
min42.G2.phase
min49.G2.phase
min56.M.phase
min63.M.phase
min70.G1.phase
min77.G1.phase
min84.S.phase
min91.S.phase
min98.G2.phase
min105.G2.phase
min112.M.phase
min119.M.phase

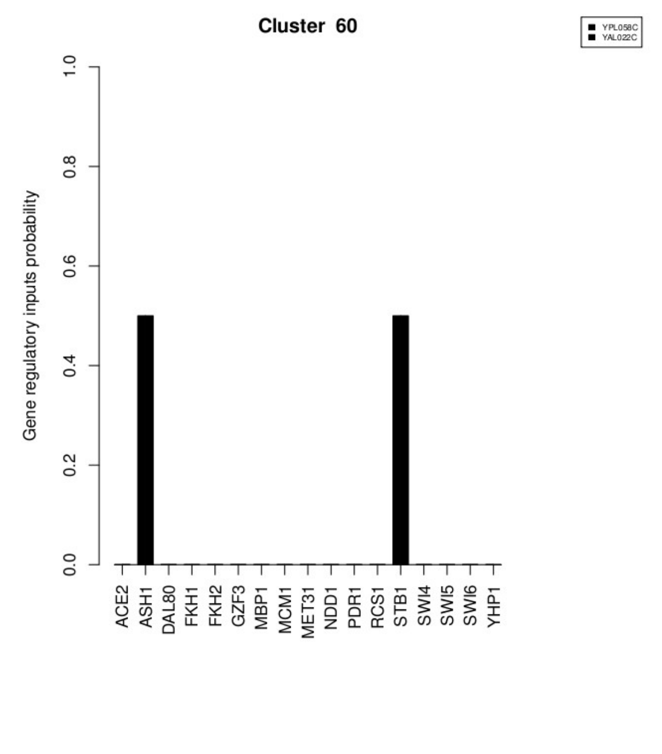
Cluster 60
YPL058C
YAL022C
Gene regulatory inputs probability
0.0
0.2
0.4
0.6
0.8
1.0
ACE2
ASH1
DAL80
FKH1
FKH2
GZF3
MBP1
MCM1
MET31
NDD1
PDR1
RCS1
STB1
SWI4
SWI5
SWI6
YHP1

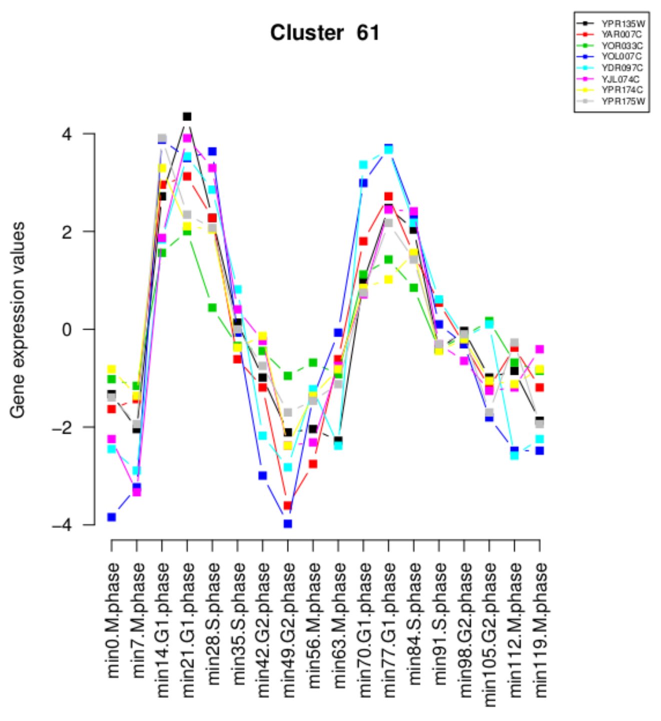
Cluster 61
YPR135W
YAR007C
YOR033C
YOL007C
YDR097C
YJL074C
YPR174C
YPR175W
Gene expression values
4
2
0
−2
−4
min0.M.phase
min7.M.phase
min14.G1.phase
min21.G1.phase
min28.S.phase
min35.S.phase
min42.G2.phase
min49.G2.phase
min56.M.phase
min63.M.phase
min70.G1.phase
min77.G1.phase
min84.S.phase
min91.S.phase
min98.G2.phase
min105.G2.phase
min112.M.phase
min119.M.phase

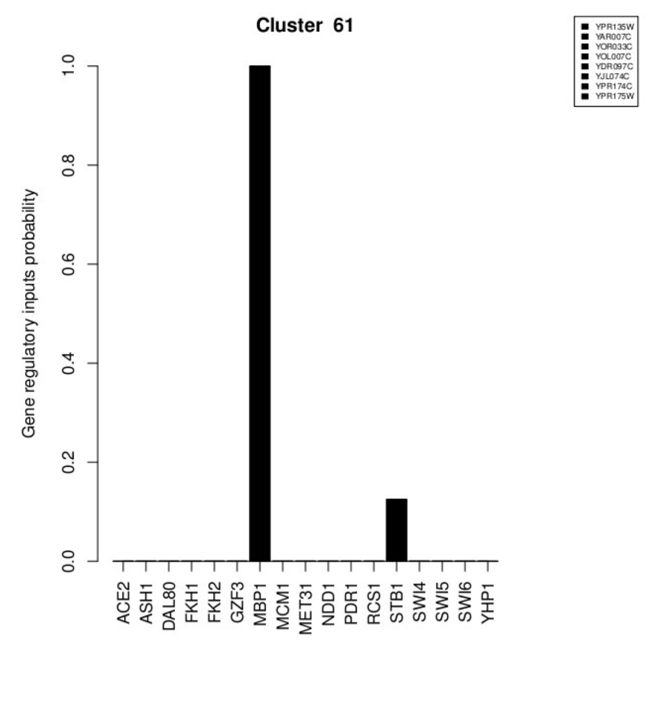
Cluster 61
YPR135W
YAR007C
YOR033C
YOL007C
YDR097C
YJL074C
YPR174C
YPR175W
Gene regulatory inputs probability
0.0
0.2
0.4
0.6
0.8
1.0
ACE2
ASH1
DAL80
FKH1
FKH2
GZF3
MBP1
MCM1
MET31
NDD1
PDR1
RCS1
STB1
SWI4
SWI5
SWI6
YHP1

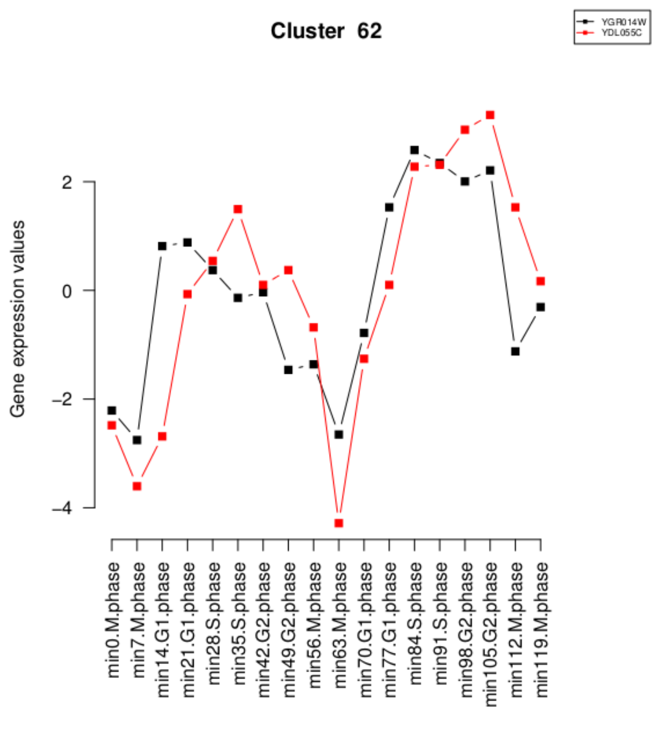
Cluster 62
YGR014W
YDL055C
Gene expression values
2
0
−2
−4
min0.M.phase
min7.M.phase
min14.G1.phase
min21.G1.phase
min28.S.phase
min35.S.phase
min42.G2.phase
min49.G2.phase
min56.M.phase
min63.M.phase
min70.G1.phase
min77.G1.phase
min84.S.phase
min91.S.phase
min98.G2.phase
min105.G2.phase
min112.M.phase
min119.M.phase

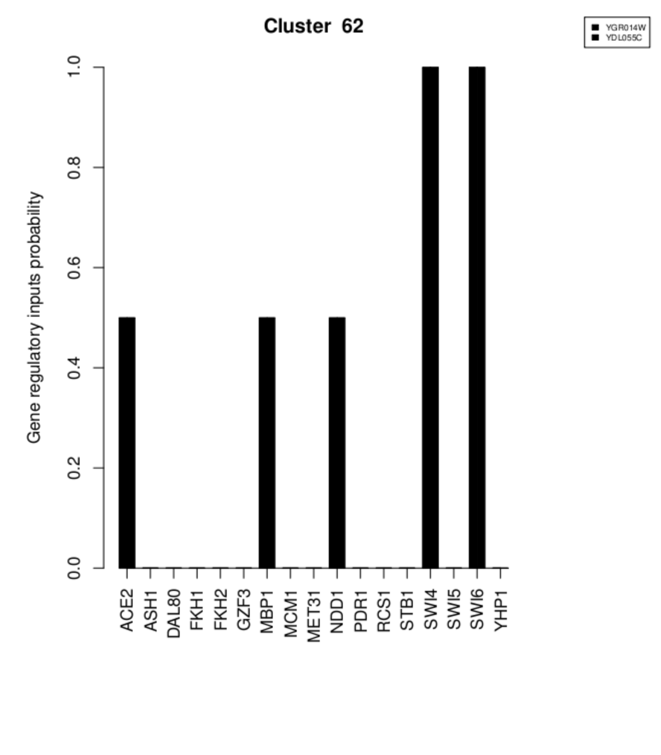
Cluster 62
YGR014W
YDL055C
Gene regulatory inputs probability
0.0
0.2
0.4
0.6
0.8
1.0
ACE2
ASH1
DAL80
FKH1
FKH2
GZF3
MBP1
MCM1
MET31
NDD1
PDR1
RCS1
STB1
SWI4
SWI5
SWI6
YHP1

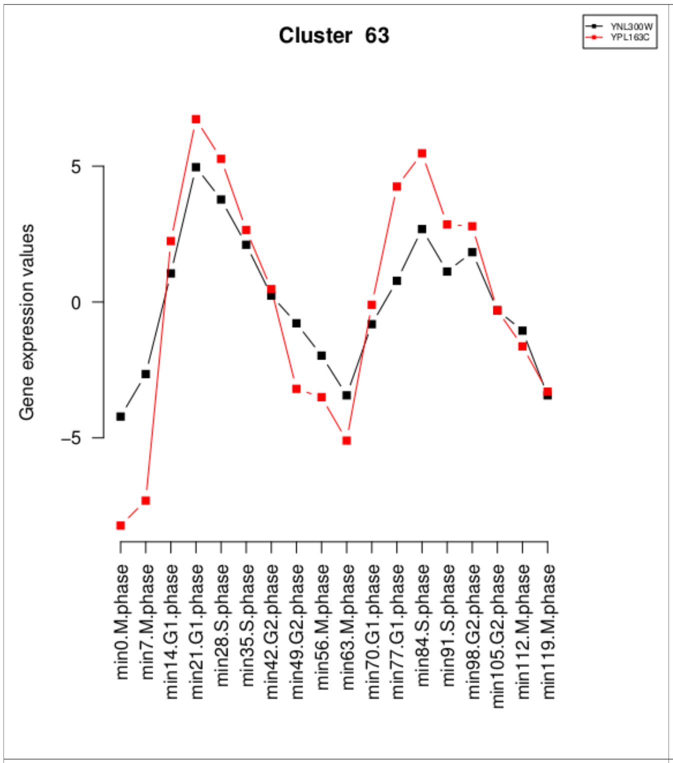
Cluster 63
YNL300W
YPL163C
Gene expression values
5
0
−5
min0.M.phase
min7.M.phase
min14.G1.phase
min21.G1.phase
min28.S.phase
min35.S.phase
min42.G2.phase
min49.G2.phase
min56.M.phase
min63.M.phase
min70.G1.phase
min77.G1.phase
min84.S.phase
min91.S.phase
min98.G2.phase
min105.G2.phase
min112.M.phase
min119.M.phase

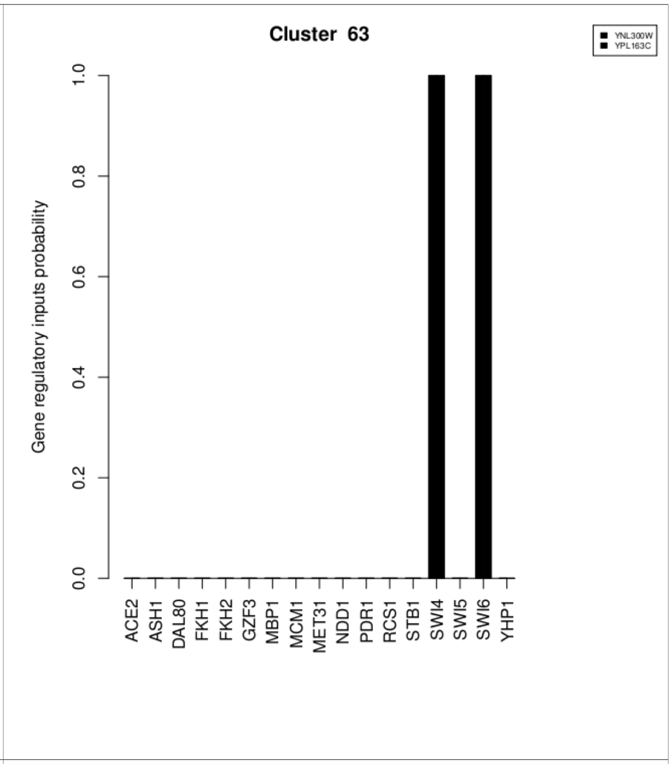
Cluster 63
YNL300W
YPL163C
Gene regulatory inputs probability
0.0
0.2
0.4
0.6
0.8
1.0
ACE2
ASH1
DAL80
FKH1
FKH2
GZF3
MBP1
MCM1
MET31
NDD1
PDR1
RCS1
STB1
SWI4
SWI5
SWI6
YHP1

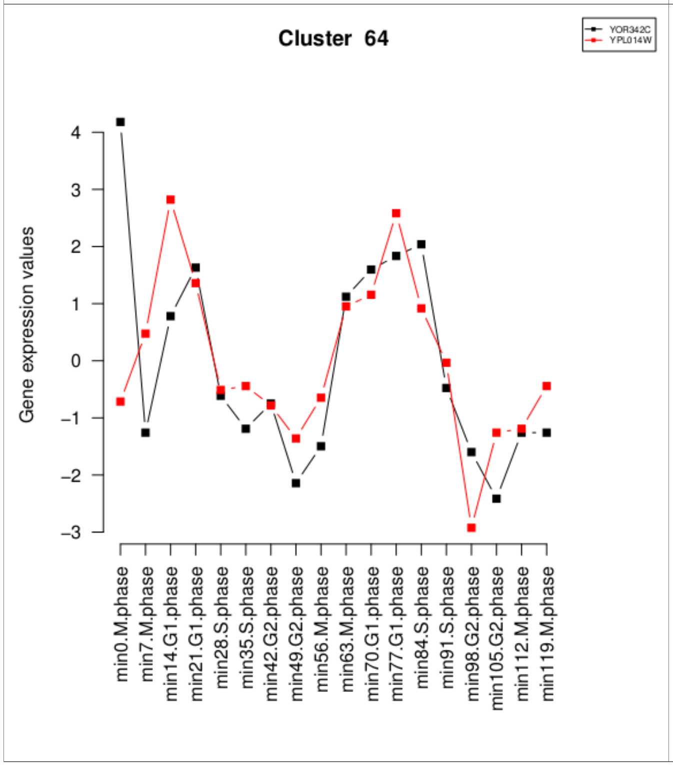
Cluster 64
YOR342C
YPL014W
Gene expression values
4
3
2
1
0
−1
−2
−3
min0.M.phase
min7.M.phase
min14.G1.phase
min21.G1.phase
min28.S.phase
min35.S.phase
min42.G2.phase
min49.G2.phase
min56.M.phase
min63.M.phase
min70.G1.phase
min77.G1.phase
min84.S.phase
min91.S.phase
min98.G2.phase
min105.G2.phase
min112.M.phase
min119.M.phase

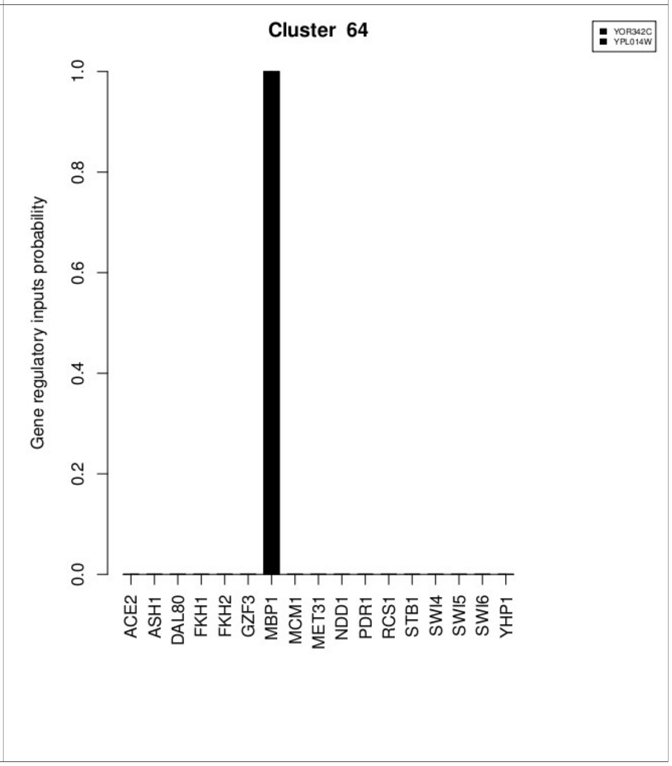
Cluster 64
YOR342C
YPL014W
Gene regulatory inputs probability
0.0
0.2
0.4
0.6
0.8
1.0
ACE2
ASH1
DAL80
FKH1
FKH2
GZF3
MBP1
MCM1
MET31
NDD1
PDR1
RCS1
STB1
SWI4
SWI5
SWI6
YHP1

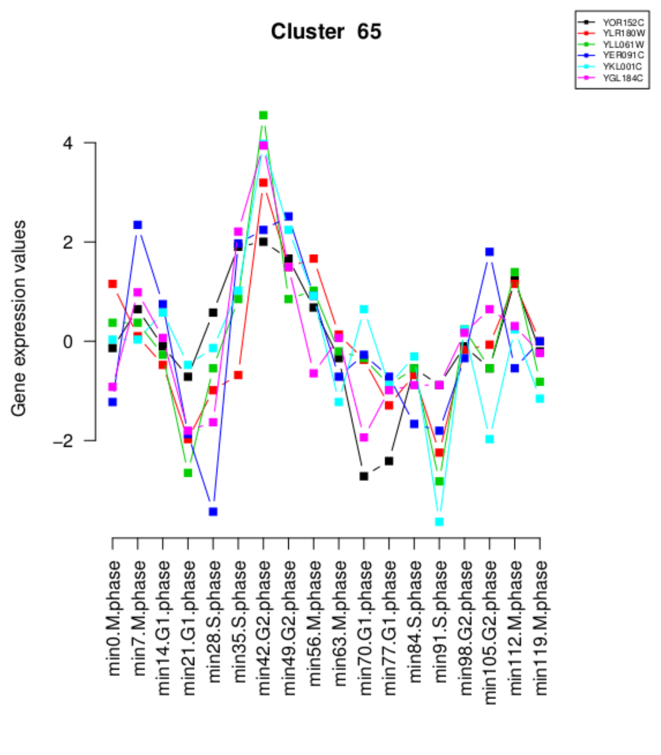
Cluster 65
YOR152C
YLR180W
YLL061W
YER091C
YKL001C
YGL184C
Gene expression values
4
2
0
−2
min0.M.phase
min7.M.phase
min14.G1.phase
min21.G1.phase
min28.S.phase
min35.S.phase
min42.G2.phase
min49.G2.phase
min56.M.phase
min63.M.phase
min70.G1.phase
min77.G1.phase
min84.S.phase
min91.S.phase
min98.G2.phase
min105.G2.phase
min112.M.phase
min119.M.phase

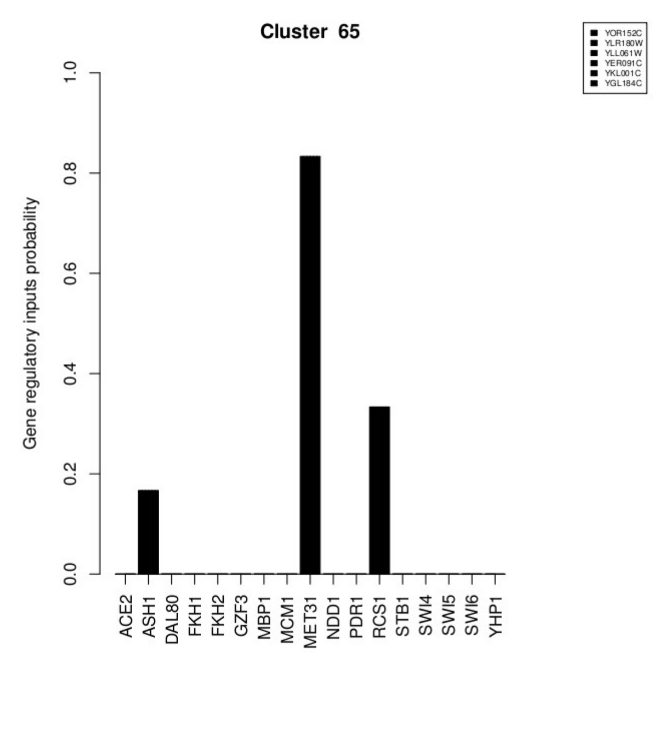
Cluster 65
YOR152C
YLR180W
YLL061W
YER091C
YKL001C
YGL184C
Gene regulatory inputs probability
1.0
0.8
0.6
0.4
0.2
0.0
ACE2
ASH1
DAL80
FKH1
FKH2
GZF3
MBP1
MCM1
MET31
NDD1
PDR1
RCS1
STB1
SWI4
SWI5
SWI6
YHP1

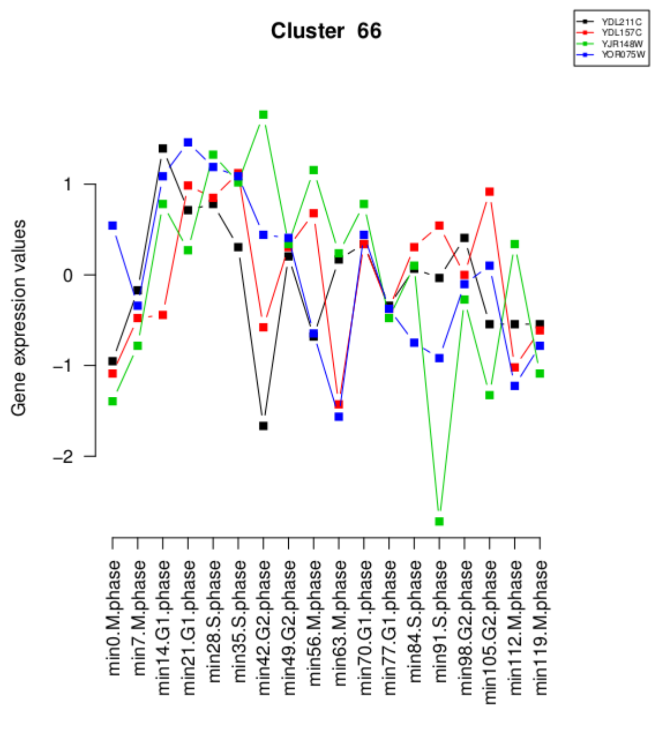
Cluster 66
YDL211C
YDL157C
YJR148W
YOR075W
Gene expression values
1
0
−1
−2
min0.M.phase
min7.M.phase
min14.G1.phase
min21.G1.phase
min28.S.phase
min35.S.phase
min42.G2.phase
min49.G2.phase
min56.M.phase
min63.M.phase
min70.G1.phase
min77.G1.phase
min84.S.phase
min91.S.phase
min98.G2.phase
min105.G2.phase
min112.M.phase
min119.M.phase

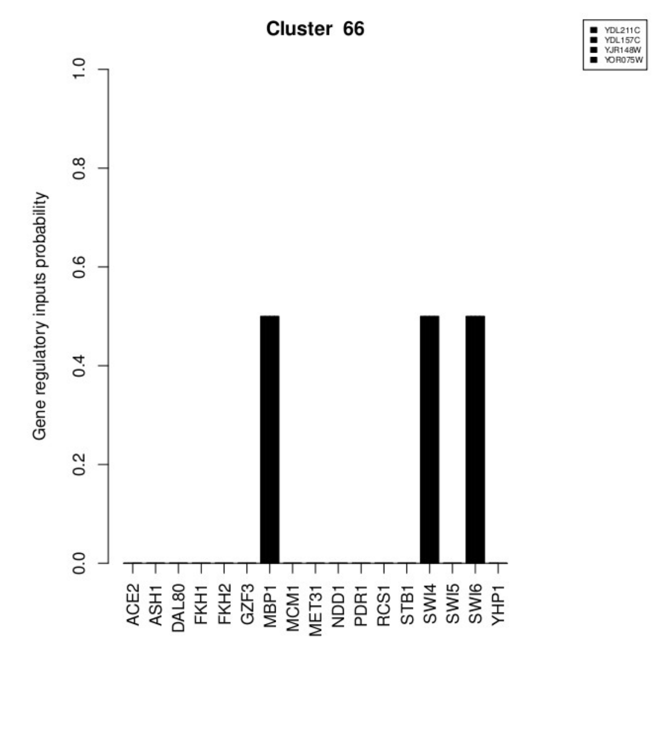
Cluster 66
YDL211C
YDL157C
YJR148W
YOR075W
Gene regulatory inputs probability
1.0
0.8
0.6
0.4
0.2
0.0
ACE2
ASH1
DAL80
FKH1
FKH2
GZF3
MBP1
MCM1
MET31
NDD1
PDR1
RCS1
STB1
SWI4
SWI5
SWI6
YHP1

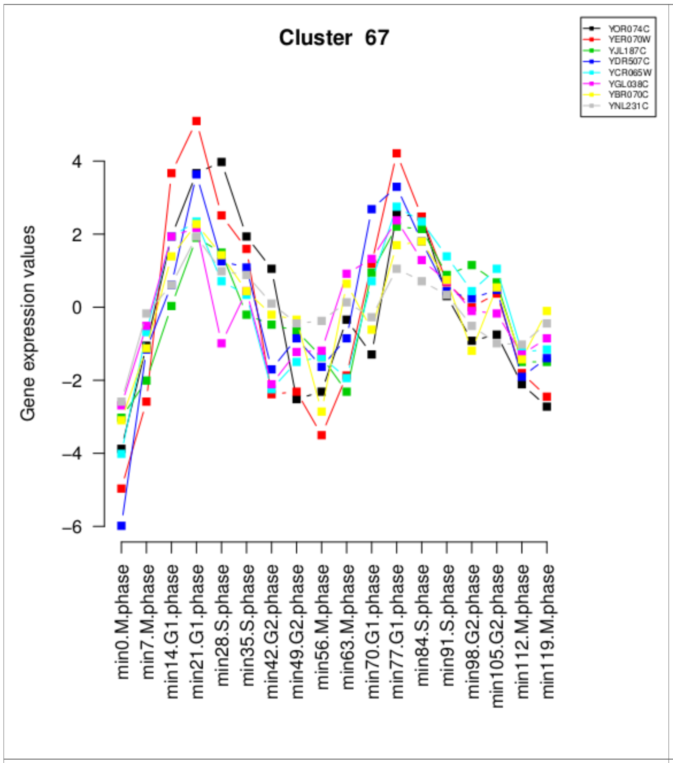
Cluster 67
YOR074C
YER070W
YJL187C
YDR507C
YCR065W
YGL038C
YBR070C
YNL231C
Gene expression values
4
2
0
−2
−4
−6
min0.M.phase
min7.M.phase
min14.G1.phase
min21.G1.phase
min28.S.phase
min35.S.phase
min42.G2.phase
min49.G2.phase
min56.M.phase
min63.M.phase
min70.G1.phase
min77.G1.phase
min84.S.phase
min91.S.phase
min98.G2.phase
min105.G2.phase
min112.M.phase
min119.M.phase

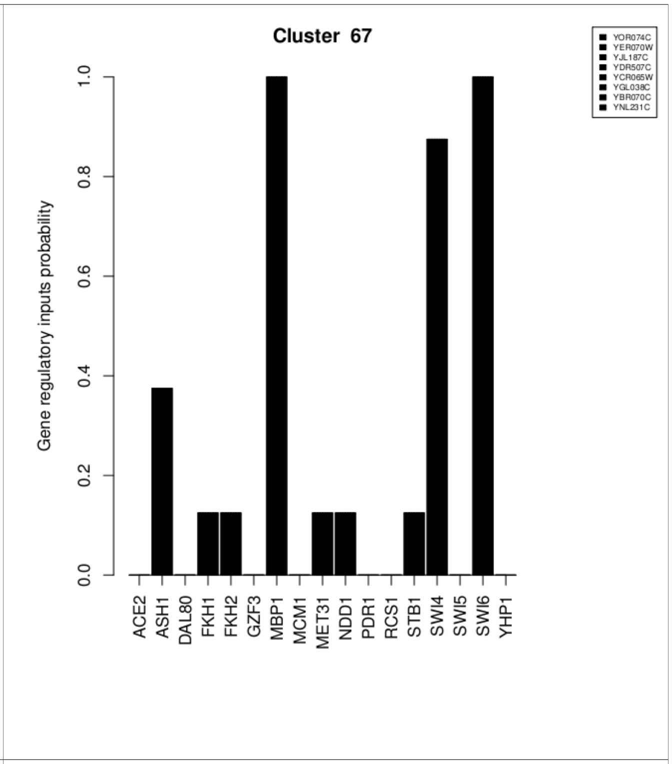
Cluster 67
YOR074C
YER070W
YJL187C
YDR507C
YCR065W
YGL038C
YBR070C
YNL231C
Gene regulatory inputs probability
1.0
0.8
0.6
0.4
0.2
0.0
ACE2
ASH1
DAL80
FKH1
FKH2
GZF3
MBP1
MCM1
MET31
NDD1
PDR1
RCS1
STB1
SWI4
SWI5
SWI6
YHP1

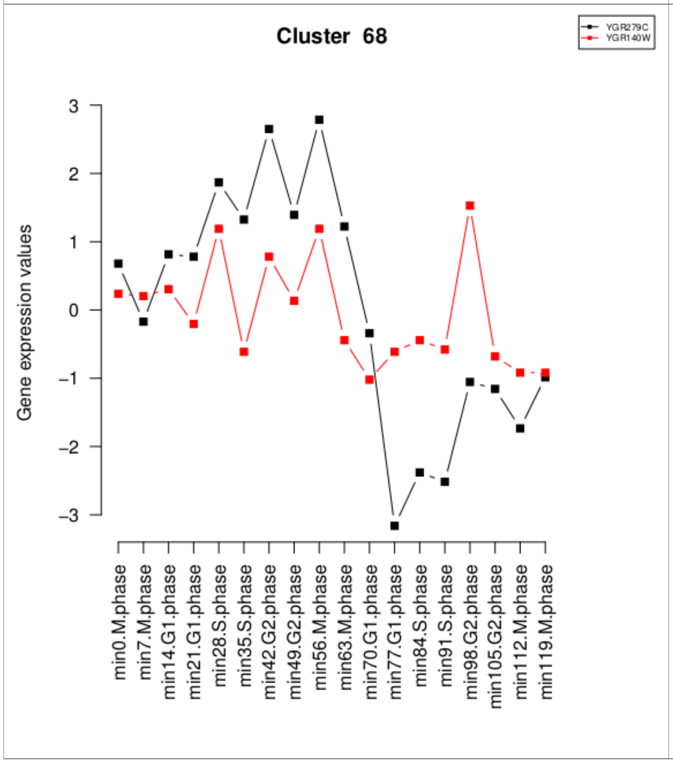
Cluster 68
YGR279C
YGR140W
Gene expression values
3
2
1
0
−1
−2
−3
min0.M.phase
min7.M.phase
min14.G1.phase
min21.G1.phase
min28.S.phase
min35.S.phase
min42.G2.phase
min49.G2.phase
min56.M.phase
min63.M.phase
min70.G1.phase
min77.G1.phase
min84.S.phase
min91.S.phase
min98.G2.phase
min105.G2.phase
min112.M.phase
min119.M.phase

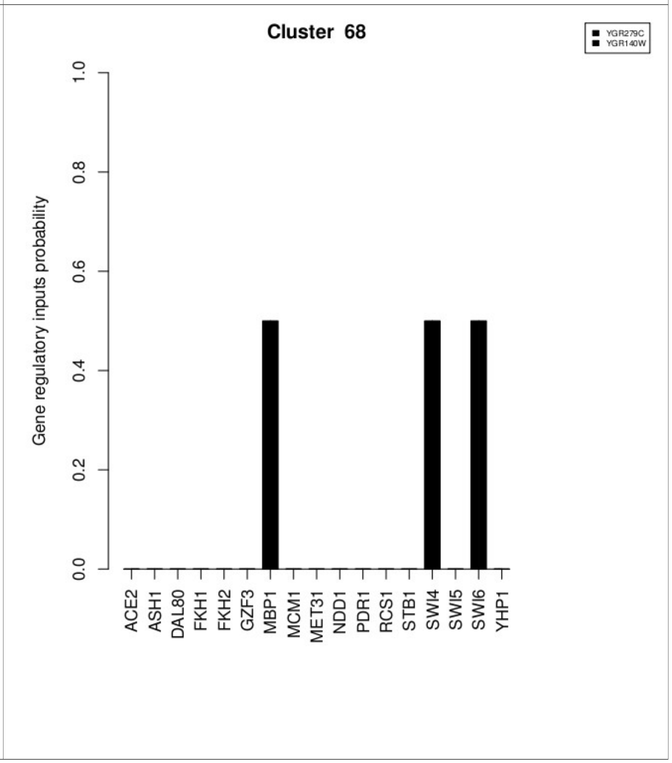
Cluster 68
YGR279C
YGR140W
Gene regulatory inputs probability
1.0
0.8
0.6
0.4
0.2
0.0
ACE2
ASH1
DAL80
FKH1
FKH2
GZF3
MBP1
MCM1
MET31
NDD1
PDR1
RCS1
STB1
SWI4
SWI5
SWI6
YHP1

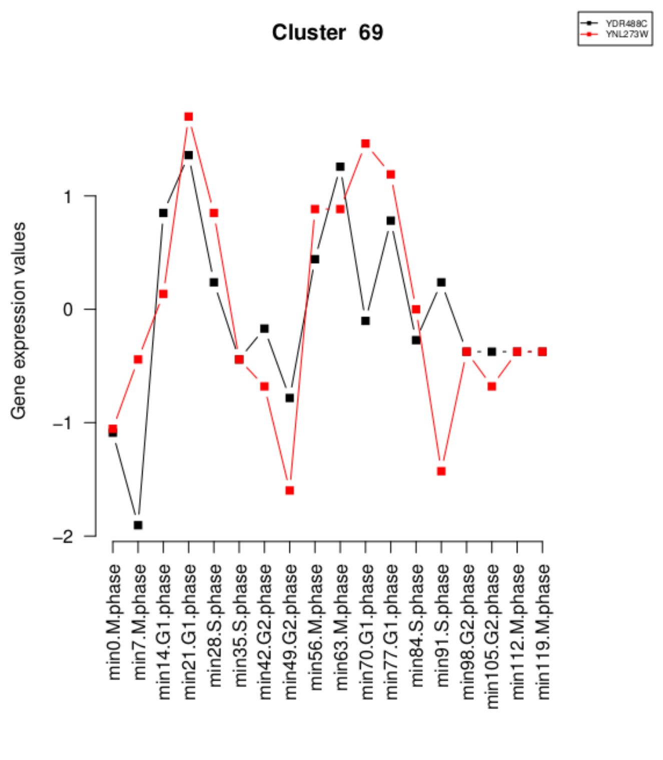
Cluster 69
YDR488C
YNL273W
Gene expression values
1
0
−1
−2
min0.M.phase
min7.M.phase
min14.G1.phase
min21.G1.phase
min28.S.phase
min35.S.phase
min42.G2.phase
min49.G2.phase
min56.M.phase
min63.M.phase
min70.G1.phase
min77.G1.phase
min84.S.phase
min91.S.phase
min98.G2.phase
min105.G2.phase
min112.M.phase
min119.M.phase

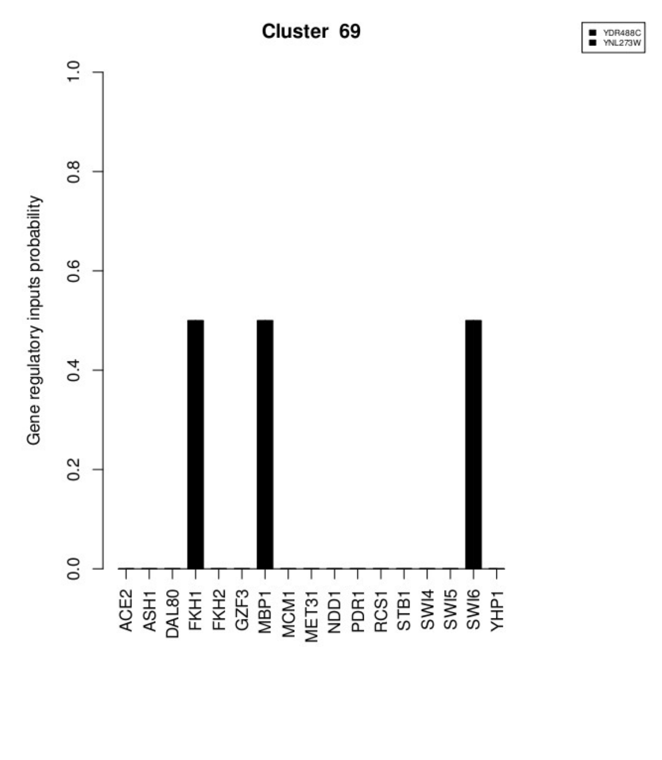
Cluster 69
YDR488C
YNL273W
Gene regulatory inputs probability
1.0
0.8
0.6
0.4
0.2
0.0
ACE2
ASH1
DAL80
FKH1
FKH2
GZF3
MBP1
MCM1
MET31
NDD1
PDR1
RCS1
STB1
SWI4
SWI5
SWI6
YHP1

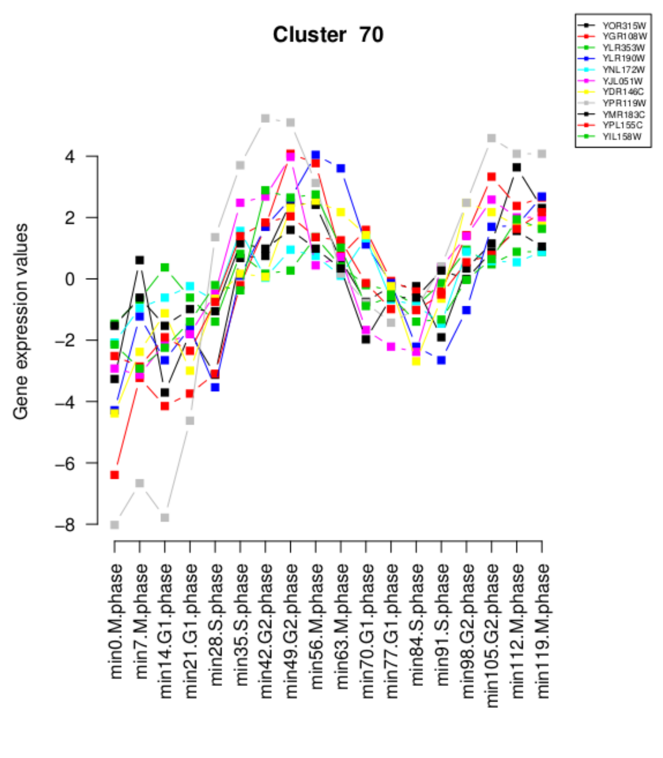
Cluster 70
YOR315W
YGR108W
YLR353W
YLR190W
YNL172W
YJL051W
YDR146C
YPR119W
YMR183C
YPL155C
YIL158W
Gene expression values
4
2
0
−2
−4
−6
−8
min0.M.phase
min7.M.phase
min14.G1.phase
min21.G1.phase
min28.S.phase
min35.S.phase
min42.G2.phase
min49.G2.phase
min56.M.phase
min63.M.phase
min70.G1.phase
min77.G1.phase
min84.S.phase
min91.S.phase
min98.G2.phase
min105.G2.phase
min112.M.phase
min119.M.phase

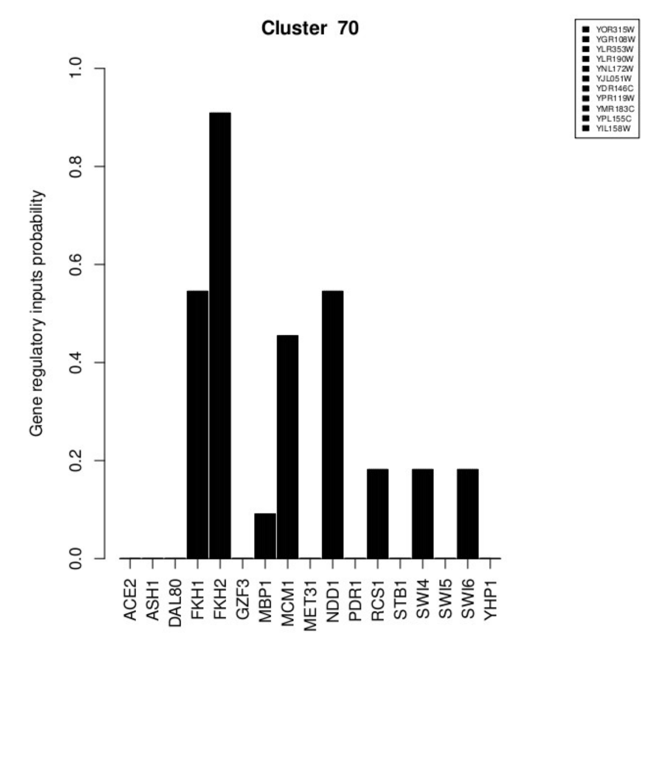
Cluster 70
YOR315W
YGR108W
YLR353W
YLR190W
YNL172W
YJL051W
YDR146C
YPR119W
YMR183C
YPL155C
YIL158W
Gene regulatory inputs probability
1.0
0.8
0.6
0.4
0.2
0.0
ACE2
ASH1
DAL80
FKH1
FKH2
GZF3
MBP1
MCM1
MET31
NDD1
PDR1
RCS1
STB1
SWI4
SWI5
SWI6
YHP1

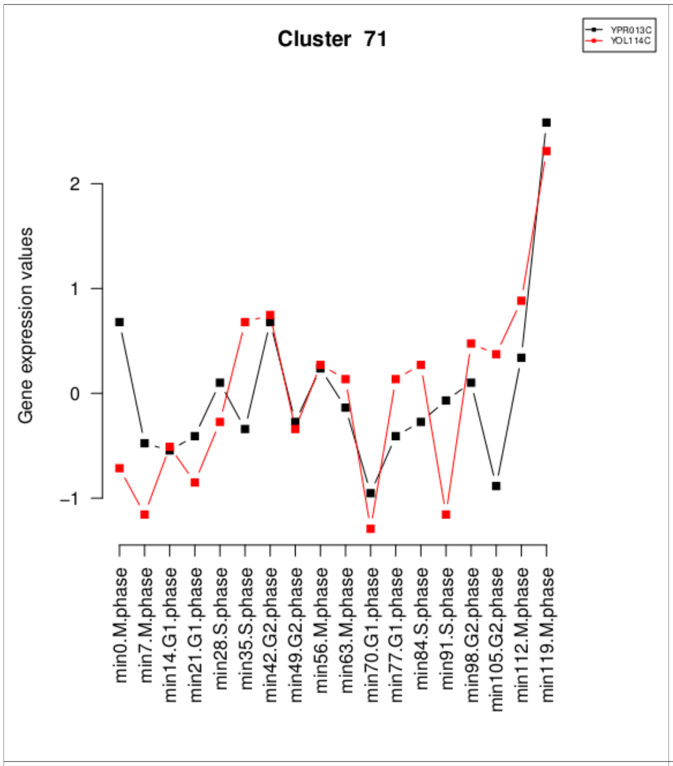
Cluster 71
YPR013C
YOL114C
Gene expression values
min0.M.phase
min7.M.phase
min14.G1.phase
min21.G1.phase
min28.S.phase
min35.S.phase
min42.G2.phase
min49.G2.phase
min56.M.phase
min63.M.phase
min70.G1.phase
min77.G1.phase
min84.S.phase
min91.S.phase
min98.G2.phase
min105.G2.phase
min112.M.phase
min119.M.phase

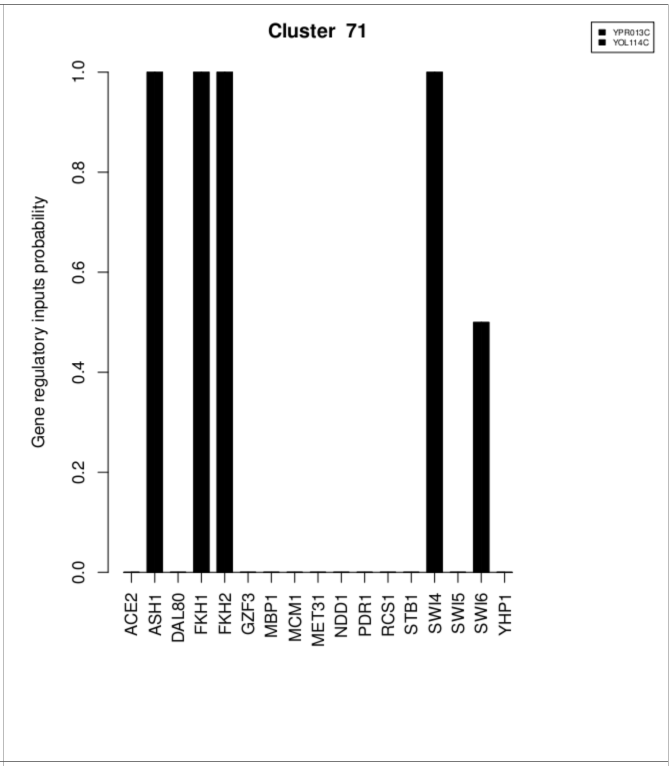
Cluster 71
YPR013C
YOL114C
Gene regulatory inputs probability
ACE2
ASH1
DAL80
FKH1
FKH2
GZF3
MBP1
MCM1
MET31
NDD1
PDR1
RCS1
STB1
SWI4
SWI5
SWI6
YHP1

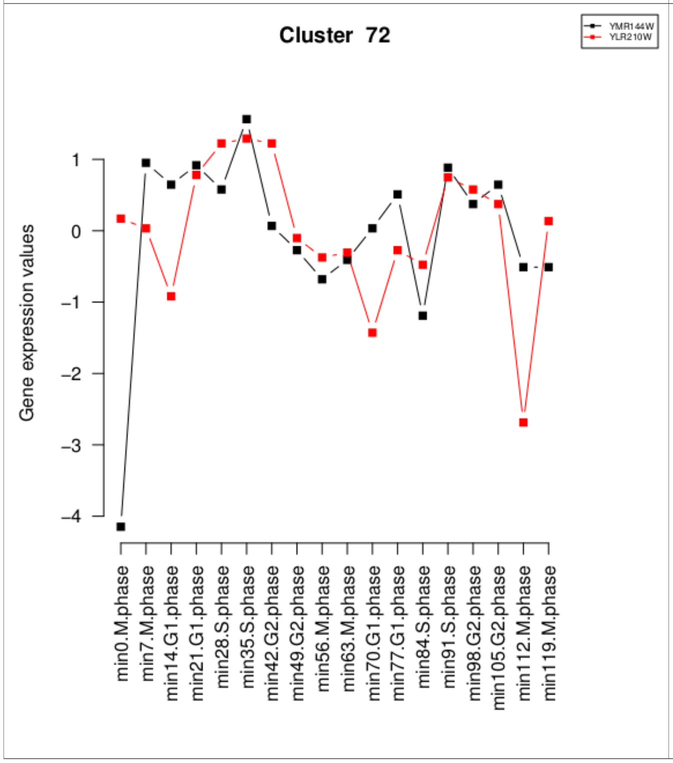
Cluster 72
YMR144W
YLR210W
Gene expression values
min0.M.phase
min7.M.phase
min14.G1.phase
min21.G1.phase
min28.S.phase
min35.S.phase
min42.G2.phase
min49.G2.phase
min56.M.phase
min63.M.phase
min70.G1.phase
min77.G1.phase
min84.S.phase
min91.S.phase
min98.G2.phase
min105.G2.phase
min112.M.phase
min119.M.phase

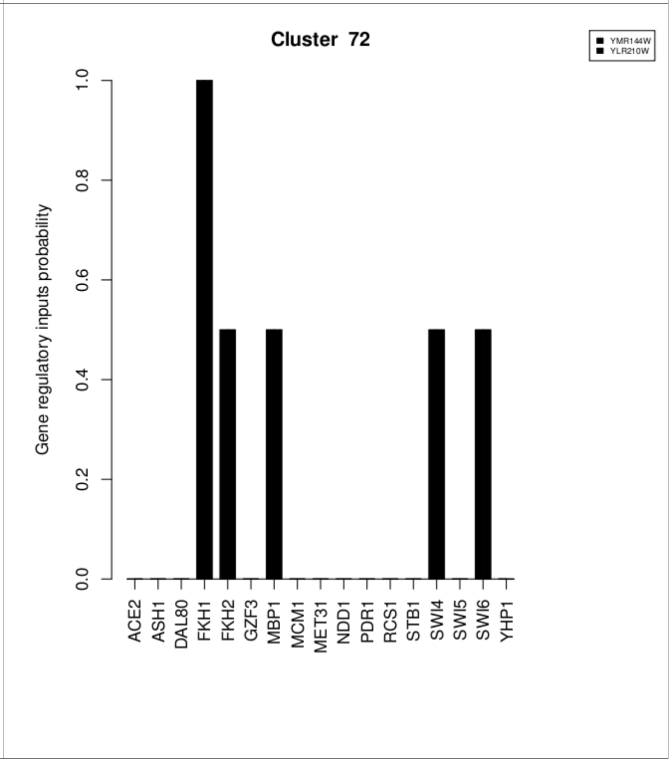
Cluster 72
YMR144W
YLR210W
Gene regulatory inputs probability
ACE2
ASH1
DAL80
FKH1
FKH2
GZF3
MBP1
MCM1
MET31
NDD1
PDR1
RCS1
STB1
SWI4
SWI5
SWI6
YHP1

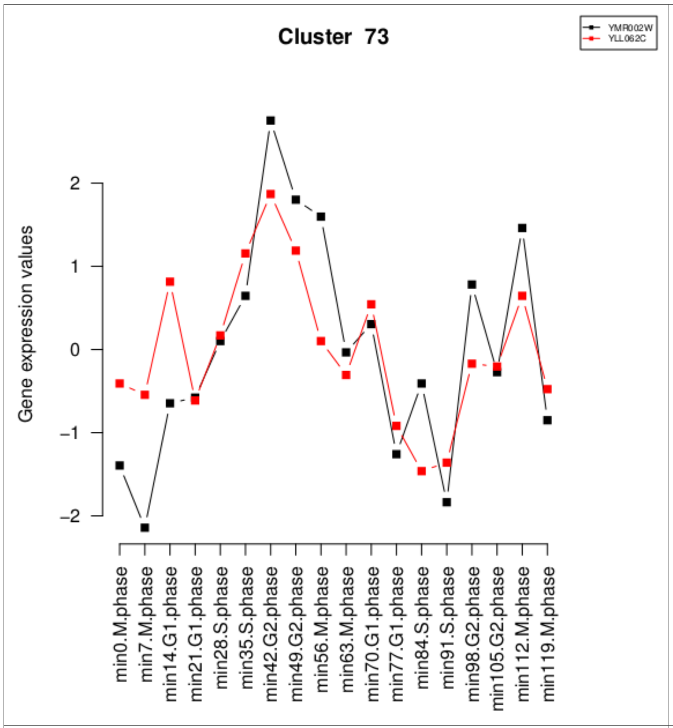
Cluster 73
YMR002W
YLL062C
Gene expression values
2
1
0
−1
−2
min0.M.phase
min7.M.phase
min14.G1.phase
min21.G1.phase
min28.S.phase
min35.S.phase
min42.G2.phase
min49.G2.phase
min56.M.phase
min63.M.phase
min70.G1.phase
min77.G1.phase
min84.S.phase
min91.S.phase
min98.G2.phase
min105.G2.phase
min112.M.phase
min119.M.phase

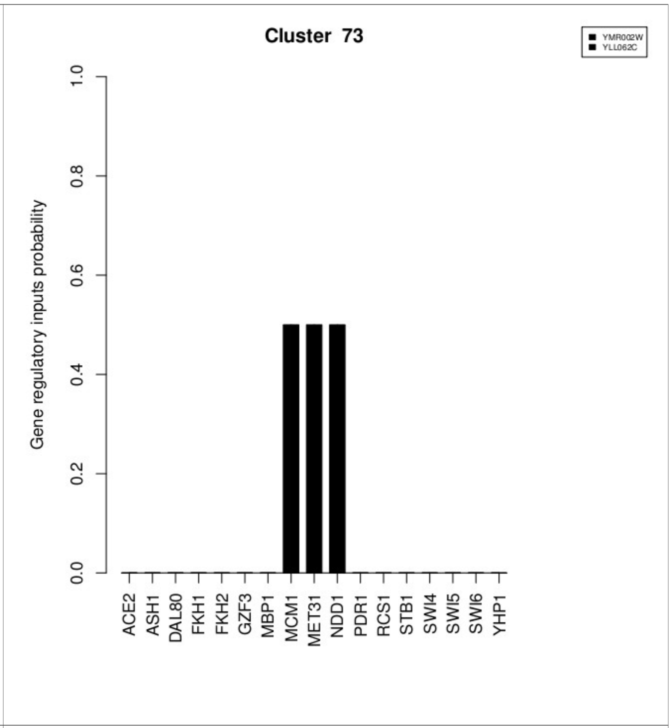
Cluster 73
YMR002W
YLL062C
Gene regulatory inputs probability
0.0
0.2
0.4
0.6
0.8
1.0
ACE2
ASH1
DAL80
FKH1
FKH2
GZF3
MBP1
MCM1
MET31
NDD1
PDR1
RCS1
STB1
SWI4
SWI5
SWI6
YHP1

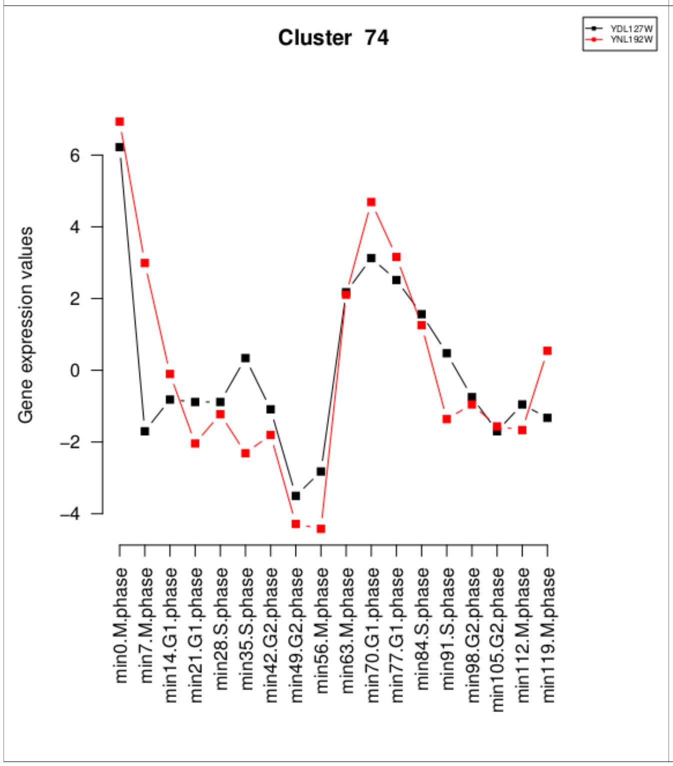
Cluster 74
YDL127W
YNL192W
Gene expression values
6
4
2
0
−2
−4
min0.M.phase
min7.M.phase
min14.G1.phase
min21.G1.phase
min28.S.phase
min35.S.phase
min42.G2.phase
min49.G2.phase
min56.M.phase
min63.M.phase
min70.G1.phase
min77.G1.phase
min84.S.phase
min91.S.phase
min98.G2.phase
min105.G2.phase
min112.M.phase
min119.M.phase

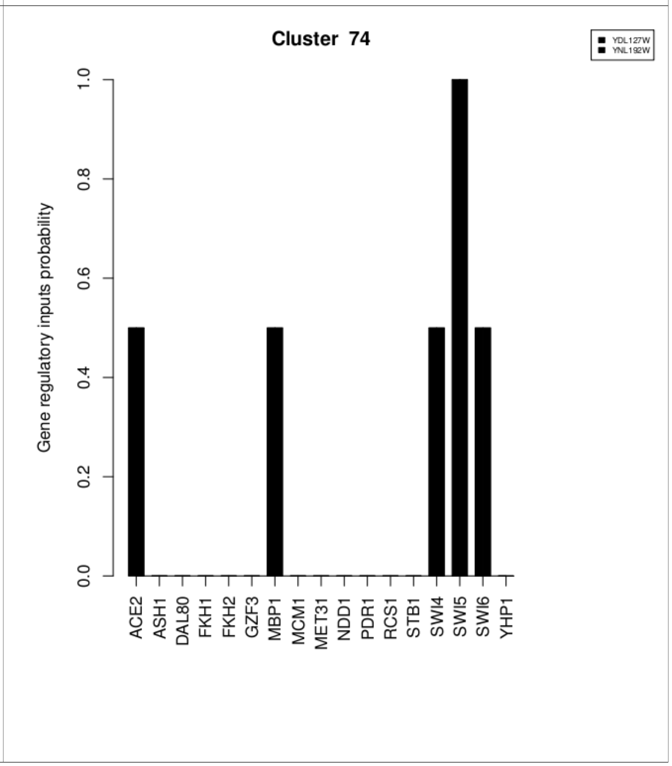
Cluster 74
YDL127W
YNL192W
Gene regulatory inputs probability
0.0
0.2
0.4
0.6
0.8
1.0
ACE2
ASH1
DAL80
FKH1
FKH2
GZF3
MBP1
MCM1
MET31
NDD1
PDR1
RCS1
STB1
SWI4
SWI5
SWI6
YHP1

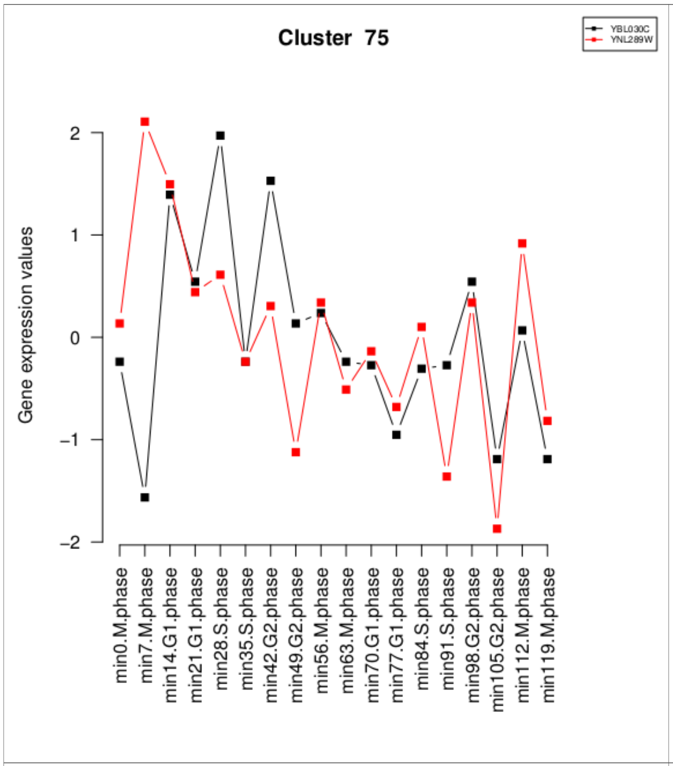
Cluster 75
YBL030C
YNL289W
Gene expression values
2
1
0
−1
−2
min0.M.phase
min7.M.phase
min14.G1.phase
min21.G1.phase
min28.S.phase
min35.S.phase
min42.G2.phase
min49.G2.phase
min56.M.phase
min63.M.phase
min70.G1.phase
min77.G1.phase
min84.S.phase
min91.S.phase
min98.G2.phase
min105.G2.phase
min112.M.phase
min119.M.phase

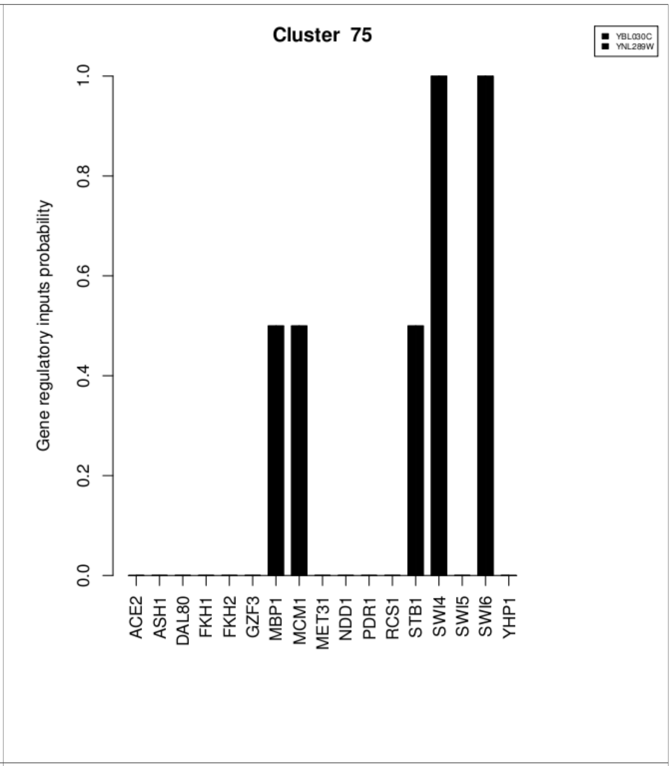
Cluster 75
YBL030C
YNL289W
Gene regulatory inputs probability
1.0
0.8
0.6
0.4
0.2
0.0
ACE2
ASH1
DAL80
FKH1
FKH2
GZF3
MBP1
MCM1
MET31
NDD1
PDR1
RCS1
STB1
SWI4
SWI5
SWI6
YHP1

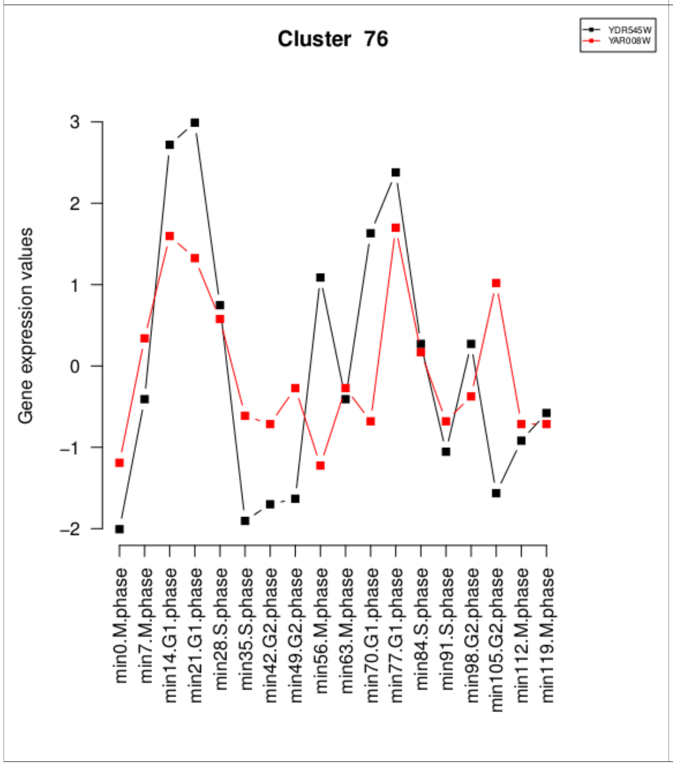
Cluster 76
YDR545W
YAR008W
Gene expression values
3
2
1
0
−1
−2
min0.M.phase
min7.M.phase
min14.G1.phase
min21.G1.phase
min28.S.phase
min35.S.phase
min42.G2.phase
min49.G2.phase
min56.M.phase
min63.M.phase
min70.G1.phase
min77.G1.phase
min84.S.phase
min91.S.phase
min98.G2.phase
min105.G2.phase
min112.M.phase
min119.M.phase

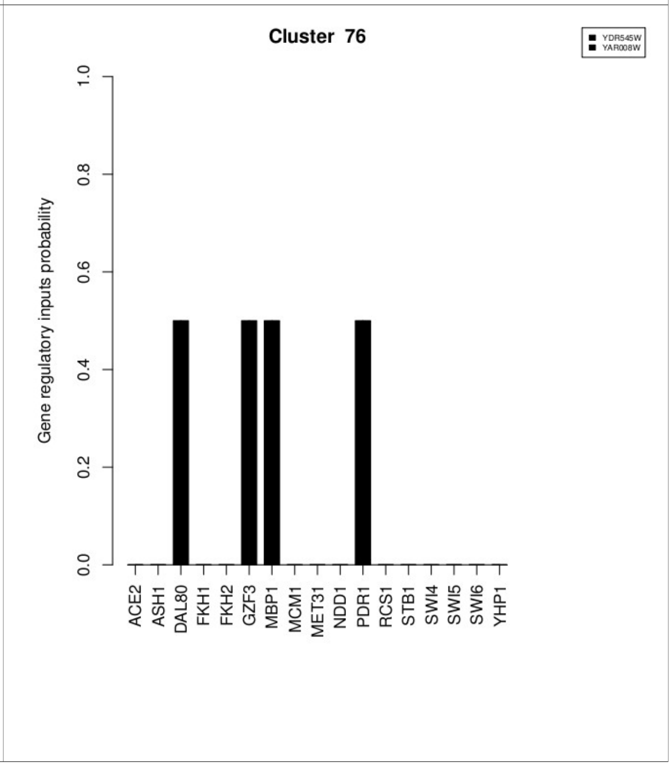
Cluster 76
YDR545W
YAR008W
Gene regulatory inputs probability
1.0
0.8
0.6
0.4
0.2
0.0
ACE2
ASH1
DAL80
FKH1
FKH2
GZF3
MBP1
MCM1
MET31
NDD1
PDR1
RCS1
STB1
SWI4
SWI5
SWI6
YHP1
