# Supplementary material for: Flexible model-based clustering of mixed binary and continuous data: application to genetic regulation and cancer
Source: Nucleic Acids Res. 2016 Dec 19;45(7):e53. doi: 10.1093/nar/gkw1270 (PMC5399749; doi:10.1093/nar/gkw1270)
Supplement: Supplementary Data [file gkw1270_supplementary_data.zip › nar-02952-met-n-2016-File012.doc]

| **Sample (AML patients)** | **Mutated genes*** | **Variably expressed genes**** |
| --- | --- | --- |
| TCGA-AB-2948-03 TCGA-AB-2853-03 TCGA-AB-2942-03  TCGA-AB-2909-03 TCGA-AB-2970-03 TCGA-AB-2825-03  TCGA-AB-3000-03 TCGA-AB-2985-03 TCGA-AB-2987-03  TCGA-AB-2910-03 TCGA-AB-2994-03 TCGA-AB-2812-03  TCGA-AB-2890-03 TCGA-AB-2980-03 TCGA-AB-2857-03  TCGA-AB-3008-03 TCGA-AB-2862-03 TCGA-AB-2943-03  TCGA-AB-2866-03 TCGA-AB-2904-03 TCGA-AB-2982-03  TCGA-AB-2835-03 TCGA-AB-2863-03 TCGA-AB-2859-03  TCGA-AB-2933-03 TCGA-AB-2879-03 TCGA-AB-3007-03  TCGA-AB-2837-03 TCGA-AB-2963-03 TCGA-AB-2860-03  TCGA-AB-2995-03 TCGA-AB-2869-03 TCGA-AB-2988-03  TCGA-AB-2944-03 TCGA-AB-2818-03 TCGA-AB-2916-03  TCGA-AB-2847-03 TCGA-AB-2931-03 TCGA-AB-2981-03  TCGA-AB-2956-03 TCGA-AB-2881-03 TCGA-AB-2816-03  TCGA-AB-2903-03 TCGA-AB-2833-03 TCGA-AB-2929-03  TCGA-AB-2977-03 TCGA-AB-2813-03 TCGA-AB-2950-03  TCGA-AB-2992-03 TCGA-AB-2927-03 TCGA-AB-2964-03  TCGA-AB-2954-03 TCGA-AB-2884-03 TCGA-AB-2901-03  TCGA-AB-2848-03 TCGA-AB-2998-03 TCGA-AB-2811-03  TCGA-AB-2815-03 TCGA-AB-2843-03 TCGA-AB-2959-03  TCGA-AB-2880-03 TCGA-AB-2971-03 TCGA-AB-2855-03  TCGA-AB-2973-03 TCGA-AB-2928-03 TCGA-AB-2967-03  TCGA-AB-2836-03 TCGA-AB-2821-03 TCGA-AB-2870-03  TCGA-AB-2873-03 TCGA-AB-2882-03 TCGA-AB-2839-03  TCGA-AB-2845-03 TCGA-AB-2840-03 TCGA-AB-2972-03  TCGA-AB-2832-03 TCGA-AB-2949-03 TCGA-AB-2937-03  TCGA-AB-2940-03 TCGA-AB-2895-03 TCGA-AB-2877-03  TCGA-AB-2856-03 TCGA-AB-2810-03 TCGA-AB-2849-03  TCGA-AB-2841-03 TCGA-AB-2965-03 TCGA-AB-2865-03  TCGA-AB-2911-03 TCGA-AB-2861-03 TCGA-AB-2806-03  TCGA-AB-2842-03 TCGA-AB-2930-03 TCGA-AB-2925-03  TCGA-AB-2823-03 TCGA-AB-3002-03 TCGA-AB-2887-03  TCGA-AB-3011-03 TCGA-AB-2914-03 TCGA-AB-2918-03  TCGA-AB-2991-03 TCGA-AB-2969-03 TCGA-AB-3006-03  TCGA-AB-2896-03 TCGA-AB-2913-03 TCGA-AB-2826-03  TCGA-AB-2838-03 TCGA-AB-2897-03 TCGA-AB-2814-03  TCGA-AB-2941-03 TCGA-AB-2891-03 TCGA-AB-2934-03  TCGA-AB-2851-03 TCGA-AB-2886-03 TCGA-AB-2805-03  TCGA-AB-2986-03 TCGA-AB-2828-03 TCGA-AB-2803-03  TCGA-AB-2990-03 TCGA-AB-2844-03 TCGA-AB-2819-03  TCGA-AB-2820-03 TCGA-AB-2978-03 TCGA-AB-2808-03  TCGA-AB-2921-03 TCGA-AB-2858-03 TCGA-AB-2983-03  TCGA-AB-2867-03 TCGA-AB-2999-03 TCGA-AB-2888-03  TCGA-AB-2955-03 TCGA-AB-2919-03 TCGA-AB-2996-03  TCGA-AB-2898-03 TCGA-AB-2846-03 TCGA-AB-2854-03  TCGA-AB-2935-03 TCGA-AB-2976-03 TCGA-AB-3001-03  TCGA-AB-2979-03 TCGA-AB-2966-03 TCGA-AB-2908-03  TCGA-AB-2932-03 TCGA-AB-2885-03 TCGA-AB-2872-03  TCGA-AB-2824-03 TCGA-AB-2868-03 TCGA-AB-2917-03  TCGA-AB-2899-03 TCGA-AB-3012-03 TCGA-AB-2938-03  TCGA-AB-2939-03 TCGA-AB-2952-03 TCGA-AB-2875-03  TCGA-AB-2924-03 TCGA-AB-2912-03 TCGA-AB-2874-03  TCGA-AB-2830-03 TCGA-AB-2993-03 TCGA-AB-2915-03  TCGA-AB-2936-03 TCGA-AB-2817-03 TCGA-AB-3009-03  TCGA-AB-2834-03 TCGA-AB-2807-03 TCGA-AB-2920-03  TCGA-AB-3005-03 TCGA-AB-2900-03 TCGA-AB-2871-03  TCGA-AB-2984-03 TCGA-AB-2822-03 | FRYL BMPER  OR13H1 PHACTR1  PRPF8 GLTSCR1L  CACNA2D3 PDCD2L  SEMA4A ARAP2  TUBA3C GJB3  PPP1R3A MED12  RYR3 FLG  CACNA1E KMT2C  PLEKHH1 PSME4  MTMR8 CROCC  ZC3H18 TNC  LRRC37B P2RY2  NUP98 APOB  ATP1B4 CADPS  ZNF687 TTBK1  FCGBP NMUR2  GATA2 DIS3  DDX41 SAXO2  NRXN3 CALR  MEFV E2F8  GRM3 PRAMEF2  SCARB1 SCN1A  DNMT3B PICALM  FOXP1 GSTK1  DOCK2 SMG1  DCLK1 CUL1  SETBP1 MUC16  PKHD1L1 RNF213  THRAP3 SPEN  C17ORF97 ASXL1  EPPK1 NF1  UNC5B GBP4  ADGRG4 NSD1  ZBTB33 CACNA1B  ATP10B COL12A1  TRPM3 BCR  DCHS2 GRID1  OR11H12 PTPRT  CDK11B TFG  FAM57B EZH2  LRBA TMEM255B  ETV6 SUZ12  DDR2 GAS6  DNAH9 ADGRG7  HECW1 PLCE1  HNRNPK ABL1  TOP3B ELL  CD74 RAD21  GIGYF2 BRINP3  CSMD3 TTN  CSMD1 STAG2  MUC5B PHF6  KCNA4 MLLT10  STRIP2 KRAS  MECOM KIT  KDM3B SMC1A  BSN SMC3  KDM6A KMT2A  PHIP U2AF1  DLC1 PTPN11  RIMS1 RUNX1T1  KDR MYH11  ILDR1 WT1  TET1 CBFB  WAC NRAS  TCEAL6 TP53  SCAF8 CEBPA  SEMA3A TET2  CADM2 RARA  GRIK2 PML  GRIK4 IDH1  GALNT18 IDH2  SI RUNX1  CNTNAP4 DNMT3A  CMYA5 FLT3  MAP2 NPM1 | UBE2V1 AHSP SH3BP4 GLT1D1 MYCT1 FAM127A ZFY NPR3 CSMD1 RNF217 GABRE CYorf15B  JPH1 BAALC C20orf54 DOCK1 EPCAM ELN LIFR MYOF LUM EPB42 UTY CT45A5  ASS1 CCNA1 GPR12 HOXB8 KIAA0087 HMGA2 DUSP27 PTPN20B AREG VENTX ZNF711 FERMT1  LHX6 TM4SF1 CYP1B1 NPTX2 PDK4 UNC13B NAPSB GPR126 SNCAIP CD200 TOM1L1 VCAM1  SLC28A3 SLCO5A1 NLRP2 FBLN1 C5orf23 DTNA RFPL1S DEFA4 MECOM CLEC7A HTR1F THNSL2  RETN LOC100101938 NRP1 PRLR HBM SEMA3C PTGER3 ITGB3 VGLL3 PPARGC1A EPX UGGT2  IRX5 LTBP1 FGD5 MEIS1 HBB HDC IRX1 FBN2 ANO7 HOXB3 LDLRAD3 MYCL1  IRX3 SLC24A3 PHACTR3 TRPM4 NKX2-3 TMSB4Y SLITRK4 ABO CLDN10 MSR1 MAFB VCAN  SLITRK5 CTSG CLC EPHB2 S100A12 PF4 PPP1R9A TPSAB1 NUDT10 FCN1 MS4A2 DEFA1B  MEG3 CA1 COL5A1 PLCB4 MS4A3 SERPINA1 COL23A1 DKK2 CNNM1 PHKA1 S100A16 CPNE8  GYPA ANK1 CD109 CLEC9A MRC1 TMIGD2 GYPB TPSD1 HOXA7 IGLL1 LGSN C5orf20  CXCL12 LILRA6 HOXA6 LAMC1 DNTT CPNE7 PRRG1 LILRA5 HOXA5 PAX8 RXFP1 GLI2  LOC441666 LILRA3 HOXA4 SCUBE1 SNORD116-4 SIGLEC9 PRR16 LIN7A HOXA3 FLJ22536 NDST3 OPALIN  GSTM1 SYCP2L COBL CYorf15A DSC2 BEX1 APOC2 TUSC1 HOXA9 PROK2 NTRK1 MYCN  LOC284551 C5AR1 PTK2 ADAMTS2 NAV3 POU4F1 ITGA9 PKP2 STAB1 HPGDS MPEG1 AR  CD1D CHI3L1 BPI GPR173 TDRD9 GPC4 CD1E FBLN2 SCN9A VAT1L DDX3Y GPC6  CD1C CYP4F2 CLGN C17orf55 AOX2P DACH1 EVC MS4A4A COL3A1 MDFI SPAG6 CLTCL1  MYO18B ADAMTS1 PRKY PTGFR HK3 SHANK1 PTGDS ADAMTS3 VSTM1 H2AFY2 ANXA8L2 CD163  VWDE DHRS9 LPHN3 EIF1AY NEGR1 PTPRG VPREB1 SAGE1 SCARA3 CYP2S1 HTR7 PTPRD  TNNT1 GNG11 MTMR11 ADAMTS18 UMODL1 ZFP57 LILRB4 ADRA2C ZNF727 BCORL2 TSPAN7 PTRF  LILRB2 CDH9 HOXA11AS KRT8 CACNA2D3 ALOX15B NXF3 CDH2 SCN2A FAM38B SERPINB2 PTPRM  SERPINB10 CDH4 DNAJC12 HOXA11 CALN1 MYO7A CCL23 DPP10 PACSIN1 HOXA10 BMP3 SIGLEC1  HNMT IL31RA XIST PPBP ROBO1 C8orf79 SDK2 RAMP1 C3orf50 STOX2 ROBO4 C20orf200  HOXB9 PTH2R MS4A6A DSG2 HBG1 TEX15 HOXB2 CYYR1 HBA1 C2 MEFV RHAG  KCNK17 LPO HBA2 SLC8A3 TMEM189- AADAT HOXB6 PROM1 PBX1 C7 L3MBTL4 UGT2B11  HOXB7 WIT1 CYP7B1 GPR85 KDM5D ELANE HOXB4 APP CLEC4E EPB41L3 DLK1 TTTY15  HOXB5 C1QC CLEC4D MYEF2 TLR8 KIAA1462 PPARG C1QB CLEC4C NDN SCN3A PRSS21  COL4A5 C1QA ASGR2 DDIT4L PXDN SLC44A5 TSIX AZU1 C20orf103 LGALS2 CT45A1 FGF13  C10orf114 KIF17 MOSC2 PAWR CT45A3 APBA1 TCN1 WNT7B MOSC1 FAM171A1 MAMDC2 CCDC48  LOC654433 VLDLR MMP8 SLPI HBG2 PTPN14 KRT17 TBC1D3G MMP9 AIF1L DPPA4 ZNHIT2  CD14 UGT3A2 TGFBI LRP1 TIFAB LOC644172 SORT1 SPON1 MMP2 LRP6 IL1R2 KIAA1598  FOXC1 SCHIP1 ANXA8 CDA ST18 TMEM136 KRT18 GTSF1 CRLF2 PRRT4 PPAN-P2RY11 SECTM1  SEPP1 LAMB2 SYTL4 GSTM5 CPA3 PI15 NCRNA00185 ARHGEF10L CD34 COL1A2 FAT1 HPGD  MKRN3 KIAA1324L BGN EREG IGSF10 LTK SLC4A1 MARCO MYL4 COL1A1 THBS1 ZNF334  SHROOM4 CDC42BPA CLEC14A KIAA1217 PCBP3 ADCY2 COL2A1 CEACAM6 C7orf58 CDH11 TRH SPINK2  HTRA3 TACSTD2 RANBP17 TRIM71 TRO KCNE1L CLEC5A CEACAM8 IL1RL1 KIRREL ENPEP LTF  RPS4Y1 BMX DCN NLRP12 MN1 CLEC10A PRAME VNN3 SELENBP1 S100P THSD7A TMEM105  PTX4 VNN1 ZNF521 FCGR3B EVPL ACY3 ALAS2 IGFBP2 DLGAP2 LOXHD1 C10orf140 DEFB1  S100A9 SHD FAM110B USP9Y PRDM16 MXRA5 S100A8 IGFBP5 PRTN3 RUNX1T1 CES1 ARPP21  PKLR CCDC8 FCAR SIX3 PLSCR4 TMEM176B END6 GJA1 FPR1 ALDH1A1 MPO LOC728606  BEND4 GJA3 FPR2 S100B PRODH SORCS1 LOC399959 FLJ42875 TPSB2 KYNU IL17RE CHRDL1  CYP27A1 AQP9 PLBD1 CD300E OLL2A MSLN TMEM176A FAM171B TMED7-TICAM2 |
|  |  |  |

**Supplementary table S1.** Input data points and variables consisting of 170 samples, 154 mutated genes and 500 most variably expressed genes.

*	Genes which are mutated in at least 2 samples

** Genes which are variably expressed based on ranked coefficient of variation and standard deviation

**Supplementary table S2.** Genes that are significantly differentially expressed between clusters determined using the one-versus-all phenotype test in the ComparativeMarkerSelection(v10) tool from [1]. Genes with FDR (Benjamini-Hochberg corrected) less than 0.005 and with 10.0<score>10.0 were selected. Genes in red are up-regulated in the cluster whereas those in blue are down-regulated. Clusters highlighted in yellow are clusters which are associated with mutations in particular sets of genes, given in green along with their cluster mutation probability (>0.5) at the foot of the list.

| **1** | **2** | **3** | **4** | **5** | **6** | **7** | **8** | **9** | **10** | **11** | **12** | **13** | **14** | **15** | **16** | **17** | **18** |
| --- | --- | --- | --- | --- | --- | --- | --- | --- | --- | --- | --- | --- | --- | --- | --- | --- | --- |
| SLITRK5  DLK1  CDH2  FLJ42875  DDIT4L  ZNF727  GPR173  BEND4  PAWR  FAM127A  **ELL**  **(1.00)**  **KMT2A (1.00)** | C1QC  DKK2  MSR1  LGALS2  CD300E  C5AR1  C1QB  LILRB2  PTGFR  HTR7  FPR1  HNMT  LILRA5  SIGLEC9  MS4A4A  MEFV  HK3  LDLRAD3  ARHGEF10L  SERPINA1  MPEG1  MS4A6A  SIGLEC1  KYNU  CPNE8  DLK1  ZNHIT2  SNCAIP  ARPP21  COL2A1  NUDT10  NCRNA00185  BCORL2  TMSB4Y  TTTY15  EIF1AY  CYorf15A  THSD7A  CYorf15B  USP9Y  CYYR1  KDM5D  DDX3Y  DLGAP2  DSG2  KIRREL  ENPEP  MXRA5  VGLL3  FAT1  CDH11  CHRDL1  COL3A1  PXDN  COL1A2  NDN  DCN  BGN  APP  COL1A1  HBB  **NPM1**  **(1.0)** | CYP4F2  JPH1  CLEC4C  COBL  ADRA2C  NKX2-3  KRT17  BMX  MARCO  PRR16  LIN7A  GSTM1  PLBD1  CD14  ADAMTS3  MOSC2  AREG  RNF217  FBN2  MOSC1  **PHF6**  (1.00) | PRDM16  C10orf140  NKX2-3  FLJ42875  HOXB3  HOXB6  COL4A5  HOXB4  HOXA5  HOXA6  HOXB2  HOXB5  HOXA3  HOXA7  IGSF10  MEIS1  HOXA4  HOXA9  TOM1L1  CYP7B1  TRH  **DNMT3A**  **(0.66)**  **FLT3**  **(0.66)**  **NPM1**  **(1.00)** | HOXA3  HK3  HOXA4  MAFB  HOXA7  HOXA5  HOXA6  CD300E  LILRA6  CDA  SERPINA1  HOXA10  CCL23  SIGLEC9  TMEM105  CHRDL1  APP  KIRREL  **FLT3**  **(0.60)**  **NPM1**  **(0.73)** | COL4A5  DNAJC12  IGSF10  CLEC4C  CD1E  SPAG6  TACSTD2  ASS1  IL31RA  NPTX2  DDIT4L  DLGAP2  PRR16  TSIX  LGALS2  CYP4F2  CEACAM6  S100A8  PRLR  S100A9  MYOF  BPI  **FLT3**  **(1.00)** | CCNA1  LOC399959  THSD7A  **NSD1**  **(0.75)**  **FLT3**  **(0.75)** | RUNX1T1  TSPAN7  MPO  RFPL1S  EVC  HPGDS  SLCO5A1  PRRT4  POU4F1  SHANK1  SLC24A3  LPO  PTX4  DNTT  C20orf54  TRIM71  ZNF711  SEMA3C  NEGR1  DPP10  KIAA0087  DOCK1  ABO  C3orf50  HOXB4  HOXB5  HTR1F  PHACTR3  HOXA5  IL31RA  HOXB3  NKX2-3  HOXB6  DTNA  MEIS1  ZNF334  HOXA3  FLJ42875  CPNE8  PPARGC1A  HOXA7  HOXA6  **RUNX1T1**  **(1.00)**  **RUNX1**  **(1.00)** | MSLN  NRP1  CYP2S1  CLEC10A  LRP6  RXFP1  MTMR11  GPR12  ST18  KIF17  FBLN2  PTPRM  TRIM71  AR  VSTM1  TGFBI  MARCO  CD1E  CLEC5A  PLBD1  DUSP27  CD14  GLT1D1  CD1C  DDIT4L  PRDM16  SLITRK4  FAM38B  HOXA6  CYP7B1  RANBP17  FLJ42875  COL4A5  **MYH11**  **(1.00)**  **CBFB**  **(1.00)** | LILRB4  ARHGEF10L  MS4A6A  UGGT2  FAM127A  C17orf55  NPR3  DNTT  ARPP21  SHD  NTRK1  C5orf23  OPALIN  HDC  UMODL1  UGT2B11 | ANK1  MYL4  VWDE  PLSCR4  CA1  PTK2  ADAMTS18  ZNHIT2  NKX2-3  GPR12  SORCS1  LPO  ACY3  SHANK1  **TP53**  **(1.00)** | IL17RE AADAT  SIX3 PROM1  FGF13 VNN3  PCBP3 C20orf200  PTPRG SLC8A3  NUDT10 DHRS9  CLTCL1 KIAA1598  LPO HOXA11AS  PRRT4 LDLRAD3  IGFBP2 HOXA4  S100B HOXB2  ASS1 MN1  IRX5 HOXB5  PPARG PRDM16  LTK HOXB6  ELANE HOXA11  GABRE CD109  PTGDS EREG  LOC399959 CPNE8  MPO TMEM105  STAB1 KIAA0087  COL23A1 HOXB3  KCNE1L FLJ42875  KRT17 NKX2-3  SLC24A3 SLITRK5  ANO7 HOXB4  CPA3 HTR1F  SLPI HOXA10  PRRG1 CNNM1  UGT3A2 HOXA9  MOSC2 MEIS1  LIN7A HOXA5  RETN HOXA3  CCNA1 HOXA6  ZNF711 NPR3  LAMC1 C5orf23  PRODH HTR7  ANXA8 VNN1  TDRD9 HOXA7  AZU1  CTSG  STOX2  MS4A3  KRT18  SLC28A3  EVPL  NDST3  BEND6  VSTM1  MEG3  FBN2  PTGER3  SHROOM4  IRX1  TEX15  AR  DDIT4L  **RARA**  **(1.00)**  **PML**  **(1.00)** | HOXB6  PHACTR3  HOXB5  APOC2  CT45A1  SCUBE1  WNT7B  NKX2-3  HOXB4  H2AFY2  HOXA11  HOXB3  RPS4Y1  C2  HOXB2  LOC728606  CCL23  HOXB7  HOXA7  HNMT  ZFY  CPNE8  SORT1  HOXA6  HOXA11AS  DDX3Y  HOXA3  UTY  PRKY  KDM5D  GTSF1  TLR8  MYO7A  OPALIN  GPR12  ZNF521  CLEC9A  CD34  TMIGD2  TRH  **DNMT3A**  **(0.71)**  **NPM1**  **(1.00)** | MYL4  AHSP  COL4A5  DLK1  JPH1  CLEC4C  GLI2  OPALIN  ALOX15B  BCORL2  SCN2A  TTTY15  EIF1AY  CYorf15B  USP9Y  RPS4Y1  UTY  DDX3Y  ZFY  DLGAP2  ANXA8  TNNT1  MARCO  LPHN3  LGALS2  CLEC10A  MEFV  **STAG2**  **(1.00)** | MSR1  TMEM176A  CD300E  MTMR11  TMEM176B  PDK4  TLR8  MS4A6A  CLEC7A  KYNU  DPP10  HOXB9  FGF13  GLI2  VAT1L  SNCAIP  CDH2  LOC399959  FLJ42875  CDH4  COL2A1  NUDT10  NKX2-3  GABRE  FERMT1  GPR85  PTPN20B  C20orf200  PTGFR  NDST3  DSG2  CSMD1  PAX8  PPARGC1A  APOC2  MPO  MOSC2  KRT8  PLCB4  LOC654433  BEND6  CYP7B1 | NKX2-3  HOXA5  HOXA3  SCARA3  HOXA6  HOXA4  FAM38B  LIN7A  HOXB3  ZNF521  LTBP1  SLC24A3  HOXA9  HOXB6  HOXA7  HOXB4  CPA3  MMP2  FAM110B  MEIS1  HOXA10  HOXB5  MDFI  DSC2  WNT7B  TRIM71  SIGLEC1  MPEG1  PTX4  PRLR  PTK2  KIF17  OLFML2A  MSR1  RETN  DUSP27  CACNA2D3  **NPM1**  **(1.00)** | UGT2B11  APBA1  HPGDS  SHD  C8orf79  CYP7B1  PROM1  SLITRK5  TRO  GPR173  HOXB6  KIAA0087  CES1  HOXB3  C20orf200  MEIS1  ELN  HOXB4  NKX2-3  HOXB5  KRT17  HOXA10  HOXA9  HOXA7  HOXA6  **CEBPA**  **(0.90)** | APP  CD34  MN1  PROM1  FAM171B  BAALC  GPR173  SDK2  HMGA2  LOC728606  APOC2 |

**References**

1. Reich M, Liefeld T, Gould J, Lerner J, Tamayo P, Mesirov JP (2006) GenePattern 2.0 Nature Genetics 38 no. 5 (2006): pp500-501 doi:10.1038/ng0506-500
